# Chemical Composition of Mexicali Propolis and Its Effect on Gastric Repair in an Indomethacin-Induced Gastric Injury Murine Model

**DOI:** 10.3390/antiox14010065

**Published:** 2025-01-08

**Authors:** Pilar Dominguez-Verano, Nadia Jacobo-Herrera, Andrés Castell-Rodríguez, Octavio Canales-Alvarez, Maria Margarita Canales-Martinez, Marco Aurelio Rodriguez-Monroy

**Affiliations:** 1 Posgrado en Ciencias Biológicas, Unidad de Posgrado, Edificio D, 1 Piso, Circuito de Posgrados, Ciudad Universitaria, Coyoacán, Mexico City 04510, Mexico; pilardomver@hotmail.com; 2 Laboratorio de Investigación Biomédica en Productos Naturales, Carrera de Medicina, UNAM, FES Iztacala, Avenida de los Barrios Número 1, Tlalnepantla de Baz 54090, Mexico; octaviocanalesa@gmail.com; 3 Unidad de Bioquímica Guillermo Soberón Acevedo, Instituto Nacional de Ciencias Médicas y Nutrición Salvador Zubirán. Avenida Vasco de Quiroga 14, Colonia Belisario Domínguez Sección XVI, Tlalpan, Mexico City 14080, Mexico; nadia.jacoboh@incmnsz.mx; 4 Departamento de Biología Celular y Tisular, Facultad de Medicina, Universidad Nacional Autónoma de México, Colonia. Universidad Nacional Autónoma de México, Coyoacán, Mexico City 04510, Mexico; castell@unam.mx; 5 Laboratorio de Farmacognosia, UBIPRO, UNAM, FES Iztacala, Avenida de los Barrios Número 1, Tlalnepantla de Baz 54090, Mexico; dra.margaritacanales@gmail.com

**Keywords:** propolis, peptic ulcer, gastric repair, antioxidant, anti—inflammatory, antiapoptotic, flavonoids

## Abstract

Propolis is a resinous substance produced by bees that has several biomedical properties that could contribute to the repair process of the gastric mucosa, such as antioxidant, anti-inflammatory, healing, and gastroprotective properties. Thus, this study aimed to determine the chemical composition of Mexicali propolis, its antioxidant capacity, and its effect on gastric repair. Three polarity-directed extracts were obtained: the ethanolic extract, the ethyl acetate extract, and the hexane extract. The antioxidant activity, total phenolic content (TPC), and flavone/flavonol content were determined for each extract. The chemical composition was analysed using HPLC—TOF—MS (High—Performance Liquid Chromatography—Time—Of—Flight Mass Spectrometry) and GC—MS (Gas Chromatography–Mass Spectrometry), and a total of 52 compounds were identified. The results revealed that the ethanolic extract had the greatest effect on free radical scavenging and the content of bioactive compounds. On the basis of these results, the effect of the Mexicali ethanolic extract of propolis (MeEEP) on gastric repair was subsequently evaluated. Prior to the evaluation, MeEEP was found to exhibit low oral toxicity, as determined under the Organisation for Economic Co-operation and Development (OECD) 425 guidelines. Gastric injury was induced in male C57BL/6 mice by intragastric administration of indomethacin (10 mg/kg). MeEEP (300 mg/kg) was administered 6 h after the induction of injury using indomethacin and daily thereafter. The mice were sacrificed at 12, 24, and 48 h to assess the effect. As a result, MeEEP enhanced the repair of the gastric lesion by decreasing the percentage of the bleeding area and attenuating the severity of histological damage, as demonstrated by H&E staining. This effect was associated with a reduction in MPO enzyme activity and in the levels of the proinflammatory cytokines TNF-α, IL-1β, and IL-6, maintaining controlled inflammation in gastric tissue. Furthermore, the administration of the extract increased SOD enzymatic activity and GSH levels, reducing the degree of oxidative damage in the gastric tissue, as demonstrated by low MDA levels. Finally, after evaluating the effect on apoptosis via immunohistochemistry, MeEEP was shown to reduce the expression of the proapoptotic marker Bax and increase the expression of the antiapoptotic marker Bcl-2. In conclusion, these findings suggest that MeEEP may enhance gastric repair through a cytoprotective mechanism by controlling inflammation exacerbation, reducing oxidative stress, and regulating apoptosis. These mechanisms are primarily attributed to the presence of pinocembrin, tectochrysin, chrysin, apigenin, naringenin, acacetin, genistein, and kaempferol. It is important to highlight that this study provides a preliminary exploration of the reparative effect of Mexican propolis, describing the potential mechanisms of action of the compounds present in Mexicali propolis.

## 1. Introduction

Propolis is a complex substance produced by bees by mixing wax and plant resins obtained from plant bark and buds. It has been used extensively in folk medicine given its various biomedical properties, including antioxidant, anti-inflammatory, immunomodulatory, gastroprotective, and healing activities. Its chemical composition, organoleptic characteristics, and biomedical properties have been described as dependent on the geographical environment, botanical origin, and seasonality [1,2]. To date, approximately 850 compounds have been detected in propolis from different regions of the world. These compounds include phenols, flavonoids, phenolic acids, terpenes, alcohols, fatty acids, and sugars [3], and a sample of propolis may contain 80–100 different compounds [4].

Propolis extracts are usually made from ethanol, water, chloroform, methanol, acetone, or ethyl acetate, with the ethanolic extract being the most commonly used. Nevertheless, it is important to note that the choice of solvent also influences the extracted compounds and the biomedical activity of the extract [5]. Several studies have shown that propolis extracts obtained with different solvents have protective or reparative effects on the gastric mucosa [6,7,8,9].

A peptic ulcer (PU) is an injury involving the rupture of the gastric mucosa that extends into deeper layers of the stomach, and the injury is accompanied by bleeding and inflammation of the injured area. This disease has a multifactorial origin, with the most common being *Helicobacter pylori* infection, the consumption of nonsteroidal anti-inflammatory drugs (NSAIDs), and stress. When these injuring factors overcome the protective mechanisms of the stomach, ulcers can occur in the mucosa. There is currently an increase in NSAID-induced PUs, with an estimated 10–30% of users developing lesions [10].

NSAIDs exert their ulcerogenic effect through both prostaglandin-dependent and prostaglandin-independent mechanisms. The first mechanism involves the inhibition of cyclooxygenases (COX-1 and COX-2), resulting in a reduction in prostaglandin synthesis and the mucus–bicarbonate barrier. The second is considered a topical effect, where NSAIDs increase the permeability of the mucosal surface, favour erosion, promote neutrophil adhesion, trigger death by necrosis and apoptosis, and induce oxidative stress [11]. The generation of reactive oxygen species (ROS) in the mucosa is associated with increased lipid peroxidation manifested by high levels of malondialdehyde (MDA) [12]. Oxidative stress is usually counteracted by the antioxidant defence system, which consists of the enzymes superoxide dismutase (SOD), catalase (CAT) and glutathione peroxidase (GPx), as well as nonprotein antioxidants such as glutathione (GSH) [13,14].

Similarly, the administration of NSAIDs leads to an increase in the levels of proinflammatory mediators, including cytokines such as TNF-α, IL-1β, and IL-6, which are fundamental in the process of damage and recovery [11]. TNF-α synthesis has been described as the primary event in the indomethacin-induced injury process. It acts on the gastric mucosa as a signal for the recruitment of neutrophils and macrophages, which then release proteolytic enzymes that damage the mucosa [15,16]. Once injury is established, NSAIDs inhibit restitution mechanisms essential for repair and subsequent remodelling events (cell proliferation and angiogenesis) [11]. In addition, the recurrence of injury is associated with persistent inflammation [16].

Current treatments for PUs focus on decreasing acid secretions, commonly proton pump inhibitors (PPIs), histamine H2 receptor antagonists, and antacids; however, these methods are often ineffective because they have significant adverse effects and do not adequately resolve mucosal lesions, and relapses frequently occur [17]; thus, there is great interest in the discovery of safe alternatives focused on the use of natural products [18].

It has been reported that propolis extracts from different regions have gastroprotective activity and promote the healing of gastric lesions induced in different models; however, each batch of propolis may have different repair mechanisms conferred by its chemical composition, which is unique depending on the area of collection [7,8,9]. There is currently moderate knowledge of the biomedical activities of propolis from Mexico [19], and it is not known whether it can promote gastric repair. Recent studies have reported the ability of propolis from Chihuahua to protect the gastric mucosa from injury [20] and to improve wound healing [21]. Both investigations revealed that its activity is due to the possible synergistic effect of its components, which reduces the severity of the lesions. Therefore, the purpose of this study was to characterize the chemical composition of Mexicali propolis, determine its antioxidant capacity, and evaluate its ability to repair the gastric mucosa in an acute injury model.

## 2. Materials and Methods

### 2.1. Propolis

The propolis sample was obtained from the Colorado apiary in February 2021 by beekeepers Guillermo Antionio Ruvalcaba and Lucero Castañeda. The apiary is located in the municipality of Mexicali, BC, Mexico. The organoleptic characteristics of Mexicali propolis (Table 1) have been described according to the parameters published in the Official Mexican Norm NOM-003-SAG/GAN-2016 [22].

### 2.2. Chemicals and Drugs

Indomethacin was purchased from Sigma—Aldrich and kept at room temperature. A Superoxide Dismutase Assay Kit (Item No. 706002) was obtained from Cayman Chemical (Ann Arbor, MI, USA). A Glutathione Assay Kit (Item No. 703002) was obtained from Cayman Chemical (Ann Arbor, MI, USA).

The Myeloperoxidase (MPO) Activity Assay Kit (Colorimetric) (ab105136), the Lipid Peroxidation (MDA) Assay Kit (Colorimetric) (ab233471), the Mouse TNF alpha ELISA Kit (ab285327), the Mouse IL-1 beta ELISA Kit (ab197742), and the Mouse IL-6 ELISA Kit (ab100713) were obtained from Abcam (Waltham, MA, USA). A protease inhibitor cocktail (Complete^TM^ Mini) from Roche (Mannheim, Baden-Wurtemberg, Germany), Folin–Ciocalteu reagent, 2,2-diphenyl-1-picrylhydrazyl (DPPH), bis-(trimethylsilyl) trifluoroacetamide (BSTFA) gallic acid, tween 20, sodium carbonate, methanol, ethanol, acetone, ethyl acetate, dichloromethane, hexane, and pyridine were purchased from Sigma—Aldrich (St. Louis, MO, USA). Hydrocarbon standards of 1-octene and octadecane and flavonoid standards (Trolox, genistein, kaempferol, acacetin, apigenin, chrysin, pinocembrin, myricetin, caffeic acid, naringenin, naringin, quercetin, baicalein, and catechin) were purchased from Sigma—Aldrich (St. Louis, MO, USA).

### 2.3. Extraction and Fractionation

**Figure 1:**
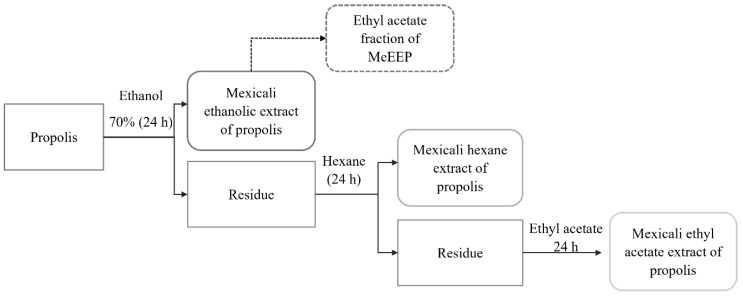
Process of obtaining the extracts of Mexicali propolis and fractionation for chemical characterization.

The extracts of Mexicali propolis were obtained using the maceration method. First, 50 g of propolis was dissolved in 500 mL of 70% ethanol for 24 h at room temperature. Next, the supernatant was filtered to extract the polar fraction. The residue was then macerated with hexane (500 mL) for 24 h to obtain the nonpolar fraction. The residue of this second extraction was dissolved in ethyl acetate (500 mL) to extract the compounds of intermediate polarity. The three extractions were filtered and distilled under reducing pressure in a rotatory evaporator. The three extracts were placed in glass containers to evaporate the solvent completely. Finally, TLC analysis was performed to identify the mobile phase that efficiently separated the MeEEP. Five mobile phases from least polar to the most polar were tested via TLC strips (5 cm × 1 cm) of silica gel on an aluminium support with the fluorescence indicator F254 from Merck KGaA (Darmstadt, Hesse, Germany): hexane, hexane–ethyl acetate (1:1), ethyl acetate, ethyl acetate–methanol (1:1), and methanol. After the most suitable mobile phase (ethyl acetate) was identified, MeEEP was fractionated with ethyl acetate (EAF). All the chromatographs were visualized and analysed under ultraviolet light (UVL) at 365 and 256 nm (Figure 1).

The yield of the extracts was calculated as the ratio between the weight of the extract obtained and the weight of the unprocessed propolis using the following equation:

Yield % = [(Weight of extract/Weight of unprocessed propolis)] × 100(1)

For the fractions, the ratio between the weight of each fraction and the weight of the extract from which it was derived was used.

### 2.4. Fractionation Using Preparative Thin-Layer Chromatography and Analysis Using Thin-Layer Chromatography (TLC)

Prep-TLC plates of silica gel (SIL G-200) with the fluorescence indicator F254 and dimensions of 20 cm × 20 cm and 2 mm thickness on a glass support from Macherey-Nagel (Düren, North Rhine-Westphalia, Germany) were used. One hundred milligrams of EAF was seeded on Prep-TLC, and hexane–ethyl acetate (3:7) was used as the mobile phase. The bands were identified under UVL at 256 and 365 nm. Then, the bands were removed from the plate and extracted with methanol. All the fractions were analysed via TLC strips (5 cm × 1 cm).

### 2.5. Analysis Using HPLC—TOF—MS

For this analysis, the following equipment conditions were established, as described previously [23]: “HPLC-TOF-MS analysis was performed using an Agilent 1200 Infinity LC liquid chromatograph coupled with an Agilent 6230 TOF Time—of—Flight mass spectrometer. The mass spectrometer was equipped with an electrospray ionization (ESI) source (ESI 5614289023) and operated with Mass Hunter Workstation software, version B.05.01, build 5.01.5125.3 with a negative ionization mode. The capillary voltage was 3500 V, and the dry gas temperature was 250–300 °C. Nitrogen was used as the dry gas at a flow rate of 6 L/min. The nebulizer pressure was 60 psig, and the fragmentation voltage was 200 V. The mass range was 50–1000 m/z. The mass acquisition rate was 1 spectrum/s. Chromatographic separation was performed via an HPLC system (Infinity Series 1200, Agilent Technologies, Waldbronn, Baden-Wurtemberg, Germany) equipped with a Kinetex column of 150 m length, 2.1 mm diameter, 2.6 μm particle size, 100 A pore size and C-18 phase (Phenomenex, Torrance, CA, USA). A two-phase gradient was used: Phase A (water with 1% formic acid) and Phase B (pure acetonitrile). The flow rate was set at a constant 2 mL/min at a constant pressure of 600 bar. The elution gradient programmed started with 80% A and 20% B and changed to 70% A and 30% B after 10 min. At 30 min, it was modified to 60% A and 40% B and maintained until 50 min. At 65 min, the proportions were modified to 30% A and 70% B. At 70 min and 80 min, a proportion of 0% A and 100% was maintained. The total time was 80 min”. MeEEP, fractions of EAF, and 15 standards (genistein, kaempferol, acacetin, apigenin, chrysin, pinocembrin, myricetin, caffeic acid, naringenin, naringin, quercetin, baicalein, and vanillin, epicatechin, and catechin) were injected, each in a volume of 20 μL (1 mg/mL).

### 2.6. Derivatization and GC—MS

For GC—MS analysis, the silylation derivatization method was used to improve the detection of the compounds. The same method and equipment conditions were used as described previously [23]: “Five milligrams of extract was placed into a glass tube (one each for extract) and mixed with 50 μL of pyridine and 75 μL of bis(trimethylsilyl) trifluoroacetamide (BSTFA). The tube containing the sample was sealed and heated at 100 °C for 60 min, after which the solvent residue was evaporated and dissolved in 500 μL of HPLC-grade hexane. One microlitre of this solution was injected into the GC—MS instrument. The conditions of the equipment were as follows. An Agilent Technologies 6850 Network GC System gas chromatograph coupled to a Model 5975C mass spectrometer equipped with HP-5MS columns that were 30 m long, 0.25 mm inner diameter, and 0.25 μm thick (Agilent Technologies) was used. The set temperature ranged from 100 to 300 °C with an increase rate of 5 °C/min, and helium was used as the carrier gas with a run flow rate of 0.7 mL/min. The injection mode was split at a ratio of 1:20, with an Injector temperature of 280 °C” The’mass range detected was 35 to 600 m/z with an ionization voltage of 70 eV and an interface temperature of 300 °C. The total run time was 40 min. In addition, 1—octene and octadecane were injected as hydrocarbon standards”. Compound identification was carried out using the NIST 8.0 database. The equipment compared the identified compound’s retention time, mass spectrum, and molecular ion with the database standard.

### 2.7. Antioxidant Capacity Determination and TLC—DPPH Bioautography Method

Antioxidant capacity was determined by the method of reducing the radical 2,2-diphenyl-1-picrylhydracil (DPPH). A total of 50 μL of extract at different concentrations (10, 20, 30, 40, 50, 60, 70, 80, 90 100, 150, 200, 250, 300 and 1000 μg/mL) and 150 μL of the DPPH solution (100 μM) were placed in a 96-well plate. After 30 min of incubation at 37 °C, absorbance was measured at 540 nm using a MultiskanTM SkyHigh microplate spectrophotometer (Thermo Fisher Scientific Inc., Regis Singapore, Singapore). Quercetin was used as a standard. The half-maximal effective concentration (EC_50_) of MeEEP, MeEAEP, and MeHEP was determined. The percent inhibition of DPPH was calculated via the following equation:

DPPH inhibition % = [(absorbance of the control − absorbance of the sample)/absorbance of the control] × 100(2)

To identify the fractions with antioxidant capacity, the TLC—DPPH bioautography technique was used. On a sectioned TLC plate, 10 μL of each fraction and standard (caffeic acid and quercetin) was added at a concentration of 1 mg/mL. The plate was subsequently sprayed with a DPPH solution (100 μM) and incubated for 30 min at room temperature. The active samples appear as yellow spots on a violet background under visible light [24]. The percentage of inhibition of DPPH radical activity by each fraction was determined at a concentration of 1 mg/mL.

### 2.8. Total Phenolic Content (TPC)

The total phenolic content was determined via a gallic acid standard curve (0.00125, 0.0025, 0.005, 0.01 and 0.02 mg/mL) from a stock solution of 0.2 mg/mL. A volume of 25 μL of the extract was obtained from a stock solution of 5 mg/mL. For this purpose, each aliquot (extract and gallic acid) was brought to a final volume of 1 mL with distilled water. Then, 700 μL of distilled water and 50 μL of Folin–Ciocalteu reagent were added. After 5 min, 150 μL of sodium carbonate (200 g/L Na_2_CO_3_) was added. After two hours of incubation at room temperature, 200 µL of each sample was placed in a 96-well plate, and the absorbance was read on a plate reader at 760 nm. The results are expressed as milligrams of gallic acid equivalents per gram of extract (mg GAE/g).

### 2.9. Flavone/Flavonol Content

Flavone/flavonol content was determined via a quercetin standard curve (1–100 μg/mL). The extract was used at a concentration of 10 mg/mL. To prepare this mixture, 1 mL of sample was mixed with 1 mL of 2% aluminium chloride (AlCl_3_). Two hundred microlitres of the mixture was placed in a 96—well plate, and after 10 min, the absorbance was read in a plate reader at 425 nm. The results are expressed as milligrams of quercetin equivalents per gram of extract (mg QE/g).

### 2.10. Animals

Male C57BL/6 mice that were 6 weeks of age and weighed 20 ± 2 g (gastric lesion protocol) and CD1 mice that were 7 weeks of age and weighed 30 ± 5 g (oral toxicity assay) were used (*Mus musculus)*. The mice were obtained and maintained in the animal facility of the FES Iztacala, UNAM, in a pathogen-free environment, according to the guidelines of the Norma Oficial Mexicana NOM-052-ZOO-1999, Especificaciones técnicas para la producción, cuidado y uso de los animales de laboratorio, Secretaría de Agricultura, Ciudad de México. This study was approved by the bioethics committee of the FES Iztacala, UNAM (CE/FESI/072023/1628). The mice were housed in polycarbonate cages with free access to water and food under a light–dark cycle at a temperature of 25 °C. Each cage contained five mice. Mice were randomly assigned for all tests, with each mouse being considered an experimental unit. All the mice were acclimatized for one week before the experiments.

### 2.11. Acute Oral Toxicity

An acute toxicity limit test was performed on the basis of the 425 protocol described by the Organization for Economic Cooperation and Development (OECD) for testing chemicals [25]. Five seven-week-old male CD1 mice were selected and fasted for 2 h with free access to water. A single dose of 2000 mg/kg MeEEP was administered in a volume of 200 µL. Signs, mortality, and activity were monitored for each mouse during the first 30 min, hourly for the next 4 h, and every 24 h for 14 days. For 14 days, the mice were maintained with free access to water and food under normal temperature and humidity conditions and a light–dark cycle.

### 2.12. Indomethacin-Induced Gastric Injury in Mice

To evaluate the reparative effect on the gastric mucosa, three experimental groups were established: the control group (*n* = 5), the indomethacin group (*n* = 20), and the MeEEP group (300 mg/kg, *n* = 15). The mice were randomly assigned to each experimental group. Prior to the experiment, the mice fasted for 12 h with free access to water. The control group was subsequently administered 200 μL of vehicle (Tween 20 at 2%). Afterwards, gastric injury was induced by administering a dose of indomethacin (10 mg/kg) orally to the indomethacin group and the MeEEP group. The mice were left to interact with the indomethacin for 6 h, then for the indomethacin group, 200 µL of the vehicle (Tween 20 at 2%) was administered, and for the MeEEP group, a dose of 300 mg/kg of MeEEP was administered, both daily and orally. After the first dose of vehicle or extract was administered to the experimental groups, the mice had free access to water and food.

For the control group, samples were obtained 6 h after vehicle administration. For the indomethacin group, samples were obtained at 6, 12, 24, and 48 h (5 mice for each period). For the MeEEP group, samples were obtained at 12, 24, and 48 h (5 mice for each period). To obtain stomach samples for each evaluation period, the mice were sacrificed in a CO_2_ chamber, and the stomachs were removed for cleaning and analysis. The stomachs were opened along the greater curvature, cleaned with cold PBS, and then spread on a flat surface. The internal area of the stomach was photographed with a Celestron handheld digital microscope (Torrance, CA, USA). The images were analysed with ImageJ Software v.2.14.0/1.54f to obtain the percentage of the bleeding area (macroscopic analysis).

### 2.13. Histological Analysis

To assess the histological integrity of the gastric mucosa, the stomachs were fixed in 10% formaldehyde dissolved in PBS at pH 7.4, followed by the histological technique of dehydration in alcohol and embedding in paraffin. Then, 5 μm tissue sections were cut on a microtome and stained with haematoxylin and eosin.

To assess histopathological changes in the gastric tissue, the histological damage index (HDI) of gastric injury was calculated with slight modifications [26,27]. Seven parameters (inflammatory infiltrate, oedema, vessel congestion, haemorrhage, erosion, necrosis/apoptosis, and the presence of ulceration) were evaluated. The values assigned were from 0 to 4 in order of severity, and the sum of all the parameters was the final value of the index.

### 2.14. Immunohistochemistry

Apoptosis in gastric tissue was determined according to the immunohistochemistry protocol described briefly below. Sections of 5 μm thick paraffin—embedded tissue were cut, and subsequently, the paraffin was removed by rinsing with xylene. A Revelar Decloaker (RV1000M: Biocare Medical, Concord, CA, USA) was used for antigen retrieval. Then, to block peroxidase activity and nonspecific binding sites, the tissue sections were spiked with hydrogen peroxide and 0.1% albumin for 10 min each. The primary antibodies anti—Bax (ab81083; Abcam), a proapoptotic marker, and anti—Bcl-2 (ab196495; Abcam), an antiapoptotic marker, were used. The MACH 2 rabbit HRP—polymer (RHRP520G; Biocare Medical) was used as the secondary antibody, and the IntelliPATH FLXTM DAB Chromogen Kit (IPK5010G80: Biocare Medical) was used as the chromogen. Finally, the tissue sections were rinsed with PBS to stop the reactions, stained with haematoxylin, dehydrated, and mounted.

### 2.15. Determination of the Oxidative Stress Parameters SOD, MDA, and GSH

SOD enzyme activity and GSH and MDA levels were determined in gastric tissue homogenates via colorimetric kits according to the manufacturers’ recommendations. To obtain homogenates, the stomachs of the different experimental groups were opened at the greater curvature and cleaned with cold PBS (pH 7.0–7.2). Then, the stomachs were homogenized with a protease inhibitor (Complete^TM^ Mini) from Roche (Mannheim, Baden-Wurtemberg, Germany), dissolved in cold PBS using a Bullet Blender model BBX24 (New York, NY, USA). The homogenate was subsequently centrifuged at 13,400 rpm for 10 min, and the resulting supernatants were stored at −70 °C.

### 2.16. Determination of MPO Activity and Cytokine Levels

Myeloperoxidase (MPO) activity and cytokine levels (TNF-α, IL-1β, and IL-6) were determined in gastric tissue homogenate via a colorimetric kit according to the manufacturer’s instructions.

### 2.17. Statistical Analysis

The data for all tests are expressed as the means ± SDs. Statistical differences between the MeEEP group and the indomethacin group (comparing all time periods) were determined via an unpaired *t* test (percentage of bleeding area; histological damage index; SOD and MPO enzyme activity; and GSH, MDA, TNF-α, IL-1β, and IL-6 levels). Statistical differences between the control group, indomethacin group, and MeEEP group (48 h time) were tested using one-way analysis of variance (ANOVA) (SOD and MPO enzyme activity; GSH, MDA, TNF-α, IL-1β and IL-6 levels; and immunohistochemistry). The data were evaluated via GraphPad Prism 9 software v.9.0.0. A value of *p* ˂ 0.05 was considered statistically significant.

## 3. Results

### 3.1. Chemical Characterization of Mexicali Propolis

#### 3.1.1. Extraction Yield

To determine the chemical composition of Mexicali propolis, polarity-guided extraction was performed using 70% ethanol, ethyl acetate, and hexane. Each extract had a different yield depending on the polarity of the solvent used. The ethanolic extract had a yield of 28.4%, the ethyl acetate extract had a yield of 8.05%, and the hexanoic extract had a yield of 27.05%.

**Figure 2:**
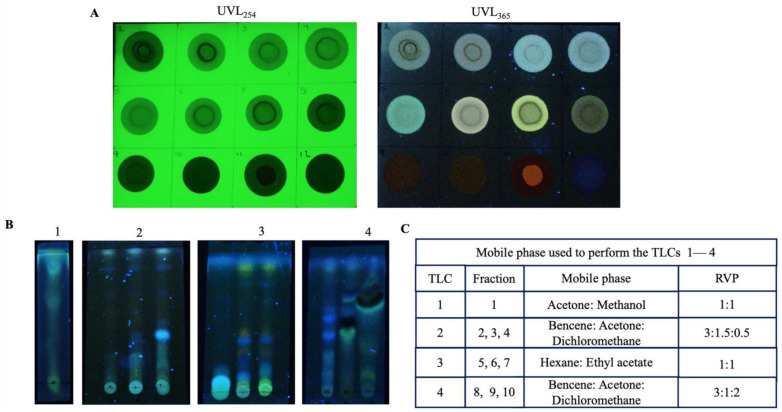
Thin-layer chromatograms of the EAF fractions of MeEEP. (**A**) Fractions visualized under ultraviolet light (UVL 254 and 365 nm). Fractions, 1–10; reference standards, 11–12 (11—quercetin; 12—caffeic acid). (**B**) Fractions developed with the indicated mobile phase and visualized under UVL 365 nm. (**C**) Mobile phases used to perform fractionation using TLC.

The MeEEP was subsequently fractionated with the aim of obtaining fewer complex samples to determine the chemical composition. To achieve this, thin-layer chromatography and preparative thin-layer chromatography analysis were performed. From the TLC analysis, the ethyl acetate mobile phase was the most suitable, allowing for the formation of defined bands. Therefore, fractionation with ethyl acetate was performed, with a yield of 87.72% (EAF). Preparative TLC was performed with 100 mg of EAF, resulting in 10 fractions. The ten fractions and the reference standards (quercetin and caffeic acid) were visualized under ultraviolet light at 254 and 365 nm to corroborate that each fraction consists of compounds with double bonds and conjugated double bonds. At 254 nm, the presence of these compounds was observed as dark spots (Figure 2A), and at 365 nm, each fraction emitted a blue, green, or yellow fluorescence (Figure 2B), characteristic of phenolic compounds. Furthermore, the fractions were developed in a TLC system using the most appropriate mobile phase to resolve the chemical complexity of each fraction. This allowed the fractions to be clearly visualized in separate and defined bands, from the most polar (fraction 1) to the least polar (fraction 10), corresponding to the compounds that constitute them and confirming that the fractions are distinct. The total yields of the extracts and fractions are presented in Table 2.

#### 3.1.2. Compounds Identified by HPLC—TOF—MS in the Ethanolic Extract of Mexicali Propolis

MeEEP and the 10 fractions obtained by prep—TLC were analysed using HPLC—TOF—MS. Each compound was determined by comparing the mass spectrum and the retention time of the standard with those of the compound registered by the equipment. As a result, seven compounds belonging to the flavonoid group were identified. The characteristics of the compounds are described in Table 3. The chromatograms of MeEEP and the fractions obtained from EAF are shown in the Supplementary Material (Figure S1).

#### 3.1.3. Compounds Present in Mexicali Propolis Identified by GC—MS

To perform GC—MS analysis, MeEEP, MeHEP, MeEAEP, and EAF were derivatized via the silylation method with the aim of increasing the degree of chemical characterization. Each compound was determined via comparing the characteristics of the compound registered by the equipment, including retention time, molecular ion, and mass spectrum, with those in the NIST 8.0 database. From the GC—MS analysis, a total of 45 compounds were identified in Mexicali propolis. All the compounds are reported in Table 4, Table 5, Table 6 and Table 7. The chromatograms of each extract and the mass spectra of each identified compound are presented in the Supplementary Material (Figure S2). Some compounds were detected in more than one extract.

#### 3.1.4. Biomedical Properties Reported for the Compounds Identified in Mexicali Propolis

A total of 52 compounds were identified through both analyses (HPLC—TOF—MS and GC—MS). A literature search was conducted to identify the biomedical activities reported for these compounds. Twenty—nine compounds were identified as having one or more of the activities described in Table 8. These activities could contribute to a decrease in gastric injury, which is the subject of interest for this study.

### 3.2. Antioxidant Capacity of Mexicali Propolis

Antioxidant capacity contributes to the protection of tissues against free radical damage and is a property of great biomedical importance linked to chemical composition, especially the contents of phenols and flavonoids [93]. The DPPH method was used to evaluate the antioxidant capacity of the extracts and fractions. The EC_50_ values of the extracts were evaluated. MeEEP and MeEAEP had EC_50_ values of 112.16 ± 3.58 and 1180 ± 29.40 μg/mL, respectively, but it was not possible to determine the EC_50_ values of MeHEP.

**Figure 3:**
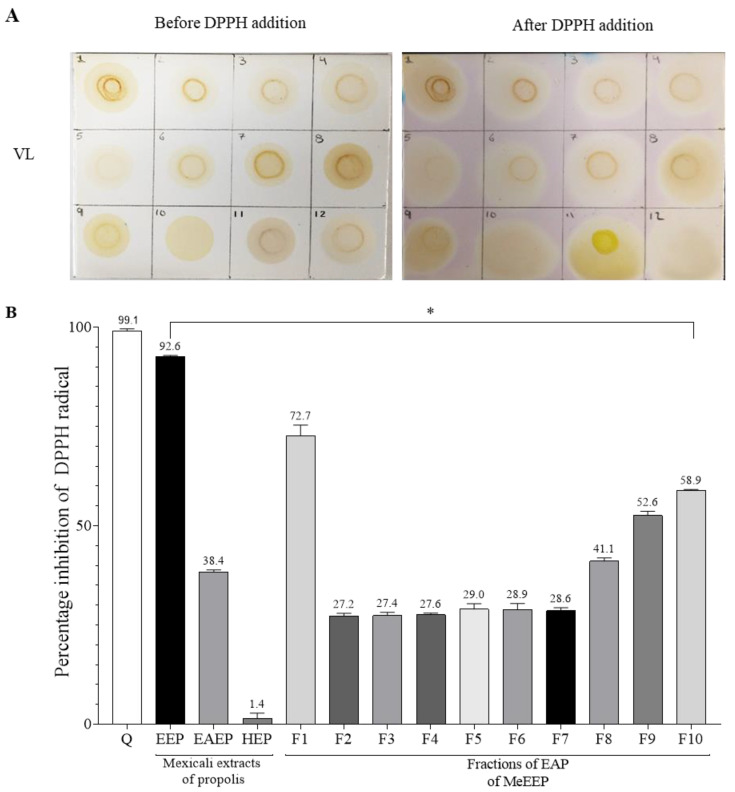
Antioxidant capacity of fractions of the EAF and Mexicali propolis extracts. (**A**) Representative photograph of the TLC—DPPH bioautography of fractions visualized under visible light (VL) before and after DPPH application. Fractions (1–10), quercetin (11), and caffeic acid (12). (**B**) Percentage inhibition of DPPH radical. Quercetin (control) (Q) Mexicali propolis extracts (EEP, EAEP, and HEP), and fractions (F1-F10). Group comparisons were determined using one-way ANOVA. A value of *p* < 0.05 was considered statistically significant. * Significant differences relative to control.

Subsequently, via the TLC—DPPH bioautography method, it was determined which of the fractions provides the antioxidant capacity to MeEEP. As all fractions tested positive for antioxidant capacity (represented by the colour change of DPPH from violet to yellow) (Figure 3A), we decided to quantify the percentage inhibition of the DPPH radical. This analysis was performed for the ten fractions and the three extracts (MeEEP, MeEAEP, and MeHEP) at a concentration of 1 mg/mL. As a result, each fraction presented a different percentage of inhibition; however, no fraction was more efficient that the complete ethanolic extract, which presented the highest percentage inhibition of the DPPH radical of 92.6% (Figure 3B).

In addition, the total phenolic content and flavone/flavonol content of the three extracts (MeEEP, MeEAEP, and MeHEP) were determined. The ethanolic extract had the highest TPC (36.8%) and flavone/flavonol content (2.58%), as shown in Table 9. These results show that the ethanolic extract holds promise in terms of evaluating its effect on a gastric lesion model.

### 3.3. Acute Oral Toxicity of MeEEP

The acute oral toxicity of MeEEP was evaluated according to the guidelines of the OECD protocol 425. Toxicity parameters were observed during the first 30 min, then at 4 h and once daily for 14 days. All parameters are reported for each mouse. The dose evaluated was 2000 mg/kg, which is known as the limit dose. The analysis revealed that MeEEP did not produce any evidence of acute oral toxicity in any of the mice; therefore, MeEEP was classified as a substance with a relatively low risk of acute toxicity or no toxic effect, i.e., category 5 of the Globally Harmonized System (GHS) of Classification and Labelling of Chemicals. Doses below the dose limit are considered appropriate for assessment.

### 3.4. Ethanolic Extract of Mexicali Propolis Attenuated Gastric Lesions Produced by Indomethacin at the Macroscopic Level

To evaluate the effect of MeEEP on the gastric repair model, a dose of 300 mg/kg was used, which was established on the basis of the results obtained from the limit dose and taking into consideration the doses reported in gastroprotection models [20,94]. The following results were obtained.

**Figure 4:**
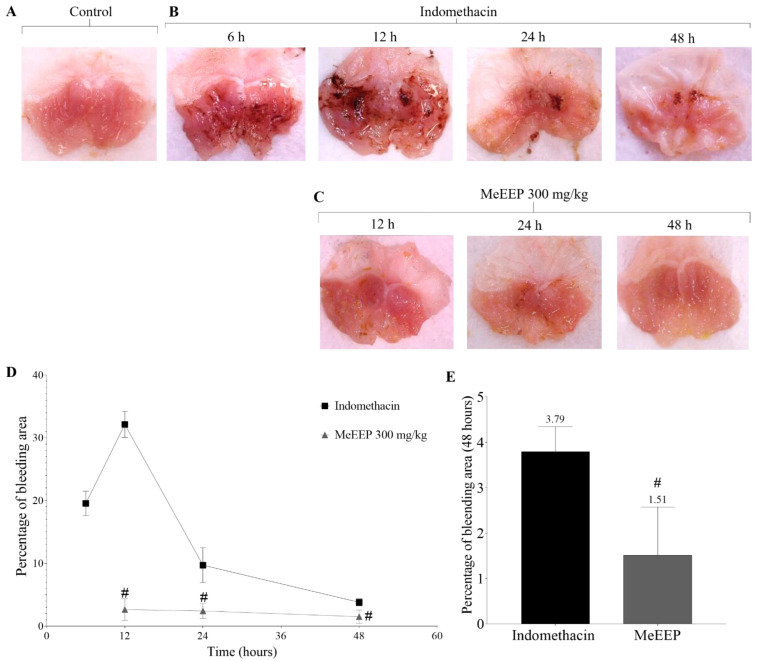
Macroscopic assessment of the effect of MeEEP on indomethacin—induced gastric lesions. (**A**) Control group with intact mucosa and no lesions. (**B**) Indomethacin group with haemorrhage and erythema (6, 12, 24, and 48 h). (**C**) MeEEP group treated daily at a dose of 300 mg/kg. A decrease in lesion severity was observed at all times, with mild erosion (12, 24, and 48 h). (**D**) Percentage of bleeding area in the indomethacin and MeEEP groups. The indomethacin group presented an initial percentage of bleeding area of 19.6% at 6 h and a peak percentage of lesions of 32.1% at 12 h. This value decreased to 9.7% at 24 h and 3.8% at 48 h. MeEEP was administered at 6 h, and at 12 h, the extract decreased the bleeding area to 2.6%, with a decrease of 2.4% at 24 h and 1.5% at 48 h. (**E**) Percentage of the bleeding area at 48 h. Values are shown as means ± SDs. Comparisons between two groups were analysed via Student’s *t* test. *p* ˂ 0.05 was considered statistically significant. # Significant differences with respect to the indomethacin group.

The macroscopic analysis of stomach samples revealed that the control group had an intact mucosa with no superficial damage (Figure 4A), whereas the indomethacin group presented an irritated mucosa with haemorrhage, erythema, and petechiae after 6 h. The bleeding area increased after 12 h, reaching a maximum peak of injury. Subsequently, the bleeding area decreased with time (24 and 48 h), but small areas of whitish lesions remained present (Figure 4B). The establishment and course of the lesion were represented based on the percentage of the bleeding area, using 100% of the total area of the mucosa as a reference and plotting the percentage that represent the bleeding area, which was delimited by ImageJ software v.2.14.0/1.54f (Figure 4D). Analysis revealed that the percentage of the area affected by the lesion was 19.6% at 6 h. At 12 h, the percentage increased to 32.1%, and this value subsequently decreased over time.

In contrast, when MeEEP was administered, the percentage of the bleeding area at 12 h decreased from 32.1% (indomethacin group) to 2.6% (MeEEP group), and this effect remained at 24 and 48 h, with the minimum percentage of the bleeding area at 48 h remaining below that of the indomethacin group (Figure 4C,E). Therefore, MeEEP attenuates indomethacin-induced injury in a short period of time.

### 3.5. Histological Analysis

**Figure 5:**
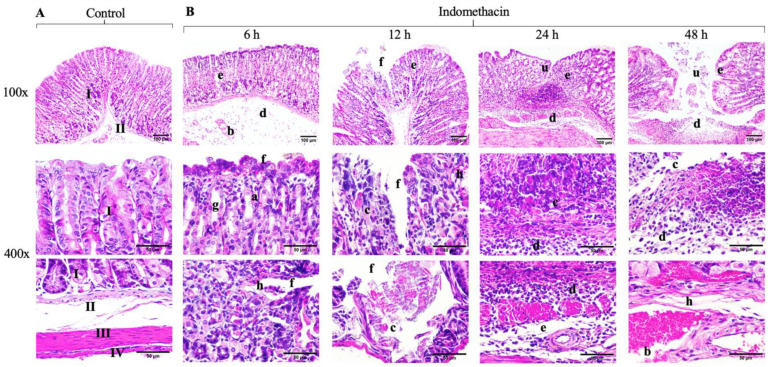
Histological analysis of the effects of the ethanolic extract of Mexicali propolis (300 mg/kg) on gastric repair. (**A**) Photomicrographs of the control group without any lesions. (**B**) Photomicrographs of the indomethacin group at 6, 12, 24, and 48 h, showing the course of establishment and the severity of the lesion generated (H&E staining). Mucosa (I), submucosa (II), muscularis (III), serosa (IV), apoptosis (a), dilated blood vessels (b), necrotic cells (c), inflammatory infiltrate (d), oedema (e), erosion (f), enlarged glands (g), haemorrhage (h), and ulcers (u).

The analysis of the experimental groups revealed that the control group had intact mucosa without any lesions (Figure 5A). The indomethacin group showed gradual histological damage, demonstrating the establishment of the lesion at 6 h with erosion on the mucosal surface, haemorrhage, and oedema in the submucosa. At 12 h, the degree of injury increased, starting with rupture of the mucosal layer caused by increased apoptosis, necrosis, and superficial erosion. In severe cases, the loss of mucosal substances with adjacent tissue with oedema and inflammatory infiltration was observed, which is characteristic of acute NSAID injury.

At 24 h, the histological damage patterns were more evident. The ulcers in the tissue were small and extended over the mucosal layer, with slight accumulation of inflammatory infiltrates in the ulcer base as well as congested blood vessels and necrotic cells at the edge of the ulcer. In addition, in response to increased oedema, the mucosa became hyperaemic. On the other hand, recovering lesions were observed with a continuous surface; however, the base was covered with inflammatory infiltrate, an area susceptible to relapse. At 48 h, severe lesions were identified, probably due to relapses, involving the submucosal and muscular layers with congested blood vessels in the submucosa and necrotic tissue (Figure 5B).

**Figure 6:**
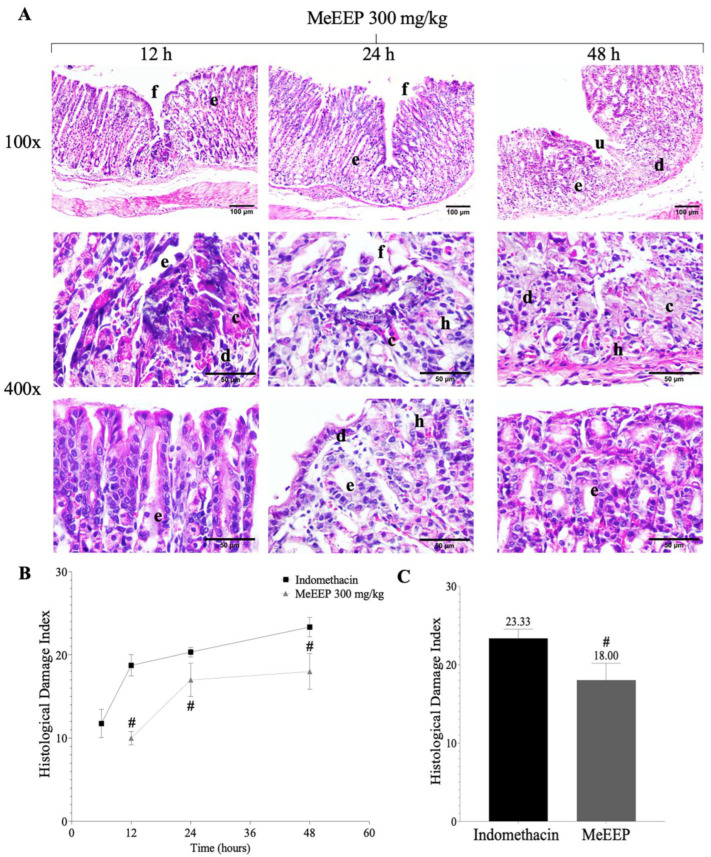
Histological analysis of the effects of the ethanolic extract of Mexicali propolis (300 mg/kg) on gastric repair. (**A**) Sequence of photomicrographs of MeEEP’s effect at 12, 24, and 48 h (H&E staining). (**B**) Histological damage index. (**C**) Histological damage index at 48 h. Values are shown as means ± SDs. Comparisons between two groups were analysed via Student’s *t* test. *p* ˂ 0.05 was considered statistically significant. # Significant differences with respect to the indomethacin group. Mucosa (I), submucosa (II), muscular (III), serosa (IV), necrotic cells (c), inflammatory infiltrate (d), oedema (e), erosion (f), haemorrhage (h), and ulcers (u).

In contrast, the group treated with MeEEP (300 mg/kg) presented a less severe course of injury. Administering the extract 6 h after the lesion had been generated indicated that its effect was based on a pre-existing lesion. The results revealed that at 12 h, minor superficial lesions with mild oedema and congested blood vessels were present. At 24 h, a moderate lesion course was observed. Compared with those in the indomethacin group, the lesions generated at baseline were significantly reduced at 48 h. MeEEP prevented lesions from intensifying by maintaining inflammation and oedema at low levels, safeguarding the integrity of the gastric mucosa, and allowing the lesion to resolve (Figure 6A,C). These microscopic changes are reflected in the histological damage index (Figure 6B).

### 3.6. Ethanolic Extract of Mexicali Propolis Decreases MPO Activity and Proinflammatory Cytokine Levels

**Figure 7:**
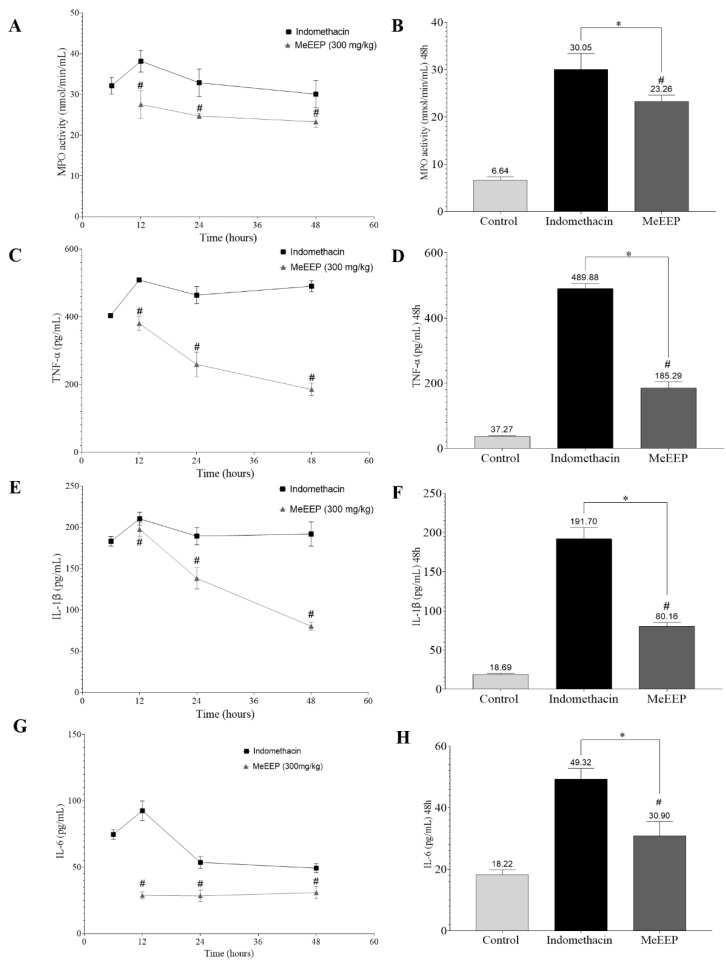
Effect of ethanolic extract of Mexicali propolis on the levels of MPO and the proinflammatory cytokines TNF-α, IL-1β, and IL-6. (**A**) MPO enzyme activity levels in the indomethacin and MeEEP groups (6, 12, 24, and 48 h). (**B**) MPO activity at 48 h. Cytokine levels of (**C**) TNF-α, (**E**) IL-1β, and (**G**) IL-6 in the indomethacin and MeEEP groups. Cytokine levels at 48 h: (**D**) TNF-α, (**F**) IL-1β, and (**H**) IL-6. Values are shown as means ± SDs. Comparisons between two groups were analysed via Student’s *t* test. Comparisons between more groups were determined using one-way analysis of variance (ANOVA). *p* ˂ 0.05 was considered statistically significant. * Indicates significant differences with respect to the control group. # Indicates significant differences with respect to the indomethacin group.

Indomethacin—induced gastric injury triggers an increased inflammatory response. MPO is an indicator of the acute inflammatory response, and the increased production of the cytokines TNF-α, IL-1β, and IL-6 is also implicated in the development of gastric injury. As a result, the control group exhibited a low level of MPO activity (6.64 nmol/min/mL). Compared with the control group, the indomethacin group presented increased MPO activity at all time points tested. Compared with that in the indomethacin group, MPO activity in the MeEEP group was reduced at all time points tested (Figure 7A). The levels of cytokines (TNF-α, IL-1β and IL-6) also increased significantly in the indomethacin group, indicating severe injury. However, MeEEP treatment decreased TNF-α, IL-1β, and IL-6 levels (Figure 7C,E,G). This reduction in the levels of inflammatory parameters was noticeable 48 h after injury (Figure 7 B,D,F,H). These findings indicate that the ability of the treatment to reduce inflammation allows a faster recovery from the injury.

### 3.7. Ethanolic Extract of Mexicali Propolis Increases the Parameters of the Endogenous Antioxidant Defence System and Decreases the Levels of MDA

During the formation of gastric lesions, there is an increase in reactive oxygen species, which cause cell and tissue damage characterized by membrane lipid peroxidation and MDA formation. This oxidative damage is mediated by endogenous antioxidants such as SOD and GSH, which neutralize free radicals and protect against oxidative stress [95,96].

**Figure 8:**
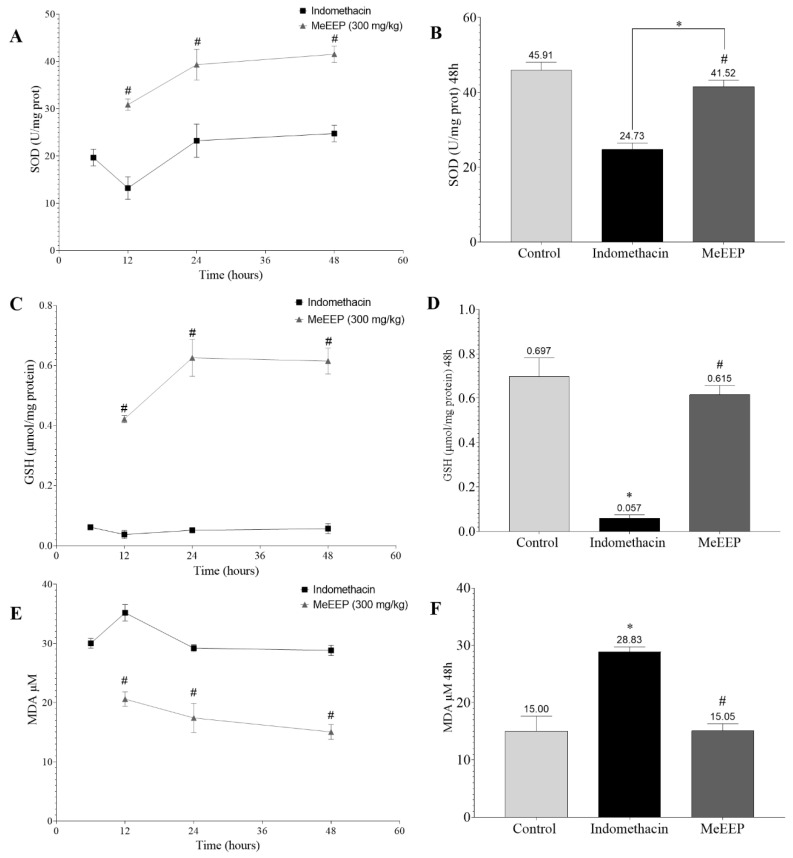
Effects of MeEEP on the oxidative stress parameters of SOD activity and MDA and GSH levels. (**A**) SOD enzyme activity in the indomethacin and MeEEP groups (6, 12, 24, and 48 h). (**B**) SOD activity at 48 h. (**C**) GSH levels in the indomethacin and MeEEP groups (6, 12, 24, and 48 h). (**D**) GSH levels at 48 h. (**E**) MDA levels in the indomethacin and MeEEP groups (6, 12, 24, and 48 h). (**F**) MDA levels at 48 h. Values are shown as means ± SDs. Comparisons between two groups were analysed via Student’s *t* test. Comparisons between more groups were determined using one-way analysis of variance (ANOVA). *p* ˂ 0.05 was considered statistically significant. * Indicates significant differences with respect to the control group. # Indicates significant differences with respect to the indomethacin group.

The analysis revealed that the control group had a SOD enzyme activity of 49.91 U/mg protein, an MDA level of 15 μM, and a GSH level of 0.697 μmol/mg protein. In contrast, in the indomethacin group, SOD enzyme activity was reduced at all time points assessed, specifically at the 12 h time point (Figure 8A), representing the maximum degree of injury observed in the macroscopic analysis. This reduction in activity was related to the increase in the MDA concentration (Figure 8E), indicating increased cell damage. In the GSH analysis, a pattern similar to that of SOD was observed, with a marked reduction in GSH levels (Figure 8C).

In contrast, compared with indomethacin-treated group mice, MeEEP-treated mice presented increased SOD enzyme activity and GSH levels and decreased MDA levels at all times tested. Most notably, treatment with MeEEP increased SOD enzyme activity and restored the GSH and MDA levels to levels similar to those of the control group within 48 h (Figure 8B,D,F). These important results indicate that the treatment reduces oxidative stress, facilitating recovery from gastric injury.

### 3.8. Ethanolic Extract of Mexicali Propolis Reduces Apoptotic Death in Injured Gastric Mucosa

**Figure 9:**
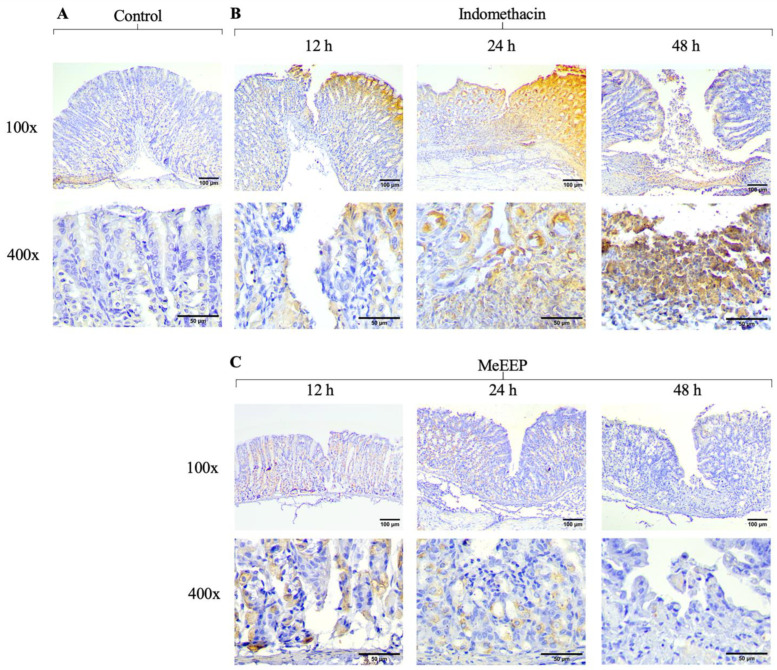
MeEEP (300 mg/kg) reduces the expression of a proapoptotic marker (Bax) in the gastric mucosa. (**A**) Photomicrographs of Bax expression in the control group. (**B**) Photomicrographs of Bax expression in the indomethacin group at 12, 24, and 48 h. Bax expression increased at all time points. (**C**) Photomicrographs of Bax expression in the MeEEP—treated group at 12, 24, and 48 h. A reduction in the intensity of the proapoptotic marker can be observed over time.

**Figure 10:**
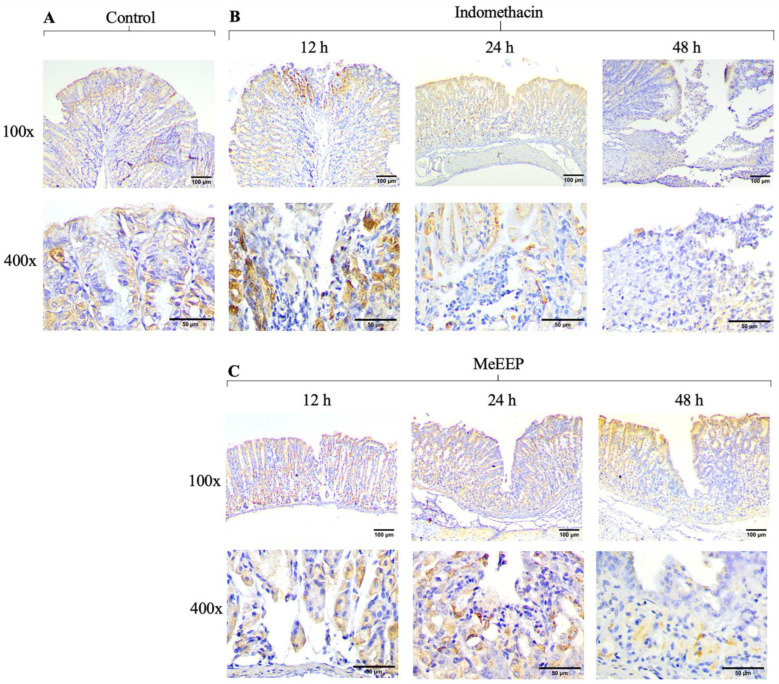
MeEEP (300 mg/kg) increased the expression of the antiapoptotic marker Bcl-2 in the gastric mucosa. (**A**) Photomicrograph of Bcl-2 expression in control group. (**B**) Photomicrographs of Bcl-2 expression in the indomethacin group at 12, 24, and 48 h. Its expression decreases as the degree of injury increases. (**C**) Photomicrographs of Bcl-2 expression in the MeEEP (300 mg/kg)-treated group.

One of the cellular mechanisms that intensifies mucosal damage is increased apoptosis. Specifically, NSAIDs such as indomethacin are characterized by mucosal damage by increasing the expression of reactive oxygen species, and in response to oxidative stress, they induce cell death. As a result, the control group expressed both Bax and Bcl-2 (Figure 9A and Figure 10A). In contrast, in the indomethacin group, overexpression of the proapoptotic marker was observed at all time points evaluated, indicating the severity of the damage caused by the drug (Figure 9B). On the other hand, Bcl-2 expression appears to be reduced; thus, it is unable to contain the damage generated in the mucosa (Figure 10B).

For the MeEEP group (300 mg/kg), different expression levels of the markers were observed. The Bax marker was initially expressed at 12 h, but its expression was reduced at subsequent time points, with a slight signal at 48 h (Figure 9C). The opposite trend was observed for the Blc-2 marker, which was expressed at higher levels than those observed in the indomethacin group at all time points (Figure 10C). Thus, by reducing Bax expression and maintaining Bcl-2 expression, MeEEP may mitigate the damaging changes generated by indomethacin.

**Figure 11:**
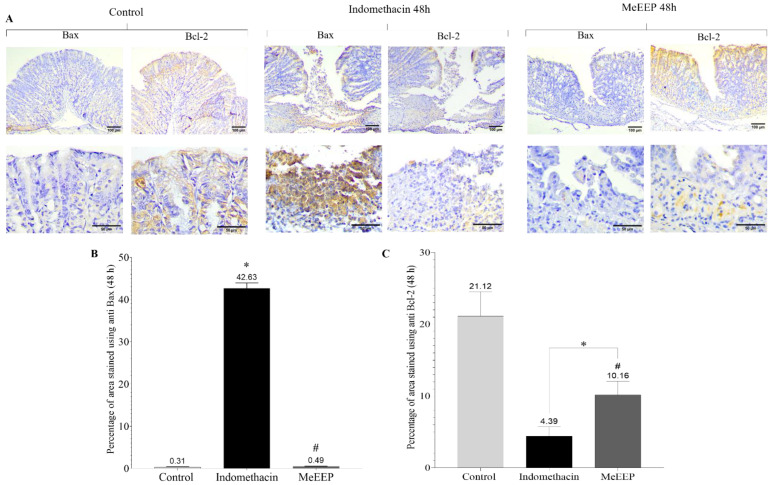
Relationship between the expression of proapoptotic and antiapoptotic factors in the gastric mucosa at 48 h after injury. (**A**) Photomicrographs of Bax and Bcl-2 expression in the control, indomethacin and MeEEP (300 mg/kg) groups. (**B**) Percentage of stained area using anti-Bax antibody. (**C**) Percentage of stained area using anti-Bcl-2 antibody. Values are shown as means ± SDs. Comparisons between groups were determined using one-way analysis of variance (ANOVA). *p* ˂ 0.05 was considered statistically significant. * Significant differences with respect to the control group. # Significant differences with respect to the indomethacin group.

To observe the relationships of both markers between the indomethacin group and the MeEEP group (300 mg/kg), a direct comparison was performed at 48 h, which revealed the outcome of the lesion in the microphotographs as well as in the statistical analysis (Figure 11A). The percentage of stained are for each marker was plotted, which provides a better view of the expression. As a result, 42.63% of the samples in the indomethacin group was stained with anti-Bax, whereas 0.49% of the samples in the MeEEP group was stained with anti-Bax (Figure 11B). In contrast, in terms of anti-Bcl-2 expression, the percentage in the indomethacin group was 4.39%, whereas that in the MeEEP group was 10.16% (Figure 11C), indicating that there was a treatment-induced increase in the expression of antiapoptotic markers.

## 4. Discussion

Propolis is a mixture of plant resins and beeswax that has attracted the interest of the scientific community due to its biological properties [97]. However, its resinous nature makes chemical characterization difficult when a single extraction solvent is used, as it comprises hydrophobic and hydrophilic substances. Thus, its chemical profile will depend on the polarity of the solvent used, as will its biomedical properties [98,99]. Consequently, it is important to characterize and analyse propolis from different regions via different extraction solvents, as each has unique properties that may be of great biomedical interest. In the present study, the chemical composition of Mexicali propolis was determined by obtaining polarity-directed extracts. Then, the extract with the greatest biomedical potential was identified on the basis of its antioxidant capacity, and its potential effect on gastric repair in the indomethacin-induced injury model was evaluated.

Three different extracts were obtained through polarity-directed extraction: an ethanolic extract, a hexane extract, and an ethyl acetate extract. To detect the compounds, fractionation of the ethanolic extract was carried out using ethyl acetate. Subsequently, this fraction was separated on a preparative TLC plate, yielding 10 distinct fractions which, under ultraviolet light, revealed the presence of compounds with conjugated structures capable of absorbing high—energy UV light (appearing as dark spots at 254 nm) and exhibiting fluorescent properties (365 nm), a characteristic behaviour of certain flavonoids and phenolic acids. These fractions were also analysed via HPLC—TOF—MS. In this analysis, seven flavonoids were identified, including naringenin, genistein, apigenin, kaempferol, chrysin, pinocembrin, and acacetin. These compounds were previously identified in Mexican propolis from semiarid areas such as Sonora, Chihuahua, Durango, Zacatecas, and Guanajuato [100,101,102,103], and pinocembrin stands out as one of the most common constituents [102].

Derivatization is a technique that improves the separation, detection, and stability of a compound, making polar or nonvolatile compounds suitable for GC—MS analysis [104]. Following chemical determination, the three extracts and the fractions (MeEEP, MeHEP, and MeEAEP) were subjected to derivatization and then analysed via GC—MS, which identified 45 compounds. This diversity in chemical composition is due to the polarity of each extract. Thus, in MeEEP and EAF, monosaccharides, alcohols, and flavonoids were identified. In MeEAEP, carboxylic acids, fatty acids, alcohols, phenylpropanoids, and alkanes were identified. In MeHEP, monoterpenes and sesquiterpenes predominated. Wesgowiec et al. performed chemical characterization of Polish propolis via direct extraction with ethanol and hexane to obtain extracts, identifying groups of compounds such as those reported in the present study [105]. On the other hand, terpenes such as α-pinene, β-caryophyllene, α-copaene, germacrene D, α-murolene, γ-cadinene, δ-cadinene, and valencene have been identified as essential oils from Brazilian propolis, which coincides with the compounds identified in the hexane extract in our study [106,107,108,109]. Therefore, this study reports for the first time the chemical composition of propolis from Mexicali, in which 52 compounds were identified.

As described above, propolis has several biomedical activities, among which its antioxidant capacity plays a very important role in protection against and recovery from diseases [110]. Antioxidant capacity comprises a set of mechanisms that protect cells and tissues from free radicals. In the case of propolis, this capacity is related to the content of phenolic compounds and flavonoid; these compounds are capable of donating hydrogen ions to free radicals to stabilize them, which prevents oxidation chain reactions [111]. When the total phenolic content, and flavone/flavonol content of the three extracts were quantified, MeEEP was found to have the highest content, represented by 36.8% TPC and 2.58% flavone/flavonol content. Both parameters are influenced by the polarity of the extraction solvent; thus, the contents of these compounds decreased according to the polarity of the extract. Some authors have reported that ethanol is the most widely used solvent for the extraction of bioactive compounds because it results in high extraction efficiency for polyphenols, and some authors also emphasize that 70% ethanol is the most suitable concentration [112,113], which was used in the present study.

Phenolic compounds are considered the main bioactive constituents of propolis; depending on the extraction method and botanical origin, they can represent 40–50% of the sample [114]. On the other hand, in some studies of propolis from Brazil, the phenolic content ranged from 6.4 to 15.2% [115], whereas for propolis from Poland, a total of 21.9% is reported [116]. Moreover, several investigations performed on propolis from regions of Mexico reported that the total phenolic content was 139, 109, 126 and 167 to 246 mg GAE/g for the regions of Durango, Zacatecas, Chihuahua, and Guanajuato, respectively, and the flavonoid content was 90, 70, 71, and 58.34 to 87.5 mg QE/g, respectively, for the same areas [102,103]. These data are consistent with the results obtained for propolis from Mexicali. Additionally, NOM-003-SAG/GAN-2017 described the minimum requirements for Mexican propolis, highlighting the phenolic and flavone/flavonol content [22]. Therefore, compared with the results of this work, MeEEP complies with the technical standard.

The DPPH reduction method was used to determine the antioxidant capacity of the three Mexican propolis extracts, and the EC_50_ and percentage inhibition of DDPH at 1 mg/mL were evaluated. Both assessments revealed that, of the three extracts, the ethanolic extract had the highest EC_50_ of 112.16 μg/mL and the highest percentage inhibition of 92.6%, followed by the ethyl acetate extract. In this context, the EC_50_ of several propolis samples from Mexico has been estimated. For Sonora, the EC_50_ ranges from 58.8 to 98.7 μg/mL, and the authors reported that this variation is related to the seasonality of the propolis [117]. Guanajuato presented an EC_50_ of 67.9 μL/mL [103], Durango, Zacatecas, and Chihuahua presented values between 975 and 1145 TE/g of extract [102], and propolis from Chihuahua presented an EC_50_ of 15.75 μg/mL [101]. Thus, the values obtained from MeEEP are within the values reported in these studies. In contrast, in the hexane extract, the EC_50_ was not determined, and it had a minimum inhibition percentage of 1.4%. Therefore, it was assumed that it did not present any activity, which coincides with other authors who reported that the nonpolar extracts of propolis from Poland and Canada extracted with hexane did not present antioxidant capacity [105,110].

Our data on antioxidant capacity and the determination of phenolic compounds, flavones, and flavonols revealed that the ethanolic extract of Mexicali propolis had the highest biomedical potential; therefore, its effect on gastric repair was evaluated in an indomethacin-induced injury model.

In line with this objective, low toxicity is an important factor in safely evaluating a natural product. Propolis has been reported to have low oral toxicity; specifically, for MeEEP, an LD_50_ above 2000 mg/kg has been estimated, similar to that reported for other propolis [118]. However, the complexity and particularity of each propolis makes this dose change. This is the case for propolis from Chihuahua, a region similar to Mexicali, which has an LD_50_ above 2000 mg/kg [20,101], whereas a long-term oral toxicity study reported that daily administration of 1000 mg/kg Brazilian propolis for 90 days did not generate histopathological changes in mice [119]. These reports corroborate the low toxicity of propolis.

Given that MeEEP has an LD_50_ greater than 200 mg/kg, a lower dose is safe for evaluating its effect on a gastric repair model, and a dose of 300 mg/kg was established, taking into consideration the doses of other propolis used in gastroprotection reports [20,94].

NSAIDs, including indomethacin, are widely used drugs worldwide and are major causes of gastrointestinal injury, exerting their ulcerogenic effect through the inhibition of prostaglandin synthesis and through topical mechanisms. The latter involves increased oxidative stress, increased cell membrane permeability, and the induction of cell death by necrosis and apoptosis [120].

The macroscopic results obtained in this study reveal the time course of indomethacin-induced gastric injury. This injury is established at 6 h after indomethacin administration. The maximum value of the percentage of bleeding area is reached at 12 h. Then, the value subsequently decreases, and the recovery phase begins, as represented by a decrease in signs of injury such as haemorrhage, erosion, oedema, and mucosa erythema. This behaviour in the injury and recovery phase of the gastric mucosa has been observed in other models of acute hydrochloric acid/ethanol and indomethacin-induced gastric mucosal injury. In the HCl/ethanol induction model, the injury phase lasts approximately 6 h, and the recovery phase starts between 12 and 18 h, as represented by the ulceration index [121]. Likewise, in the indomethacin model, erosions are established at 5 h, with peak progression noted between 24 and 48 h [122]. However, in the present study, the administration of MeEEP reduced the percentage of the bleeding area at 12 h, dramatically decreasing the peak injury from 32.1% without treatment to 2.65% upon treatment with the extract. These findings suggest the potential of MeEEP in the repair of gastric injury.

The efficacy of plant—derived natural products in the repair of the gastric mucosa has been described, and their effects are related to the biomedical activities they usually exert simultaneously. However, when evaluating each extract, the type of extract and the duration of treatment should be considered [123,124]. In the case of propolis, some research has reported that when evaluating the effects of the hydroalcoholic extract of Brazilian propolis at a dose of 300 mg/kg in a model of chronic injury induced by acetic acid, the extract accelerated the healing process by 71%, conferring activity to artepillin C [9].

Similarly, when evaluating the effects of the acetone extract of Cameroon propolis, it has been reported that at doses of 200, 400, and 600 mg/kg, it reduces the index of ulceration in models of ulceration induced by acetic acid and ethanol/indomethacin. When the chemical composition was identified, it was found to be composed mainly of chrysin, caffeic acid, and p-hydroxybenzoic acid [8], with chrysin representing a compound that is also present in MeEEP. The effects of both types of propolis are attributed to the restoration of the oxidative balance. On the other hand, the effects of chrysin on the healing of acetic acid-induced gastric ulcers were evaluated individually. At a dose of 10 mg/kg, chrysin reduced the macroscopic lesion area by 46.1% after 7 days of treatment, a period during which injury and rapid healing phases were established [34]. By comparing the results of the studies described above with those of MeEEP, we propose chrysin as one of the compounds that contributes to mucosal repair.

An important element of the evaluation of the effect of a treatment on gastric repair is histological analysis; however, most of the studies reported in the literature are based mainly on macroscopic analysis, which may limit information on the treatment effect [124]. Histological analysis allows us to observe the characteristics of the lesions, such as the severity of the damage, the signs of injury, and the type of cellular damage present in the tissue. These elements allow us to make a detailed assessment of the effect of a treatment.

In this work, histological analysis revealed that indomethacin induces a series of mucosal changes, represented by the presence of inflammatory infiltrate, haemorrhage, necrosis, apoptosis, and erosion of the mucosal surface; these changes are associated with signs that worsen with time, beginning with the establishment of the lesion at 6 h and mucosal rupture at 12 h. After this time, the recovery phase begins (24 h). However, at 48 h, areas of recurrence can be observed, an important characteristic that hinders proper recovery of the lesion [17], resulting in an area consistent with the course of the indomethacin-induced lesions in this study [125]. Nevertheless, this course of the lesion is altered by the administration of MeEEP, which decreases the severity of the lesion and prevents recurrence in a short time by protecting the mucosa. Previous studies have shown similar results, where administration of the acetonic extract of Cameroon propolis led to histological normalization of the gastric mucosa [8], and the hydroalcoholic extract of Brazilian propolis promoted recovery of the lesion [9].

Inflammation is a process involving gastric tissue each time it is injured or repaired; however, excessive activity causes damage and prevents proper recovery [126]. Neutrophils are the first immune cells to arrive at the site of injury. These cells are characterized by a high MPO content and secrete proinflammatory cytokines and reactive oxygen species. Thus, MPO activity is considered a marker of inflammatory infiltration. It has been reported that indomethacin administration induces a time—dependent increase in MPO activity, reaching high values between 12 and 24 h; this increase is related to the degree of ulcerative damage at those times, and subsequently, the enzyme activity decreases [15]. These values are similar to those observed in the indomethacin group in this work.

Moreover, TNF-α, IL-1β, and IL-6 are cytokines that stimulate the production of leukocyte-attracting molecules, building a circuit that perpetuates inflammation at the site of injury, promoting tissue damage and even injury recurrence [16,126]. In this work, we observed that the administration of MeEEP reduced the levels of these cytokines and decreased the level of inflammatory infiltration, which prevented the establishment of the inflammatory cycle, attenuated damage to the gastric tissue, and allowed for the recovery of the lesion. This decrease in parameters originated after 12 h and remained until 48 h. Therefore, keeping inflammation in check is a key mechanism in repair. The modulation of inflammation is so important that the polarization of M1— to M2—type macrophages promotes the regeneration of gastric tissue by reducing the proinflammatory environment and favouring the release of anti—inflammatory cytokines and growth factors that stimulate healing [126]. Although, in this study, the polarization of macrophages was not evaluated, a reduction in the proinflammatory environment was observed.

Oxidative stress is an important factor in the damage caused by NSAID administration, triggering different mechanisms of injury, such as apoptosis and lipid peroxidation. Specifically, indomethacin administration is associated with increased hydroxyl radical, hydrogen peroxide, and superoxide anion levels, as well as glutathione depletion and increased MDA levels [12,14]. The ability of the gastric mucosa to repair itself is influenced by the regulation of the antioxidant defence system and the production of reactive oxygen species; thus, the activation of antioxidant mechanisms contributes favourably to the repair of gastric ulcers [12].

The results of this study suggest that the administration of MeEEP decreased the severity of gastric lesions by increasing SOD and GSH enzymatic activity. This increase in the antioxidant system prevented oxidative damage to the tissue, represented by low levels of MDA at each of the time points evaluated, which ended at 48 h, when the levels of MDA and GSH returned to values similar to those of the control group. These results indicate that oxidative damage was reduced, allowing for the recovery of the lesions.

Several studies have demonstrated that some natural products contribute to the recovery of gastric injury through their antioxidant activity; for example, the administration of Saudi Arabian honey resulted in recovery from injury after decreasing the levels of MDA and increasing the enzymatic activity of SOD, CAT, and GPx, improving the architecture of the gastric mucosa [27]. Similarly, the administration of anthocyanins isolated from black rice alleviated naproxen-induced gastric ulcers by reducing oxidative stress through the activation of the Nrf2/ARE signalling pathway [13]. This pathway is responsible for the induction of several antioxidant enzymes associated with cytoprotective effects. It has also been reported that some phytochemicals, such as pinocembrin [28], chrysin [32], and acacetin [45], flavonoids present in MeEEP, exert their antioxidant effect by activating this pathway.

Apoptosis is a mechanism of NSAID—induced injury caused by increased reactive oxygen species and endoplasmic reticulum (ER) stress [125]. This mechanism is regulated by proapoptotic (Bax, Bak, and caspases) and antiapoptotic (Bcl-2 and Bcl-xl) factors. Indomethacin and aspirin administration trigger increases in Bax and caspase 3 and 8 expression, as well as a reduction in Bcl-2 expression [125,127,128].

Specifically, indomethacin administration triggers a gradual time-dependent increase in apoptosis, starting after 3 h and reaching the highest level of expression between 6 and 12 h, because cytoprotective mechanisms are unable to compensate for the damage generated by the drug, triggering gastric mucosal injury [125,129]. This finding is consistent with that observed in the indomethacin group in the present study. However, Bax expression was noted for up to 48 h; thus, the mucosa was more susceptible to damage despite being in a process of recovery. However, the administration of MeEEP reduced Bax expression and increased Bcl-2 expression, protecting the gastric mucosa from damage and preventing the spread of the lesion.

This effect was observed for other natural products that promote gastric mucosal repair by negatively regulating apoptosis. Specifically, chrysin has been reported to reduce caspase-3 expression [34], whereas liquorice flavonoid reduces Bax, and caspase 3 expression, and increases Bcl-2 expression [130]. In addition, several antioxidant mechanisms have been reported to be involved in the inhibition of NSAID-induced proapoptotic signals [14,131]. Furthermore, by decreasing TNF-α levels, MeEEP could contribute to the reduction in apoptotic expression, as this cytokine regulates the extrinsic apoptotic response [11].

**Figure 12:**
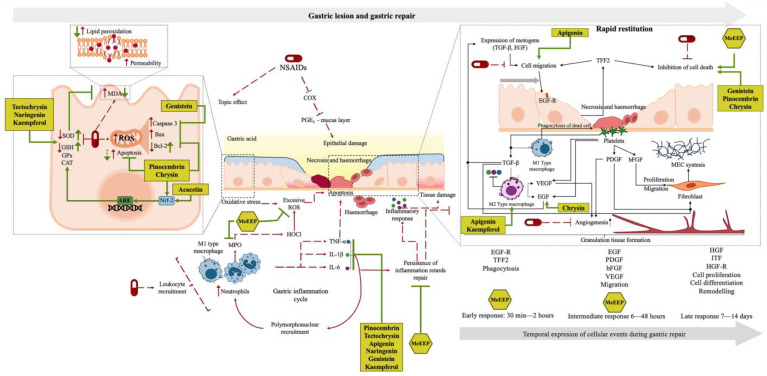
Proposed mechanism of action of MeEEP in the repair of acute gastric lesions induced by indomethacin. The mechanisms of injury and inhibition of repair processes are indicated by red arrows. The normal mucosal repair process is indicated by the black arrows. The effects of MeEEP demonstrated in this research are represented by green hexagons, illustrating that the extract ameliorates mucosal injury through a cytoprotective effect, controlling the exacerbation of inflammation, reducing oxidative stress, and regulating apoptosis during the early and intermediate response of gastric repair. On the other hand, MeEEP could control this process due to the individual or synergistic effect of pinocembrin, tectochrysin, chrysin, apigenin, naringenin, acacetin genistein, and kaempferol, compounds identified in the extract by HPLC—TOF—MS and GC—MS. The mechanisms of action of the compounds are indicated by green arrows.

The chemical composition analysis of MeEEP revealed the presence of bioactive compounds that may contribute to the protection and repair of gastric mucosa by reducing oxidative stress, decreasing inflammation, regulating apoptosis, and promoting cell migration (Table 8), one of the critical mechanisms in gastric repair. The main bioactive compounds identified in MeEEP include pinocembrin, tectochrysin, chrysin, apigenin, naringenin, acacetin, genistein, and kaempferol. Most of these compounds are flavonoids, which may act individually or synergistically through different mechanisms, reducing the severity of the lesion and promoting repair, as illustrated in Figure 12.

Firstly, anti—inflammatory activity: MeEEP may control the exacerbation of inflammation in the gastric mucosa and the tissue damage caused by leucocyte recruitment and proinflammatory cytokine secretion. In particular, pinocembrin, tectochrysin, apigenin, naringenin, genistein, and kaempferol have demonstrated anti-inflammatory effects in several models. For instance, in in vitro studies, pinocembrin has been shown to inhibit the production of TNF-α, IL-1β, and IL-6 in macrophages in a dose-dependent manner [29]. Similarly, tectochrysin inhibits the production of TNF-α and IL-1β in LPS-stimulated macrophages [31], and naringenin reduces the production of TNF-α and IL-1β in vivo in a carrageenan-induced paw oedema model [41].

Additionally, macrophage polarization and promotion of growth factors: the polarization of macrophages from M1 to M2 is a crucial mechanism in gastric repair, as M2 macrophages exhibit anti-inflammatory effects and secrete growth factors such as VEGF and EGF. This polarization occurs in response to TGF-β, which is released by platelets and M1 macrophages during the initial process of gastric injury; subsequently, TFF2 (secreted by mucous cells), EGF, and TGF-β promote cell migration to restore epithelia continuity [126,132,133]. In this context, in studies, apigenin [37] and kaempferol [55] have independently been shown to promote M2 polarization. Specifically, apigenin has been found to accelerate skin wound healing through this mechanism [37]. Furthermore, chrysin has been reported to promote EGF expression in a gastric healing model [34].

Finally, antioxidant activity and apoptosis regulation: the antioxidant activity of MeEEP may help reduce oxidative stress, a mechanism of injury that hinders gastric repair. The activation of the Nrf-2/ARE pathway, which mitigates oxidative stress through the expression of genes involved in antioxidant defence (SOD, CAT, and GPx), is essential for reducing oxidative damage [13]. Pinocembrin, acacetin, and chrysin have been described to activate this pathway, thereby protecting against apoptosis induced by oxidative stress [28,32,33,45]. Additionally, pinocembrin [28], genistein [52], and chrysin [33] regulate Bax and Bcl-2 levels, decreasing the proapoptotic effect caused by NSAIDs and supporting gastric mucosa repair.

Although the results reported in these studies do not directly relate to gastric lesion models, they provide a valuable framework for understanding the effects of the compounds present in MeEEP, which may contribute to our study’s gastric lesion repair.

The findings of this study demonstrate that MeEEP contributes to gastric mucosal lesion repair during the initial stages of the healing process, which occur within the first 48 h (early and intermediate phases), providing an initial insight into this effect. The results suggest that MeEEP is a promising candidate for evaluating its reparative effect in chronic models that involve processes such as cell proliferation and gastric gland remodelling, associated with later stages of repair. This study paves the way for further research to evaluate the effects of MeEEP on virulence factors associated with *H. pylori*—induced gastric ulcers or to investigate in more depth the mechanisms of action of the compounds that constitute propolis. Additionally, its potential implications for human gastric health should be considered, as propolis is a natural product widely used in traditional medicine.

## 5. Conclusions

The present study revealed that propolis from Mexicali has great chemical diversity and is rich in compounds such as flavonoids, monosaccharides, terpenes, alcohols, and fatty acids. Of the three extracts, the ethanolic extract exhibited the highest antioxidant capacity, as well as the highest total phenolic content, and flavone/flavonol content. In addition, it exhibited low acute oral toxicity.

After evaluating the effects of MeEEP on gastric repair, revealed that MeEEP contributes to recovery from acute gastric injury through a cytoprotective mechanism by promoting the expression of the antiapoptotic marker Bcl-2, increasing the glutathione concentration and SOD enzyme activity, and decreasing inflammation in gastric tissue. The mechanisms attributed to the chemical composition of the extract include the presence of naringenin, genistein, apigenin, kaempferol, chrysin, pinocembrin, and acacetin. MeEEP is a good candidate for evaluating its gastric repair effect in chronic models and investigating the mechanisms of action involved in recovery from injury.

## Figures and Tables

**Table 1:** Organoleptic characteristics of Mexicali propolis.

Parameter	Characteristic
Colour	Green-brown
Odour	Resinous
Flavour	Balsamic
Consistency	Rigid at room temperature

**Table 2:** Yields of the extracts and fractions.

Extract/Fraction	Yield%
MeEEP	28.4
MeEAEP	8.05
MeHEP	27.05
EAP	82.72
F1	3.25
F2	4.17
F3	4.58
F4	3.46
F5	7.02
F6	13.95
F7	10.18
F8	8.9
F9	10.08
F10	34.31

**Table 3:** Compounds present in MeEEP identified by HPLC—TOF—MS.

No	Compound	Type of Compound	RT (min)	Molecular Ion (m/z) [M-H]―	Relative Error (ppm)	Fraction/Extract
1	Naringenin	Flavanone	15.954	271.0732	10.35	10, MeEEP, EAP
2	Genistein	Isoflavone	17.58	269.0585	11.25	9, MeEEP, EAP
3	Apigenin	Flavone	17.457	269.0585	−0.96	8
4	Kaempferol	Flavonol	18.161	285.0528	−9.96	MeEEP
5	Chrysin	Flavone	28.61	253.0636	−2.98	3, 8, 9, 10, MeEEP, EAP
6	Pinocembrin	Flavanone	28.944	255.0794	−2.46	10, MeEEP, EAP
7	Acacetin	Flavone	30.403	285.0752	6.54	9

**Table 4:** Compounds present in MeEEP identified using GC—MS (TMS—Derivative).

No	Compound	Type of Compound	TR	Similarity (%)	Area (%)
1	Xylonic acid	Monosaccharide	15.830	91	0.10
2	*α*-D-Glucopyranose	Monosaccharide	20.642	94	2.03
3	*β*-D-Glucopyranose	Monosaccharide	21.258	91	3.44
4	2- Nonadecanone	Hydrocarbon	24.029	95	0.26
5	Inositol	Alcohol	24.395	90	0.95
6	Pinocembrin	Flavanone	30.598	90	0.44

**Table 5:** Compounds present in EAF identified using GC—MS (TMS—Derivative).

No	Compound	Type of Compound	TR	Similarity (%)	Area (%)
1	β-Galactopyranose	Monosaccharide	22.425	91	0.33
2	2-Nonadecanone	Hydrocarbon	24.036	89	0.20
3	Pinostrobin chalcone	Chalcone	29.238	94	0.36
4	Pinocembrin	Flavanone	30.598	97	1.07
5	Tectochrysin	Flavone	32.266	86	0.39

**Table 6:** Compounds present in MeEAEP identified using GC—MS (TMS —derivative).

No	Compound	Type of Compound	TR	Similarity (%)	Area (%)
1	Benzyl alcohol	Alcohol	4.937	95	0.02
2	Benzoic acid	Aromatic carboxylic acid	6.503	95	0.03
3	Glycerol	Alcohol	7.061	91	0.44
4	Cinnamic alcohol	Alcohol	10.179	95	0.06
5	Capric acid	Carboxylic acid	10.782	97	0.05
6	4- Hydroxybenzyl alcohol	Alcohol	11.924	98	0.05
7	Apiol	Phenylpropanoid	15.805	91	0.05
8	Thymol	Terpenoid	17.139	83	0.15
9	Myristic acid	Unsaturated fatty acid	19.154	99	0.33
10	α-D-Xylopyranose	Monosaccharide	19.808	83	0.08
11	Palmitic acid, ethyl ester	Fatty ester	21.964	98	0.25
12	Palmitic acid	Fatty acid	22.990	99	0.84
13	Heneicosane	Alkane	23.882	97	0.52
14	Oleic acid	Fatty acid	26.185	99	0.39
15	Stearic acid	Fatty acid	26.518	99	0.93
16	Octacosane	Alkane	27.397	95	0.97
17	Hexacosane	Alkane	32.145	96	0.62

**Table 7:** Compounds present in MeHEP identified using GC—MS (TMS—Derivative).

No	Compound	Type of Compound	TR	Similarity (%)	Area (%)
1	α-Pinene	Monoterpene	2.692	95	0.03
2	Camphor	Monoterpene	4.989	94	0.06
3	Myrtenal	Monoterpene	5.771	95	0.02
4	D-Verbenone	Monoterpene	6.009	91	0.05
5	Cuminaldehyde	Monoterpene	6.515	96	0.02
6	Bornyl acetate	Monoterpene	7.324	99	0.07
7	α-Copaene	Sesquiterpene	9.203	95	0.03
8	β-Caryophyllene	Sesquiterpene	10.185	99	0.26
9	α-Humulene (α-Caryophyllene)	Sesquiterpene	10.916	95	0.08
10	Germacrene D	Sesquiterpene	11.507	97	1.51
11	Guaiazulene	Sesquiterpene	11.673	81	0.07
12	α-Muurolene	Sesquiterpene	11.853	95	0.16
13	γ-Cadinene	Sesquiterpene	12.193	95	0.12
14	δ-Cadinene	Sesquiterpene	12.373	92	0.19
15	Caryophyllene oxide	Sesquiterpene	13.803	90	0.75
16	β-Guaiene	Sesquiterpene	16.151	93	0.22
17	Valencene	Sesquiterpene	19.365	91	0.18
18	Palmitic acid, ethyl ester	Fatty ester	21.970	93	0.43
19	Ent-Kaurene	Diterpene	23.080	96	0.40
20	Tricosane	Alkane	27.404	95	1.01

**Table 8:** Biomedical activities reported for the compounds identified in Mexicali propolis.

Compound	Antioxidant	Anti-Inflammatory	Gastroprotective	Healing	Cell Migration	Antiapoptotic	Ulcer-Healing
Pinocembrin	[28]	[29]				[28]	
Tectochrysin	[30]	[31]					
Chrysin	[32,33]					[33]	[34]
Apigenin	[35]	[36]		[37]	[37]		
Naringenin	[38,39,40]	[41]	[42,43]	[44]			
Acacetin	[45]	[46]					
Genistein	[47]	[48,49]	[50]	[51]		[52]	
Kaempferol	[53]	[54,55]		[56]			
Capricacid	[57]	[57,58]					
Myristicacid		[59]					
Palmiticacid		[59]					
Stearicacid	[60]						
Oleicacid	[61]	[61]			[62]		
Apiol	[63]						
Inositol	[64]	[64]					
4-hydroxybenzylalcohol	[65]	[66]					
Bornylacetate		[67]					
Thymol	[68,69]	[70]			[71]		
α-pinene	[72]	[73]	[74]				
Camphor	[75]	[76,77]		[78]			
Myrtenal	[79]	[79]					
β-Caryophyllene	[80,81]	[82]		[83]	[83]		
α-Humulene		[84,85]	[86]				
Guaiazulene	[87]						
Caryophylleneoxide		[88,89]					
Valencene	[90]	[90]					
Benzylalcohol				[91]			
Octacosane					[92]		
Eicosane					[92]		

**Table 9:** Antioxidant capacity of Mexicali propolis extracts.

Extract	EC_50_	TPC	Flavone/Flavonol Content
MeEEP	112.16 μg/mL	368 mg GAE/g—36.8%	25.87 mg QE/mg—2.58%
MeEAEP	1180 μg/mL	73 mg GAE/g—7.3%	7.5 mg QE/mg—0.75%
MeHEP	-	-	-

## Data Availability

The data used to support the findings of this study are available from the corresponding author upon request.

## Supplementary Materials

The following supporting information can be downloaded at https://www.mdpi.com/article/10.3390/antiox14010065/s1.
