# Supplementary material for: Chemical Composition of Mexicali Propolis and Its Effect on Gastric Repair in an Indomethacin-Induced Gastric Injury Murine Model
Source: Antioxidants (Basel). 2025 Jan 8;14(1):65. doi: 10.3390/antiox14010065 (PMC11762497; doi:10.3390/antiox14010065)
Supplement: Supplementary file 1 [file antioxidants-14-00065-s001.zip › antioxidants-3241068-supplementary.pdf]

Supplementary material

Chemical Composition of Mexicali Propolis and Its Effect on Gastric Repair in an Indomethacin-Induced Gastric Injury Murine Model

Mass spectra of the compounds identified in the Mexicali ethanolic extract of propolis by HPLC-TOF-MS analysis.

Figure S1. HPLC-TOF-MS chromatograms of the MeEEP, and the fractions obtained from the EAF by Prep-TLC. After each chromatogram, the mass spectrum of the identified compound and its standard are listed.

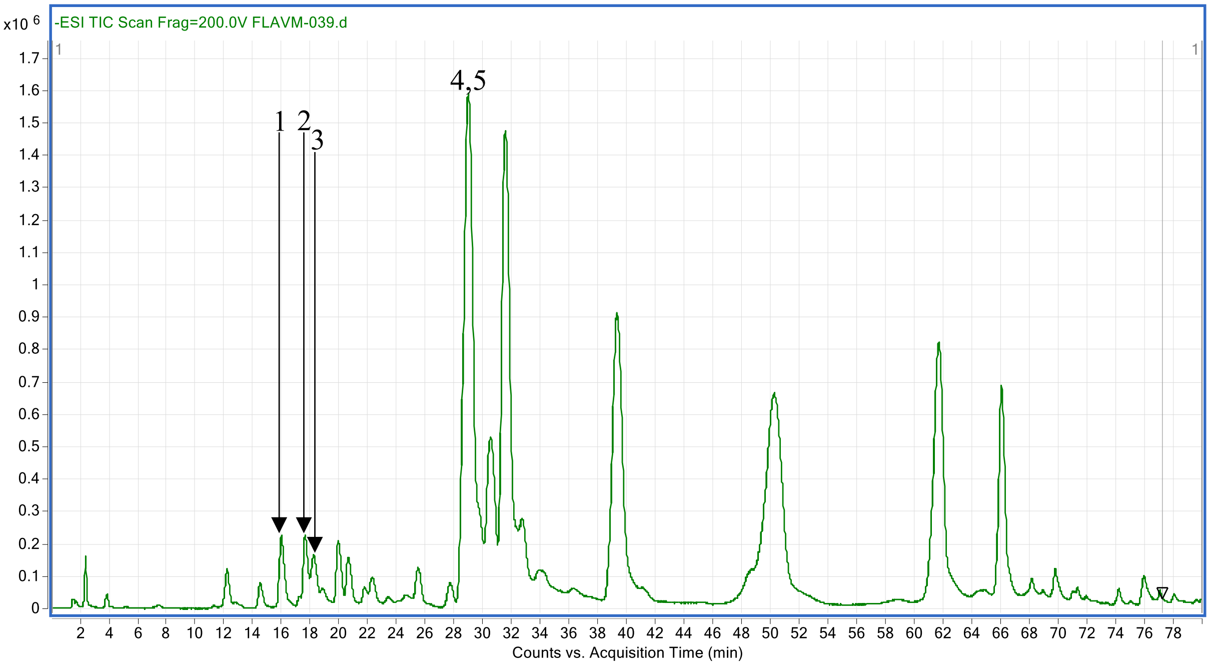

A.- HPLC-TOF-MS chromatogram of MeEEP (naringenin, genistein, kaempferol, chrysin, and pinocembrin).

1.- Naringenin

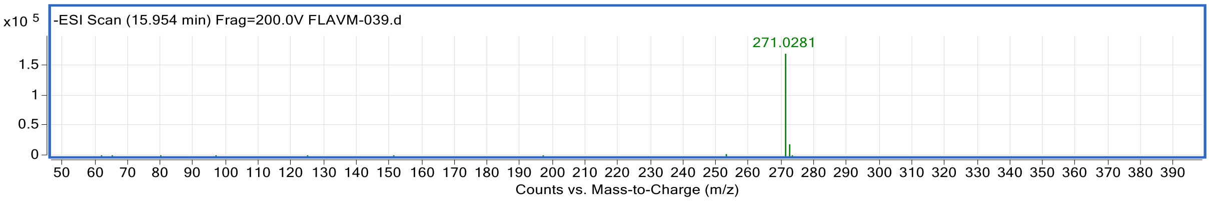

Standard

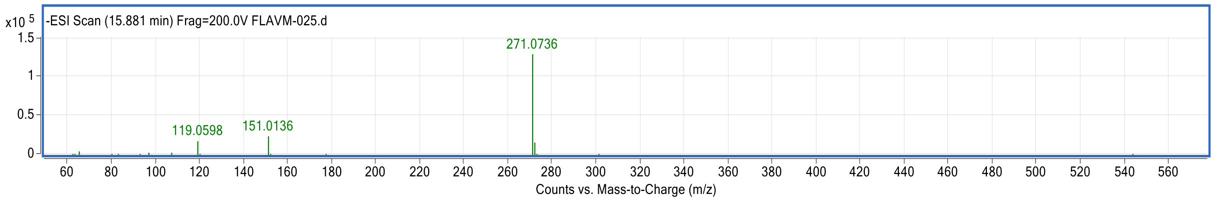

2.-Genistein

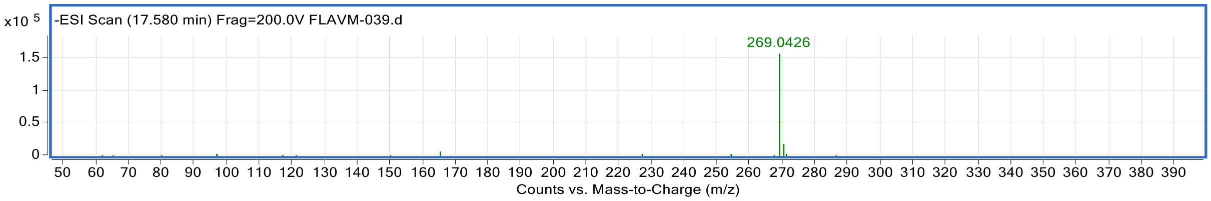

Standard

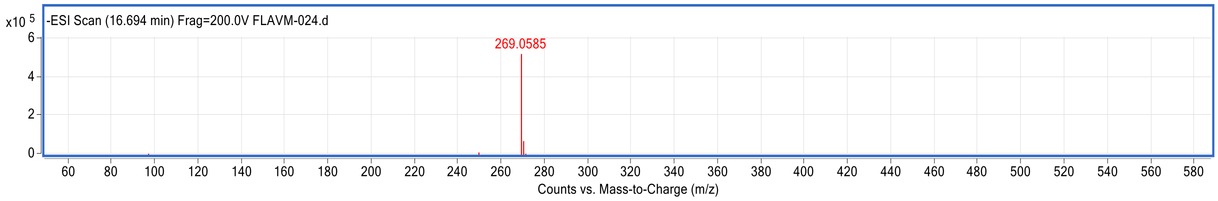

3.- Kaempferol

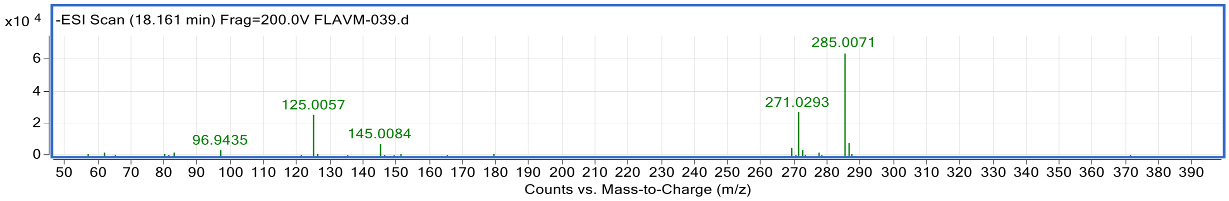

Standard

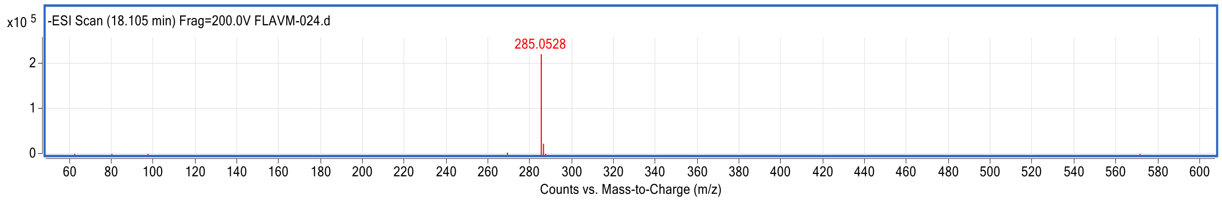

4.- Chrysin

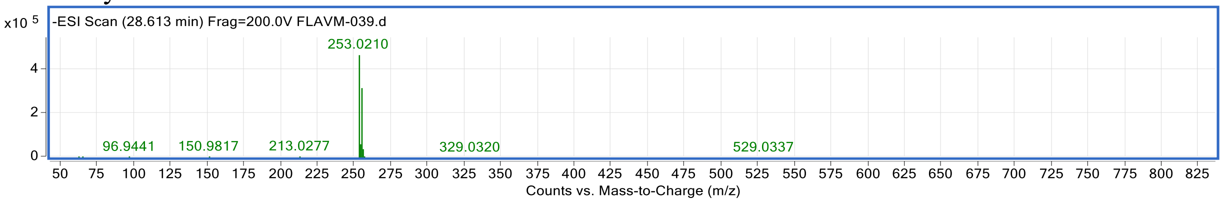

Standard

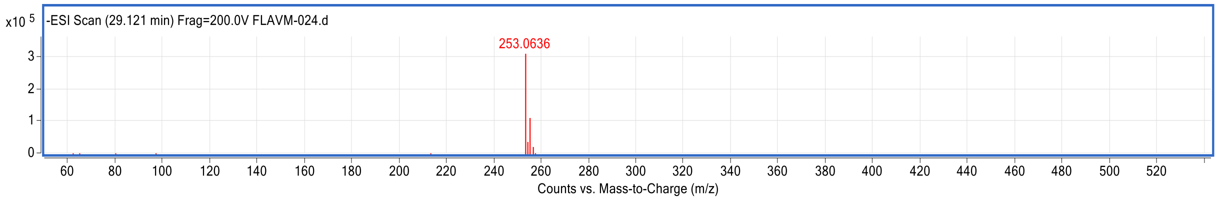

5.-Pinocembrin

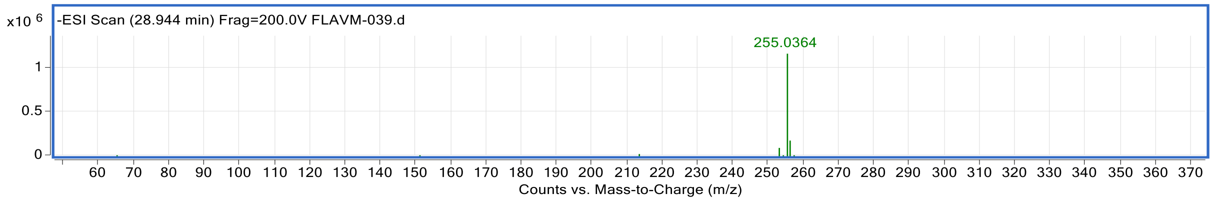

Standard

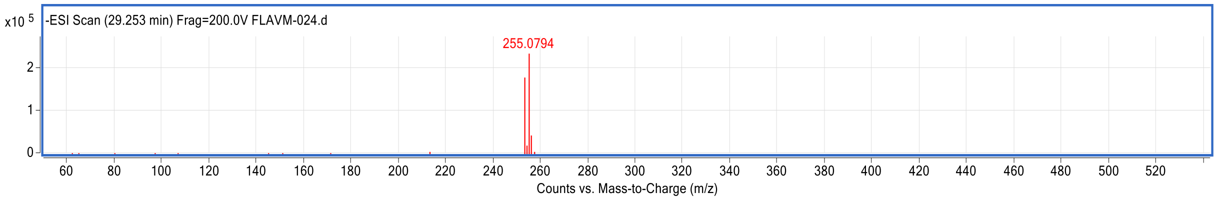

Fractions of Ethyl acetate fraction of Mexicali ethanolic extract of propolis

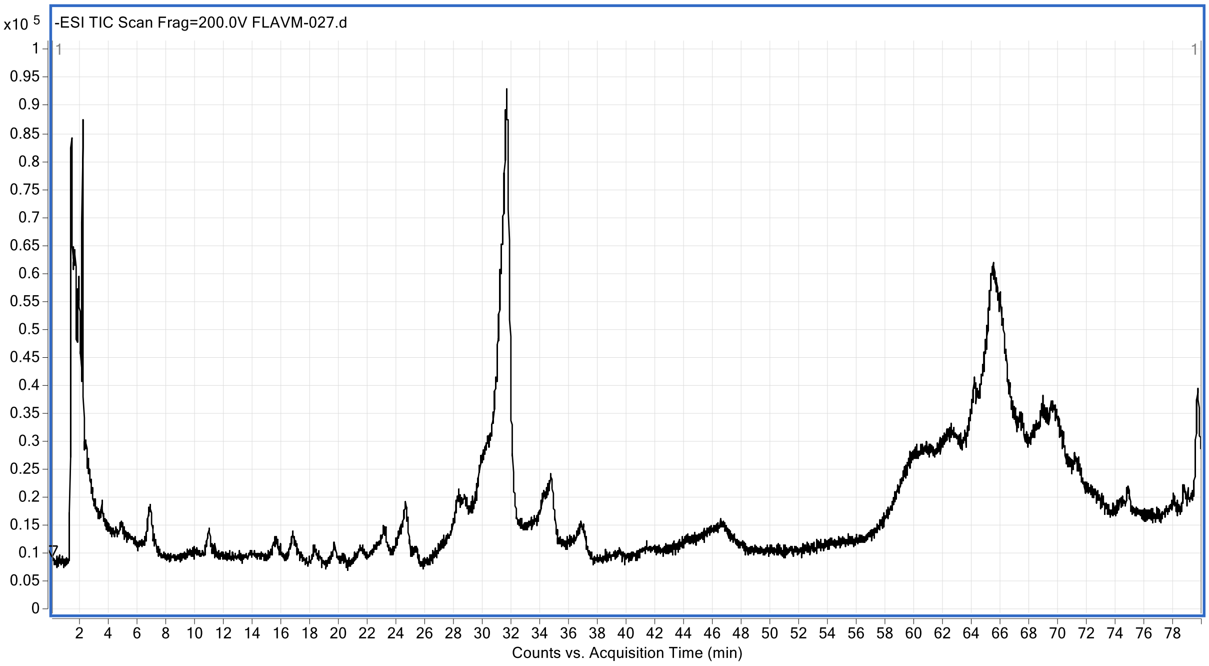

B.- HPLC-TOF-MS chromatogram of fraction one (unidentified compounds).

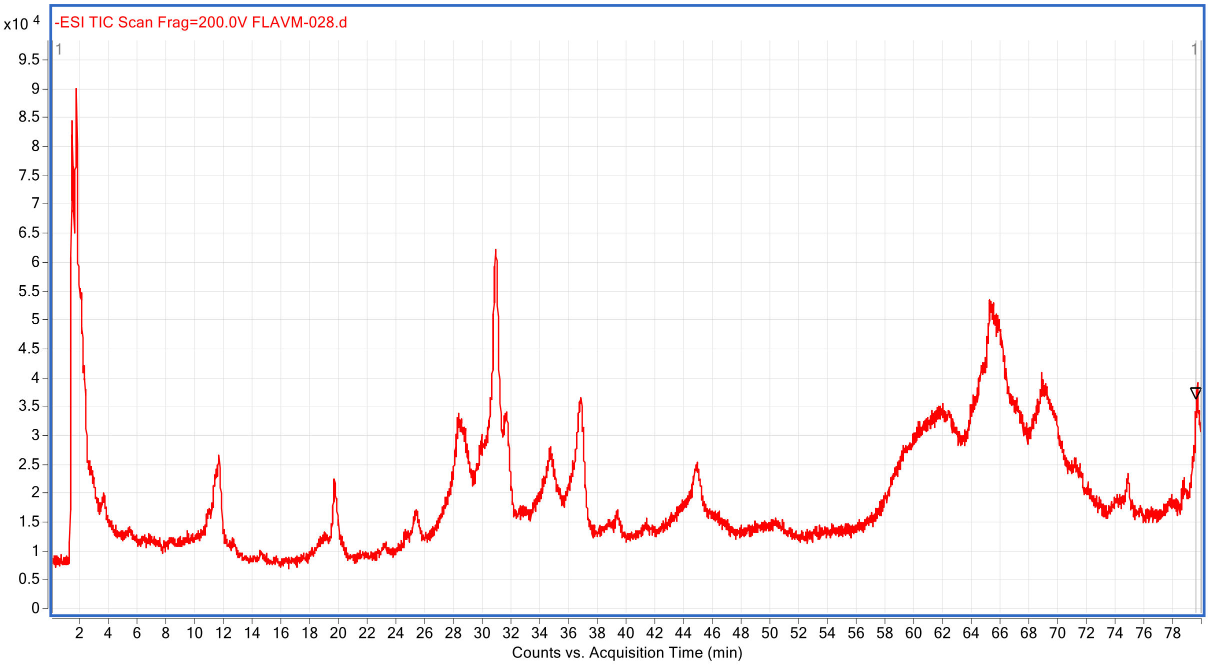

C.- HPLC-TOF-MS chromatogram of fraction two (unidentified compounds).

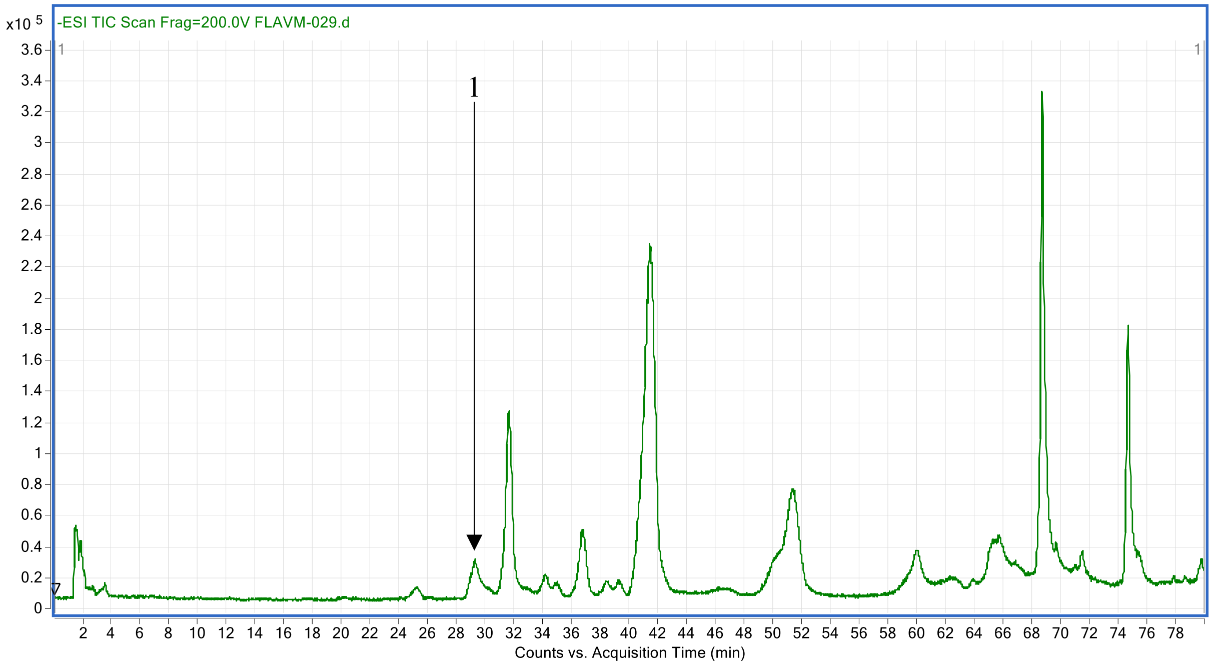

D.- HPLC-TOF-MS chromatogram of fraction three (chrysin).

1.- Chrysin

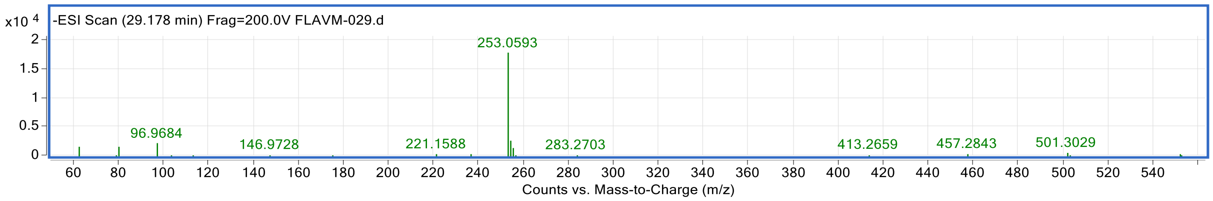

Standard

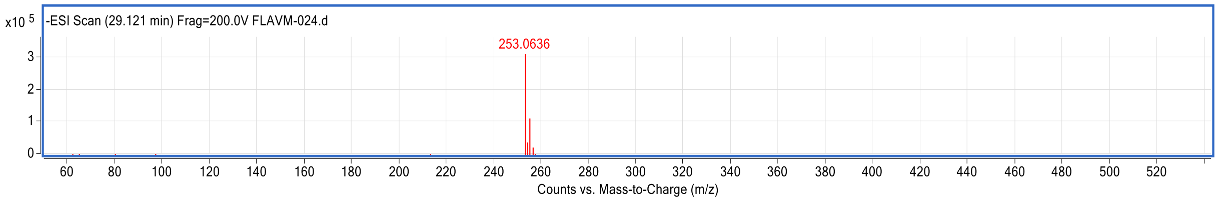

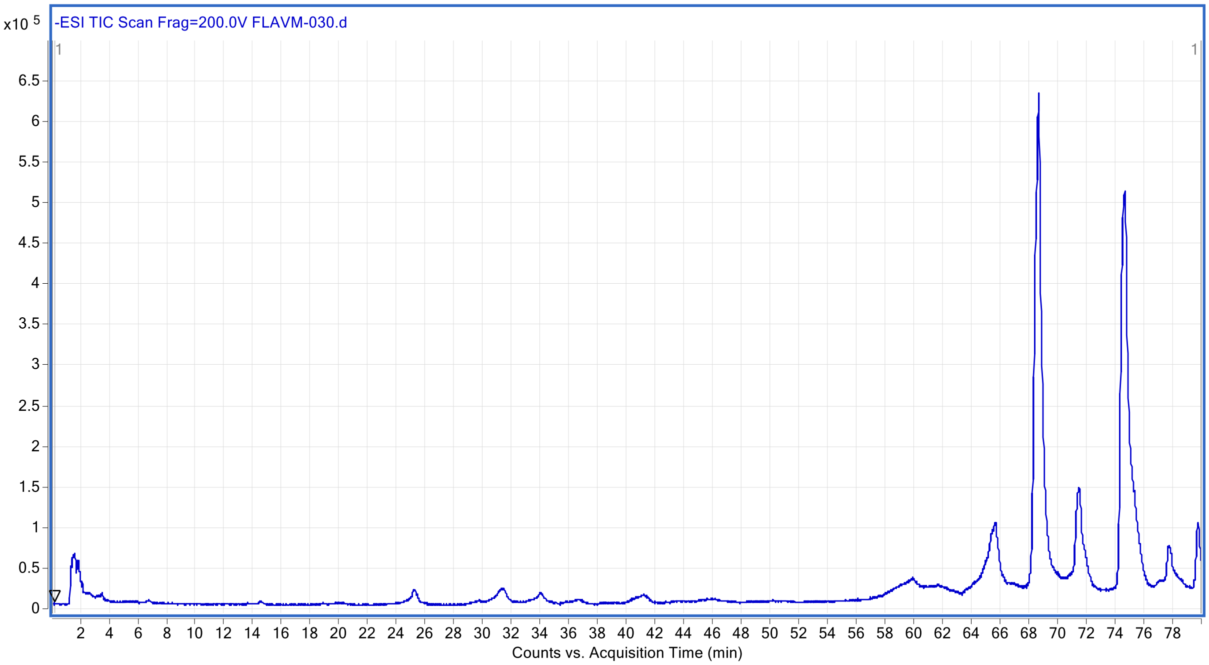

E.- HPLC-TOF-MS chromatogram of fraction four (unidentified compounds).

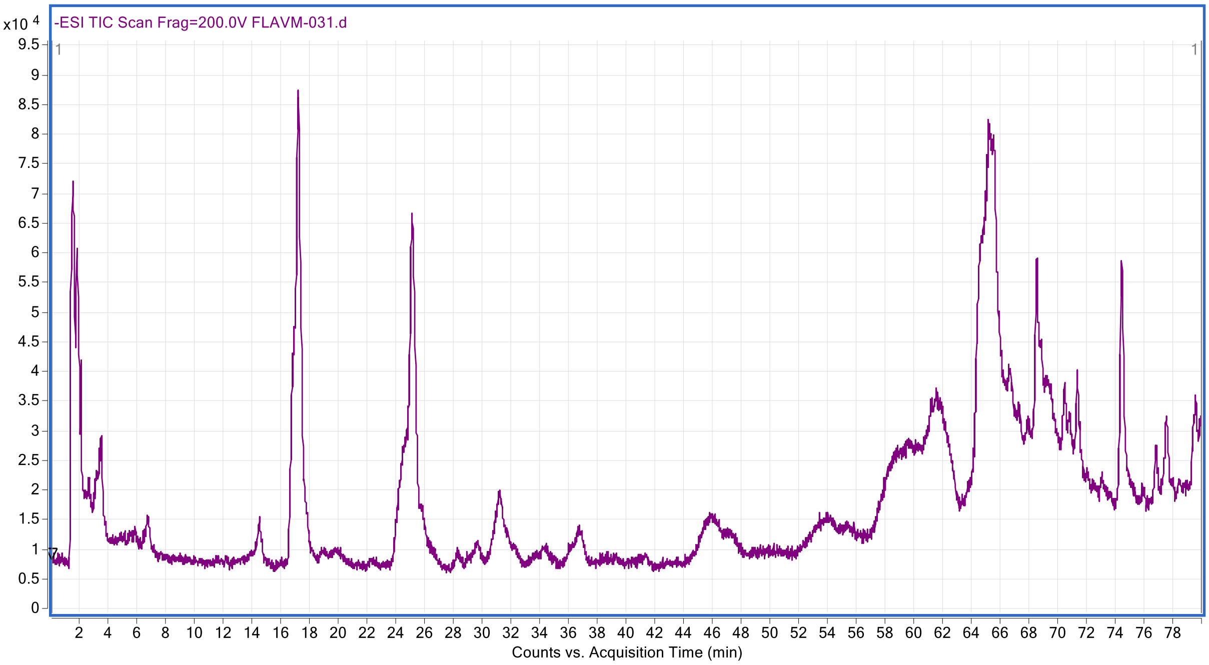

F.- HPLC-TOF-MS chromatogram of fraction five (unidentified compounds).

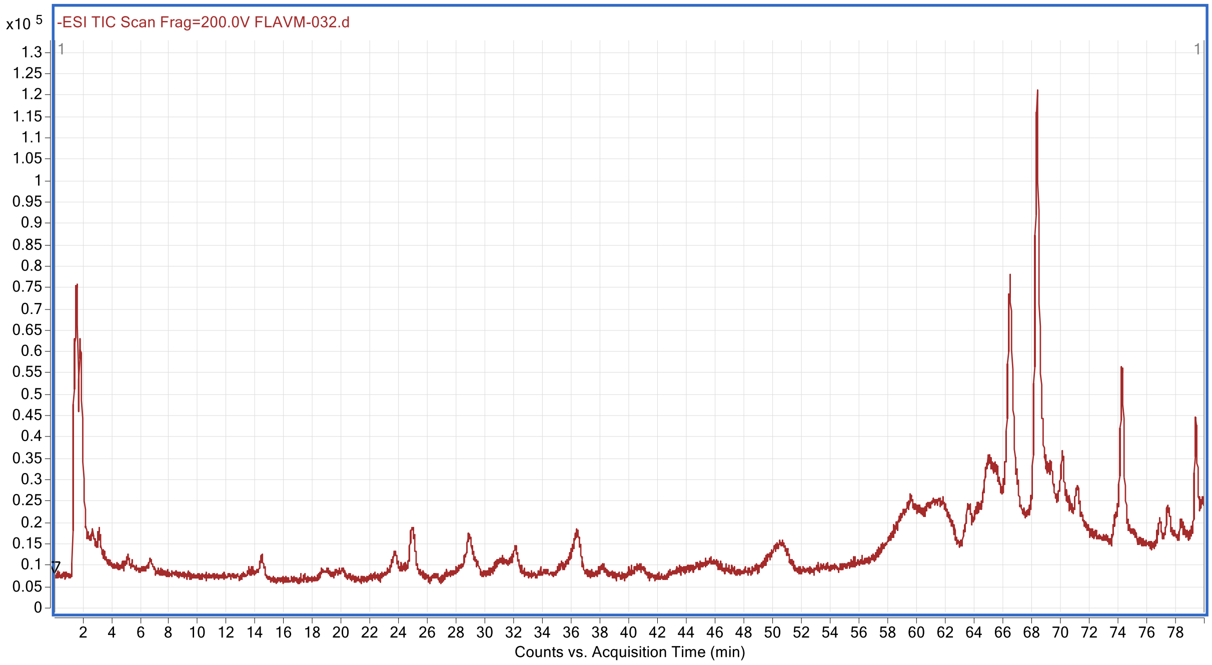

G.- HPLC-TOF-MS chromatogram of fraction six (unidentified compounds).

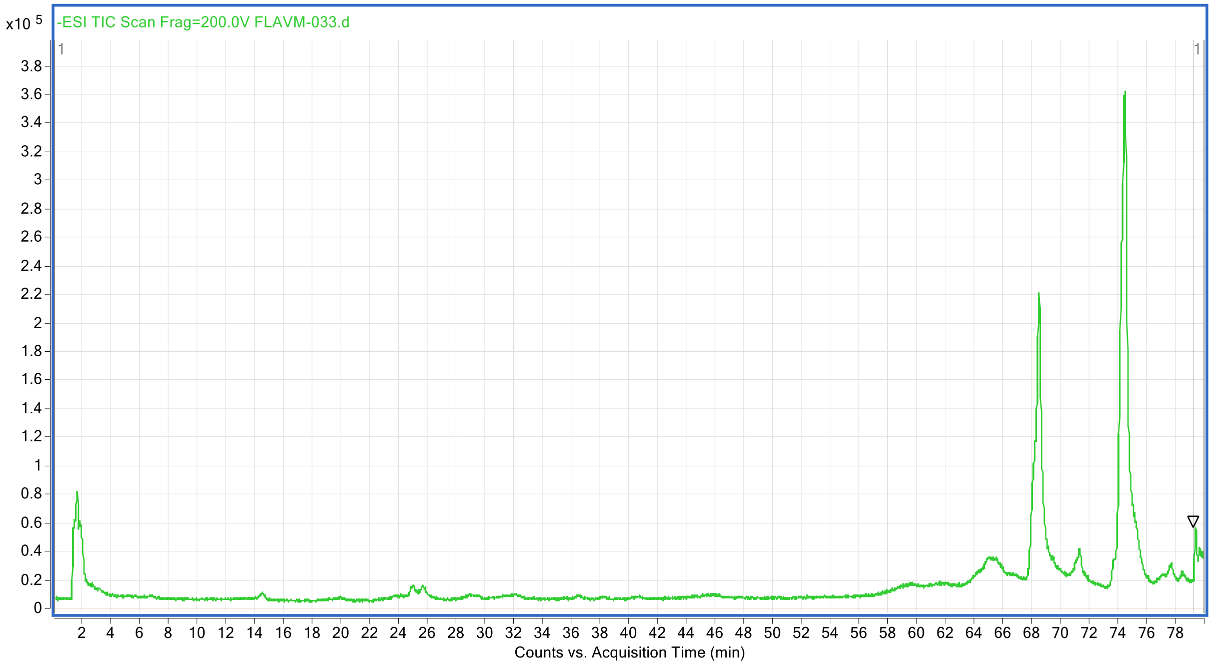

H.- HPLC-TOF-MS chromatogram of fraction seven (unidentified compounds).

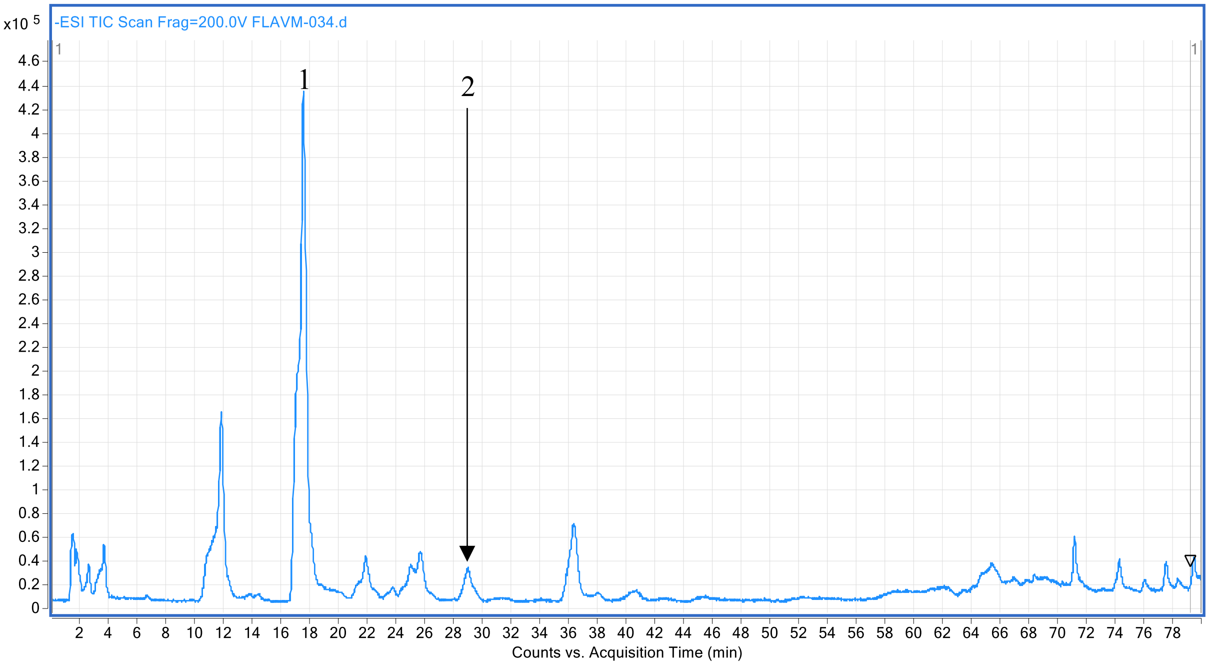

I.- HPLC-TOF-MS chromatogram of fraction eight (apigenin and chrysin).

1.- Apigenin

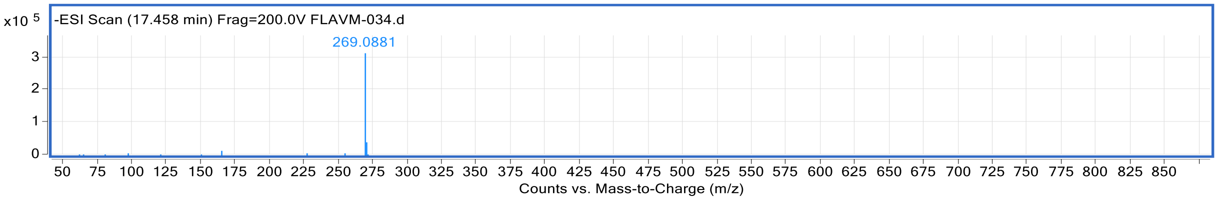

Standard

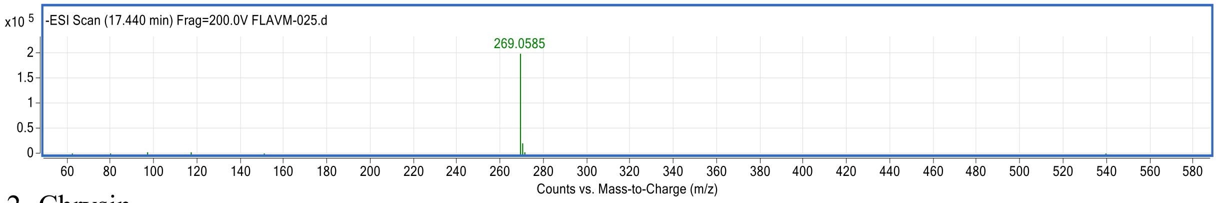

2.-Chrysin

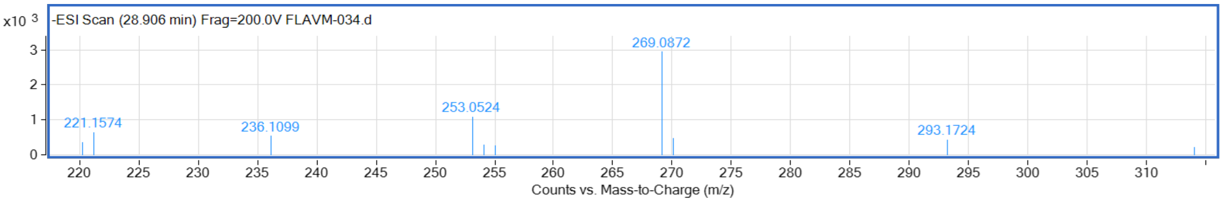

Standard

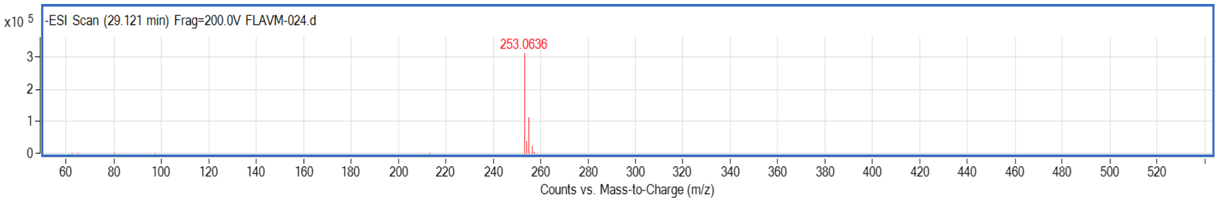

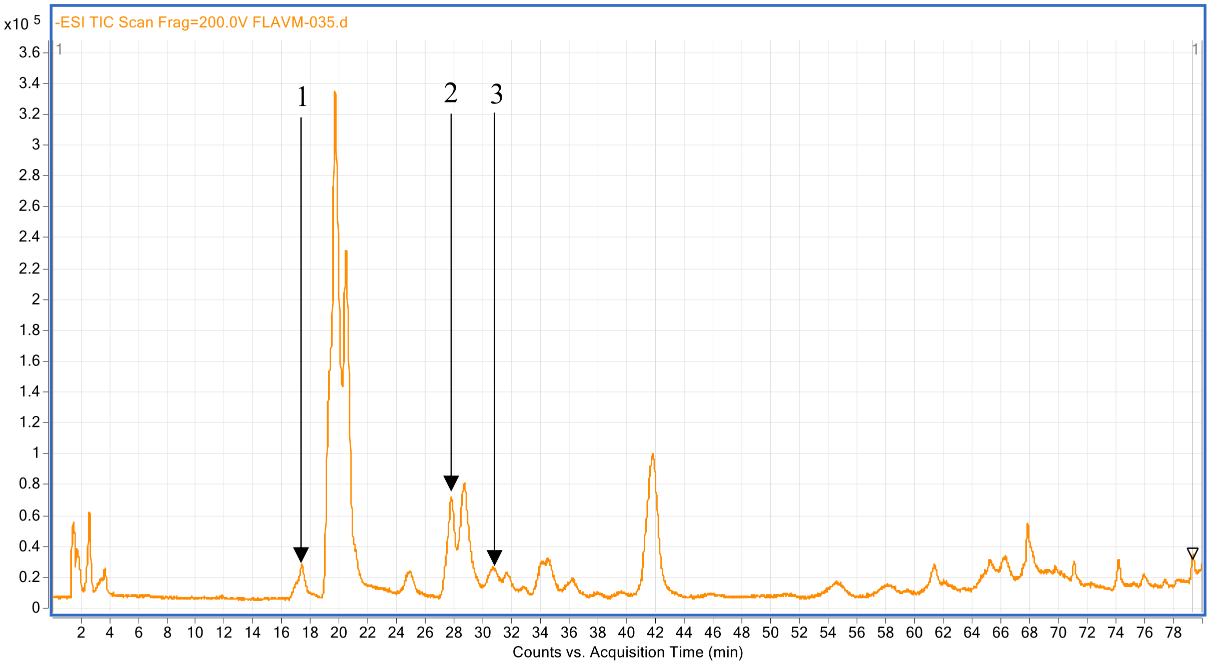

J.- HPLC-TOF-MS chromatogram of fraction nine (genistein, chrysin, and acacetin).

1.- Genistein

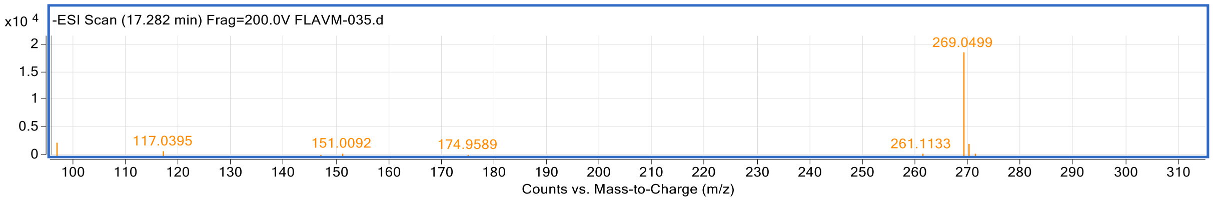

Standard

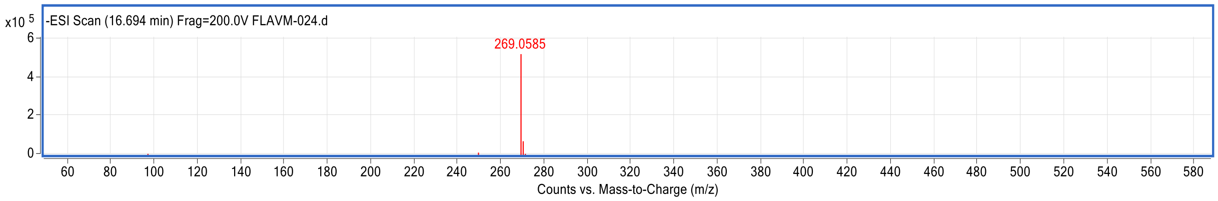

2.- Chrysin

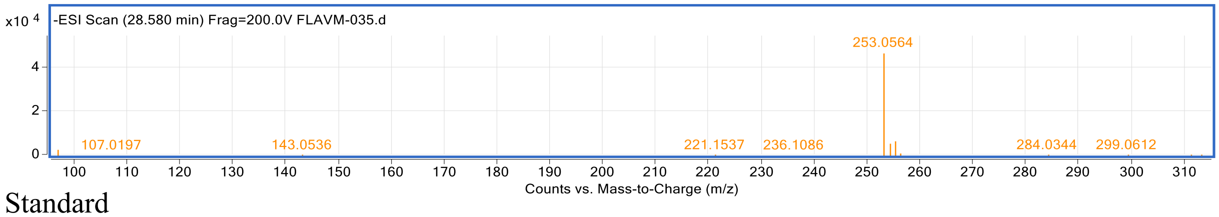

Standard

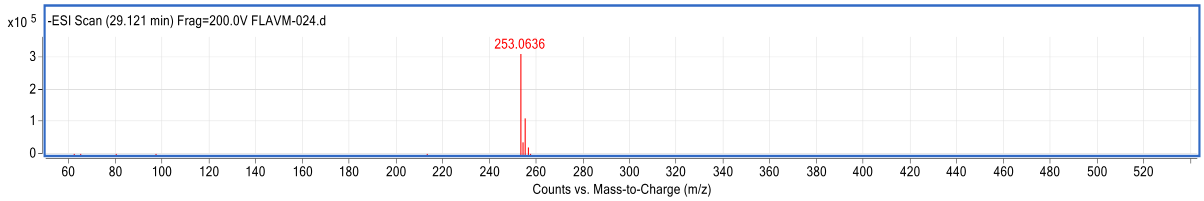

3.-Acacetin

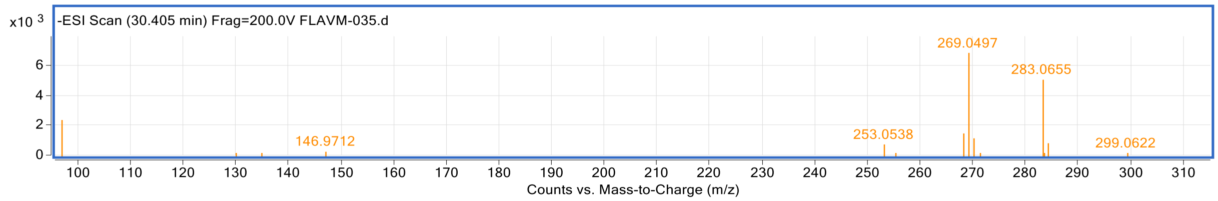

Standard

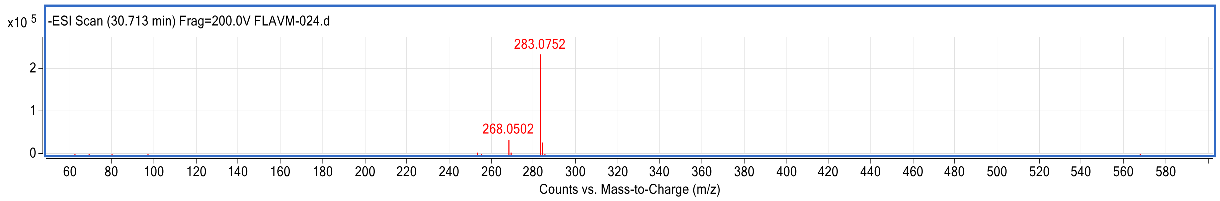

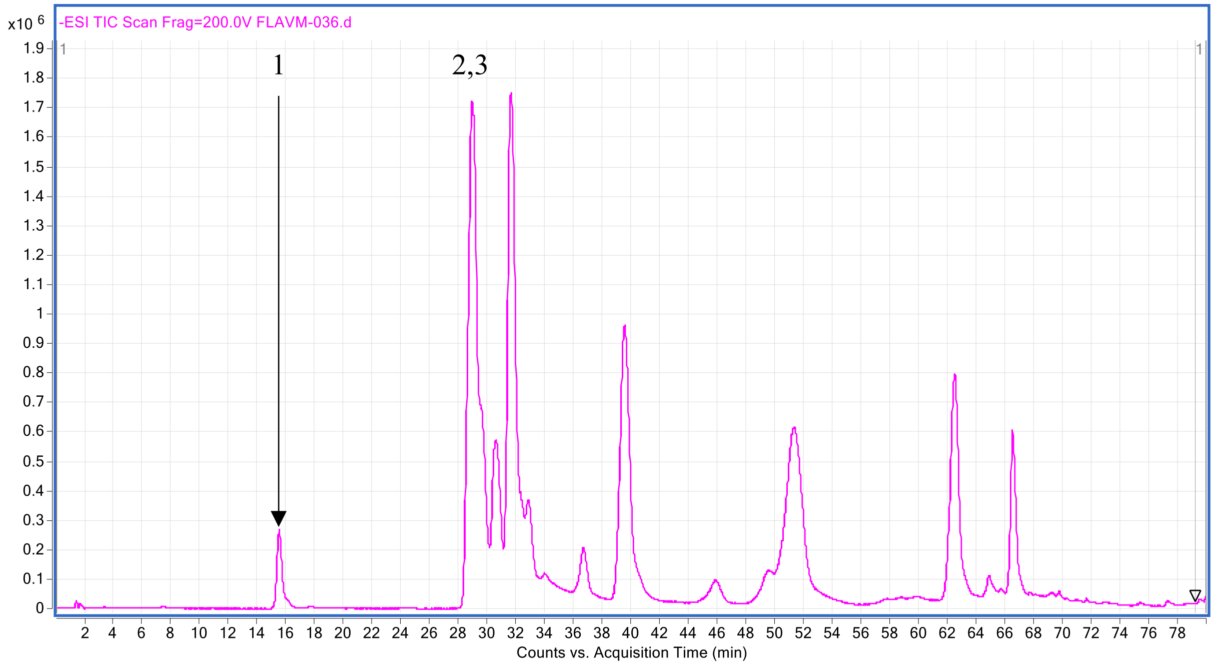

K.- HPLC-TOF-MS chromatogram of fraction ten (naringenin, chrysin and pinocembrin).

1.-Naringenin

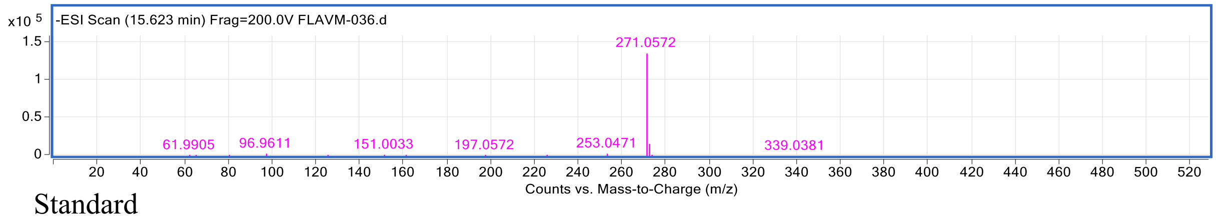

Standard

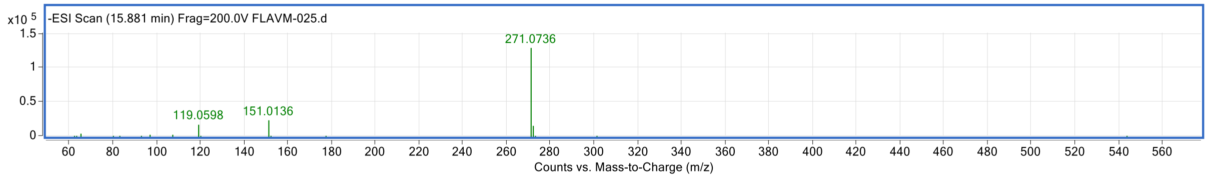

2.-Chrysin

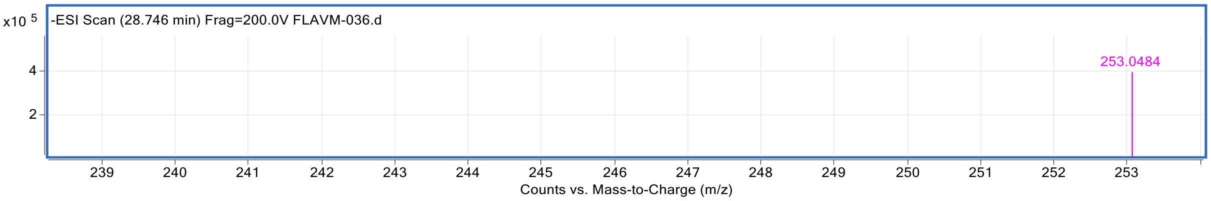

Standard

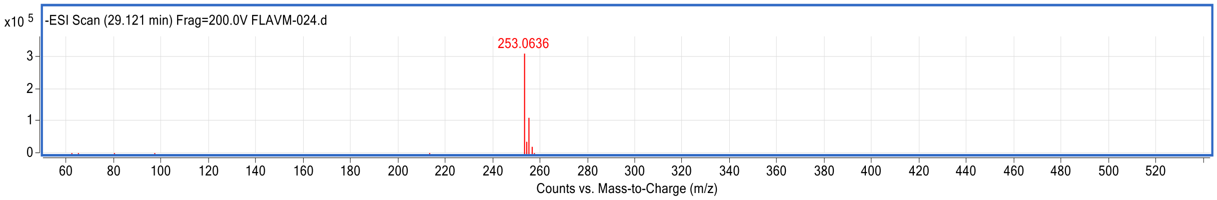

3.- Pinocembrin

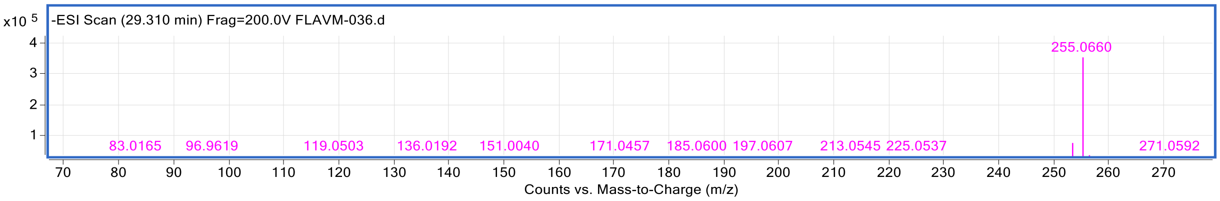

Standard

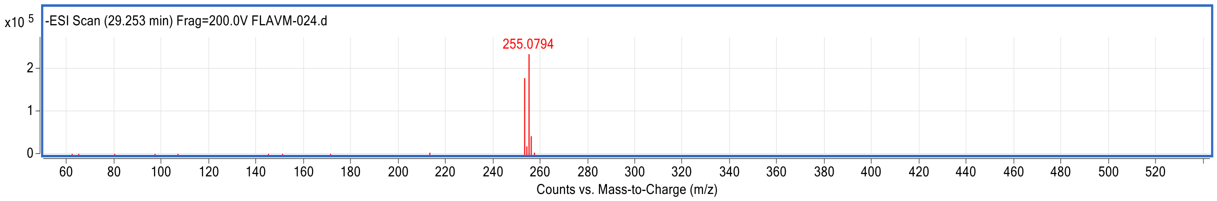

Compounds identified in the ethanolic extract of Mexicali propolis by GC-MS analysis.

Figure S2. GC-MS chromatograms of the MeEEP, EAF, MeEAEP and MeHEP. Mass spectra of compounds identified in the ethanolic extract of Mexicali propolis by GC-MS analysis. For each compound identified in the Mexicali propolis extracts, the mass spectrum of the unknown compound (obtained by GC-MS), the comparison of the mass spectrum of the unknown compound (red) with the NIST 8.0 library standard (blue) and the mass spectrum of the library standard with its molecular structure are presented.

Mexicali ethanolic extract of propolis

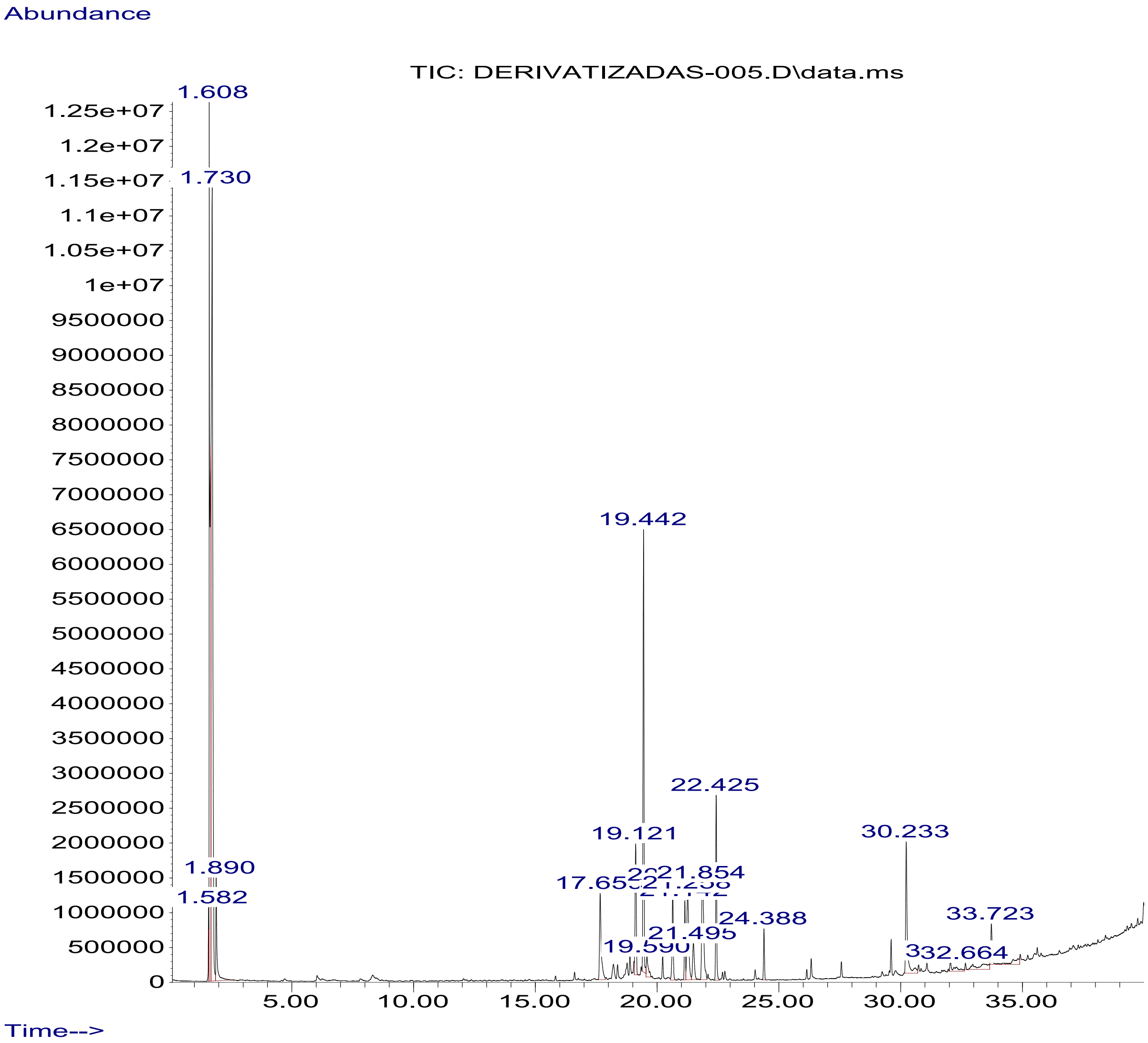

A.- GC-MS chromatogram of the MeEEP

1.- Xylonic acid

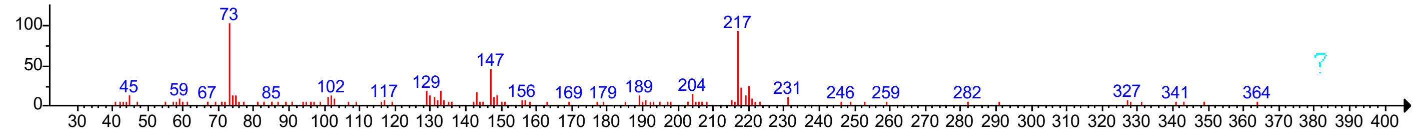

(Text File) Scan 2454 (15.830 min): DERIVATIZADAS-005.D\ data.ms (-2465)

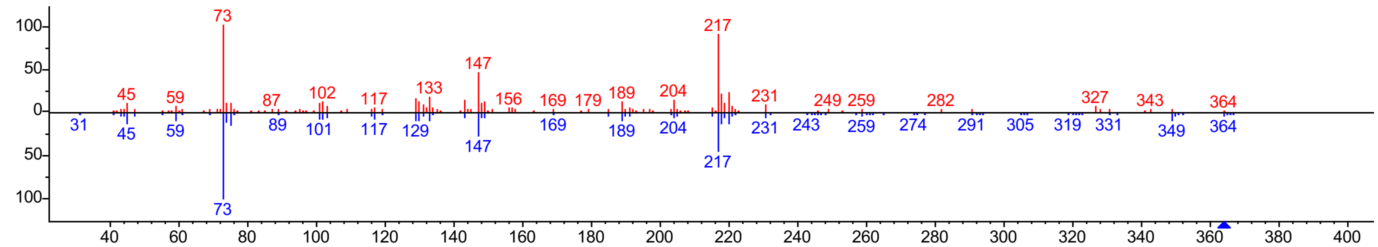

Scan 2454 (15.830 min): DERIVATIZADAS-005.D\ data.ms | Head to Tail MF=769 RMF=882 | Xylonic acid, 2,3,4-tris-O-(trimethylsilyl)-, d-lactone, D-

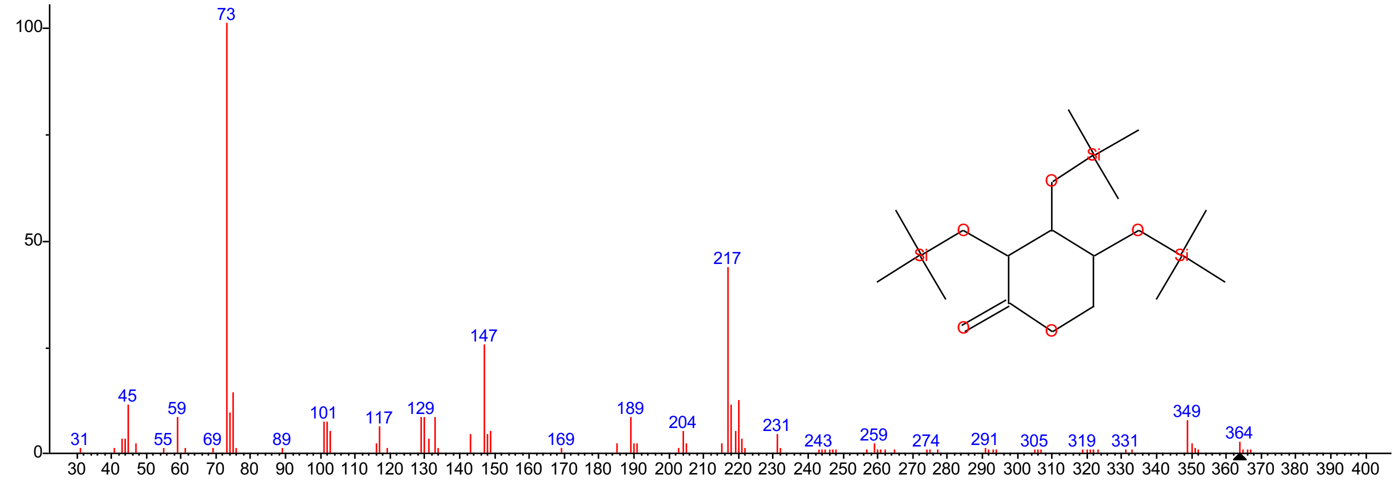

(mainlib) Xylonic acid, 2,3,4-tris-O-(trimethylsilyl)-, d-lactone, D-

2.- α-D-Glucopyranose

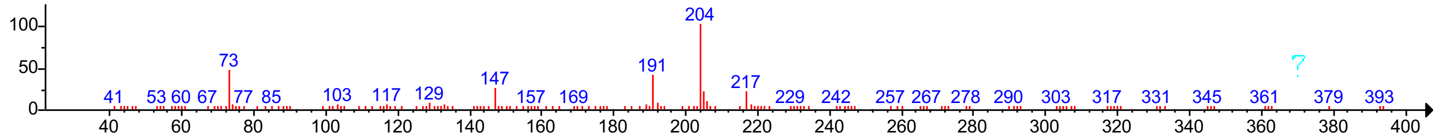

(Text File) Scan 3204 (20.642 min): DERIVATIZADAS-005.D\ data.ms (-3224)

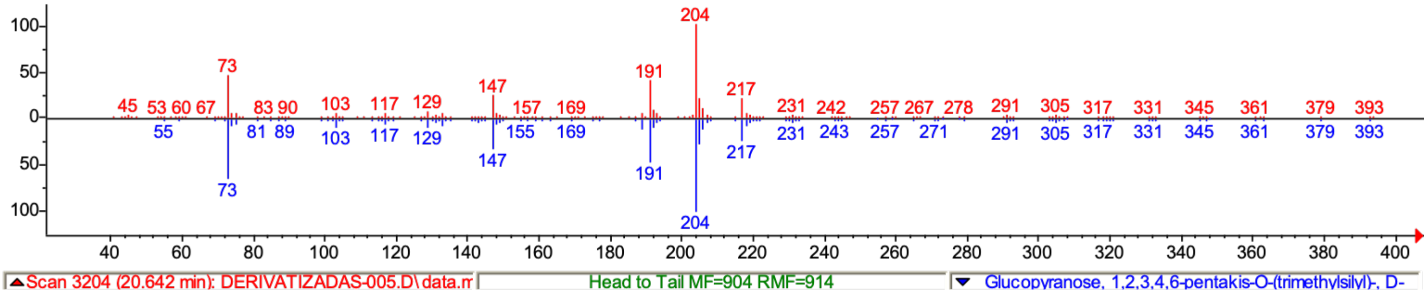

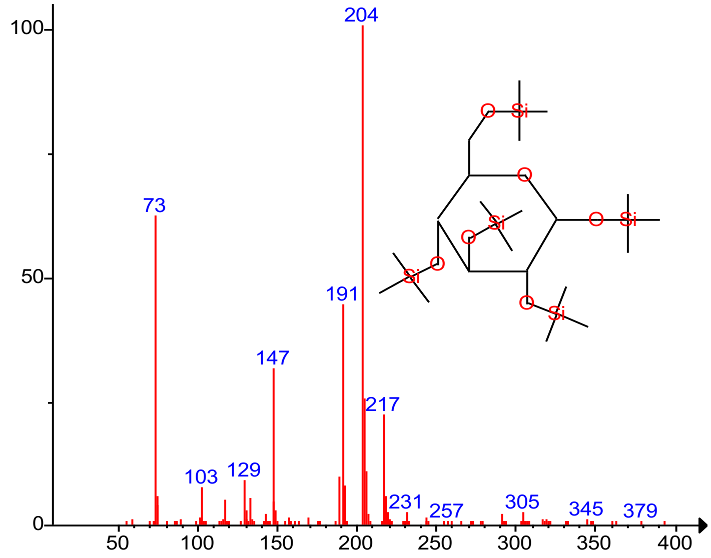

(mainlib) Glucopyranose, 1,2,3,4,6-pentakis-O-(trimethylsilyl)-, D-

3.- β-D-Glucopyranose

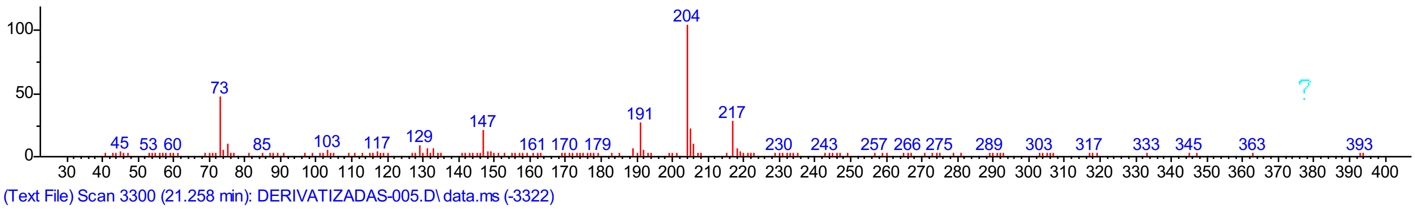

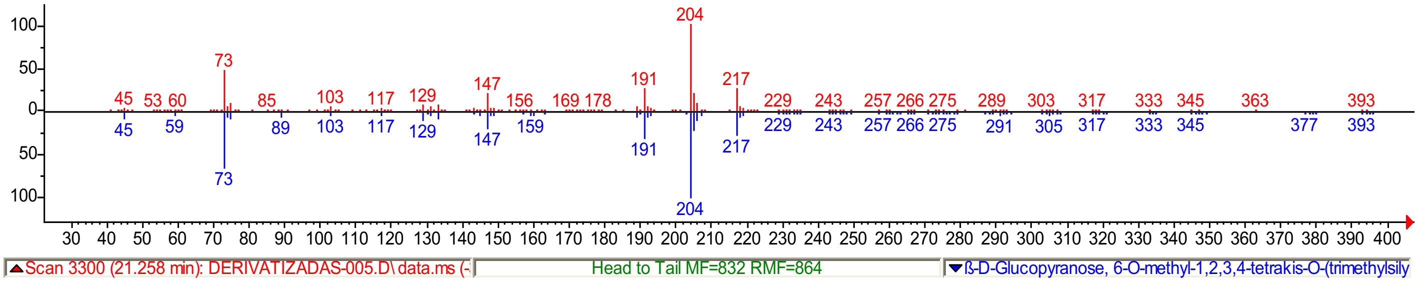

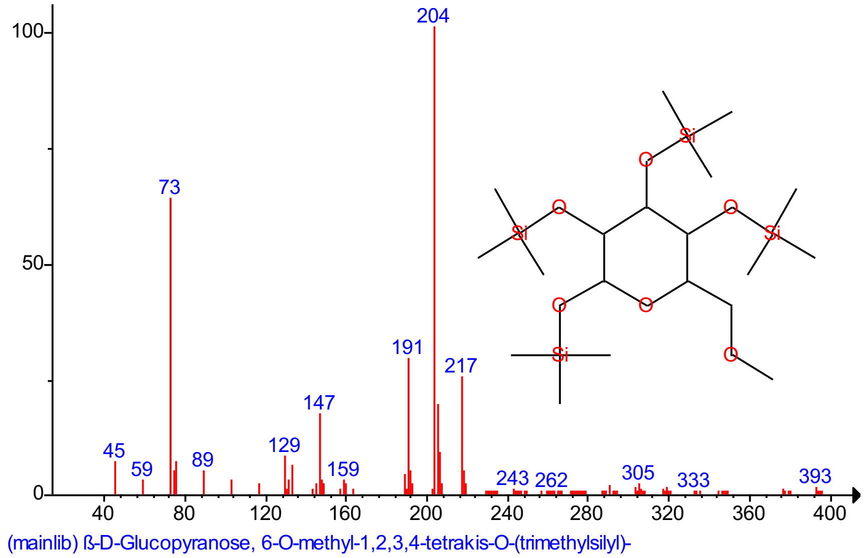

4.- 2- Nonadecanone

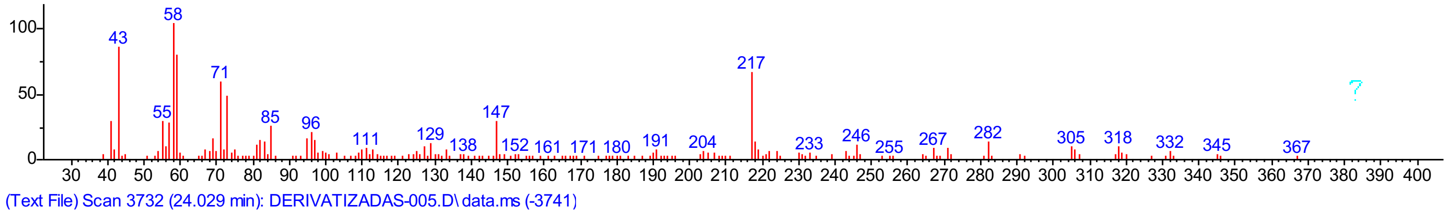

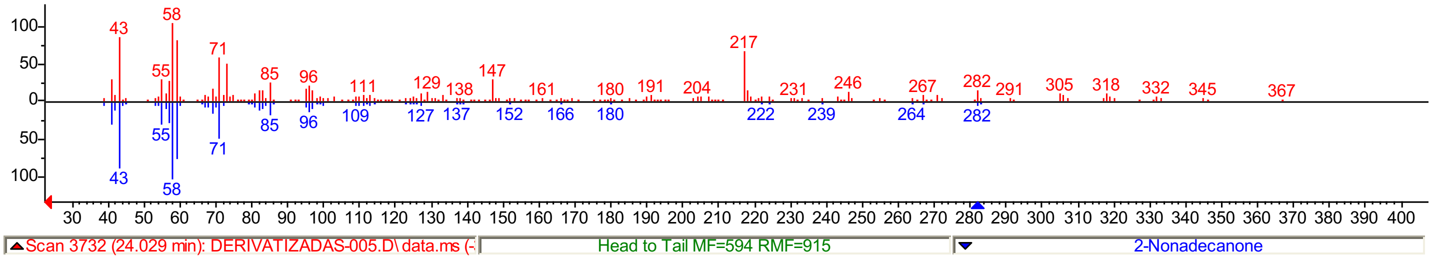

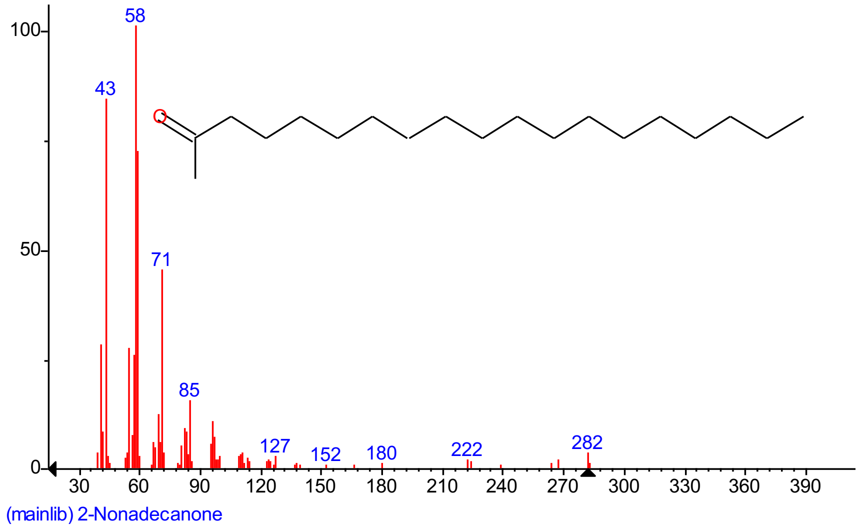

5.- Inositol

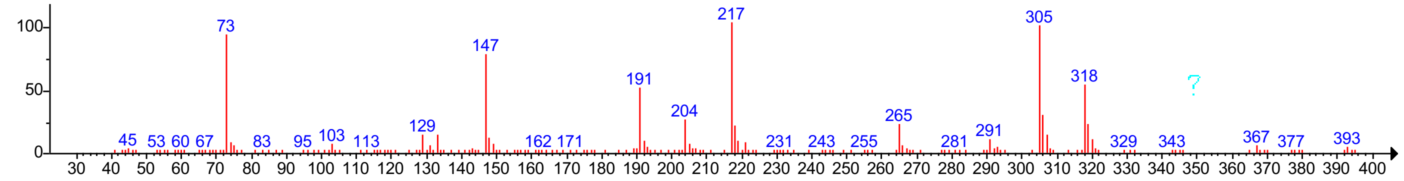

(Text File) Scan 3789 (24.395 min): DERIVATIZADAS-005.D\ data.ms (-3799)

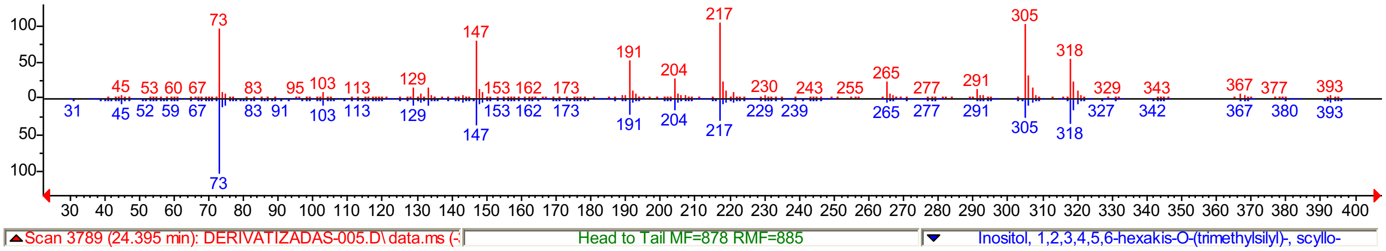

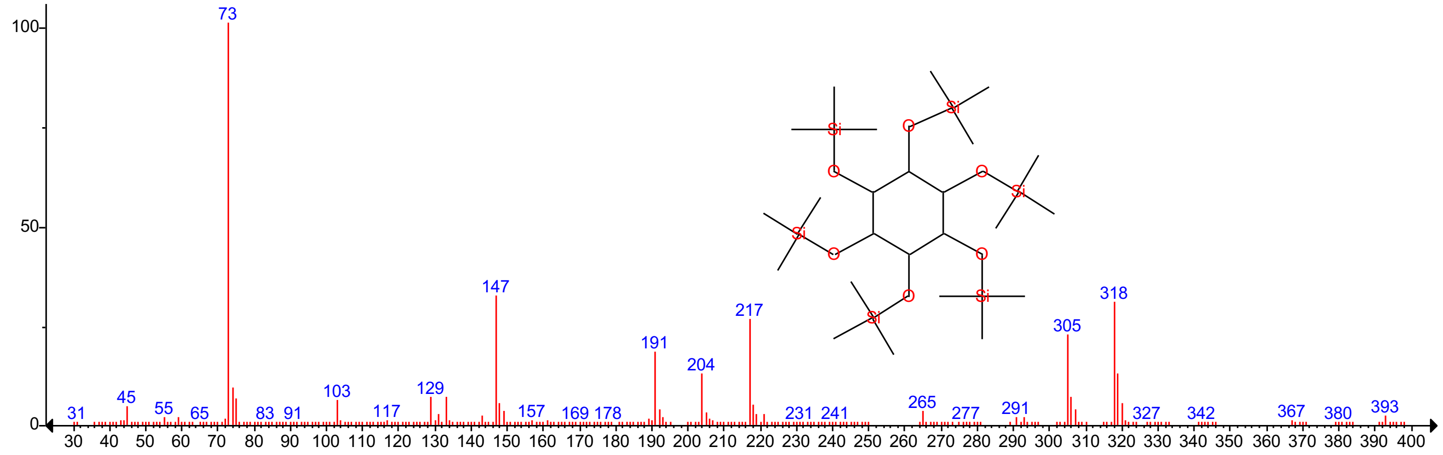

(replib) Inositol, 1,2,3,4,5,6-hexakis-O-(trimethylsilyl)-, scyllo-

6.- Pinocembrin

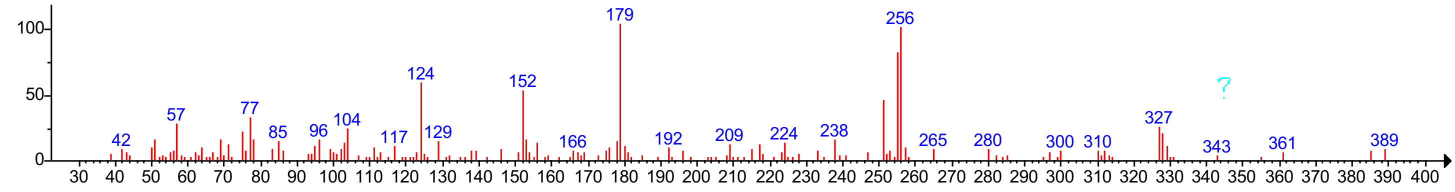

(Text File) Scan 4756 (30.598 min): DERIVATIZADAS-005.D\ data.ms (-4769)

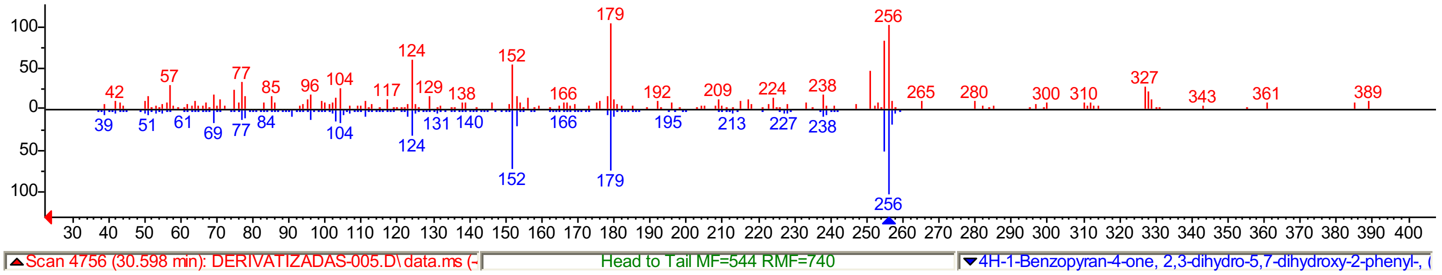

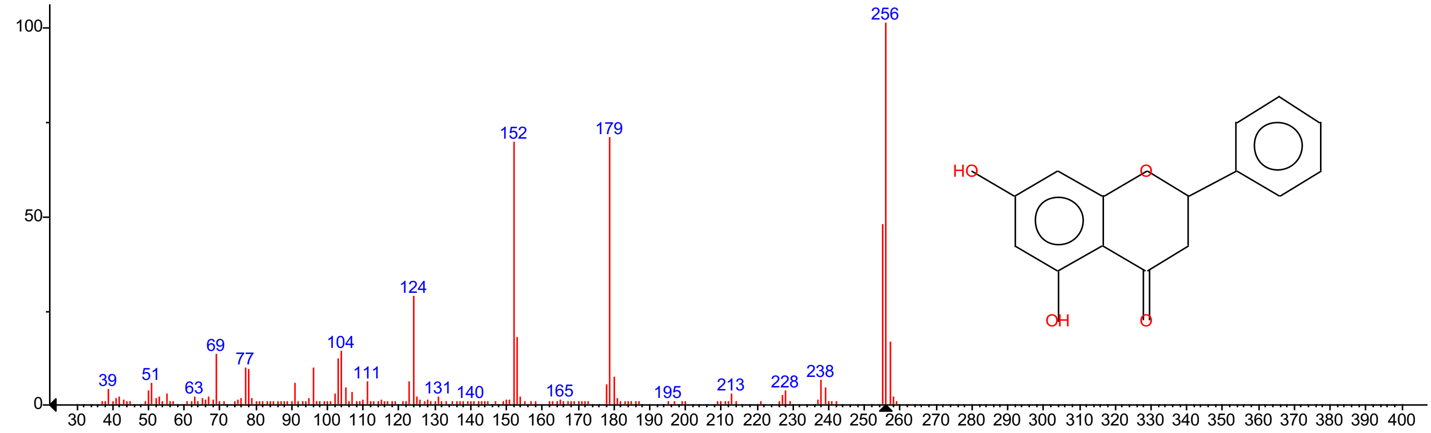

(mainlib) 4H-1-Benzopyran-4-one, 2,3-dihydro-5,7-dihydroxy-2-phenyl-, (S)-

Ethyl acetate fraction of Mexicali ethanolic extract of propolis

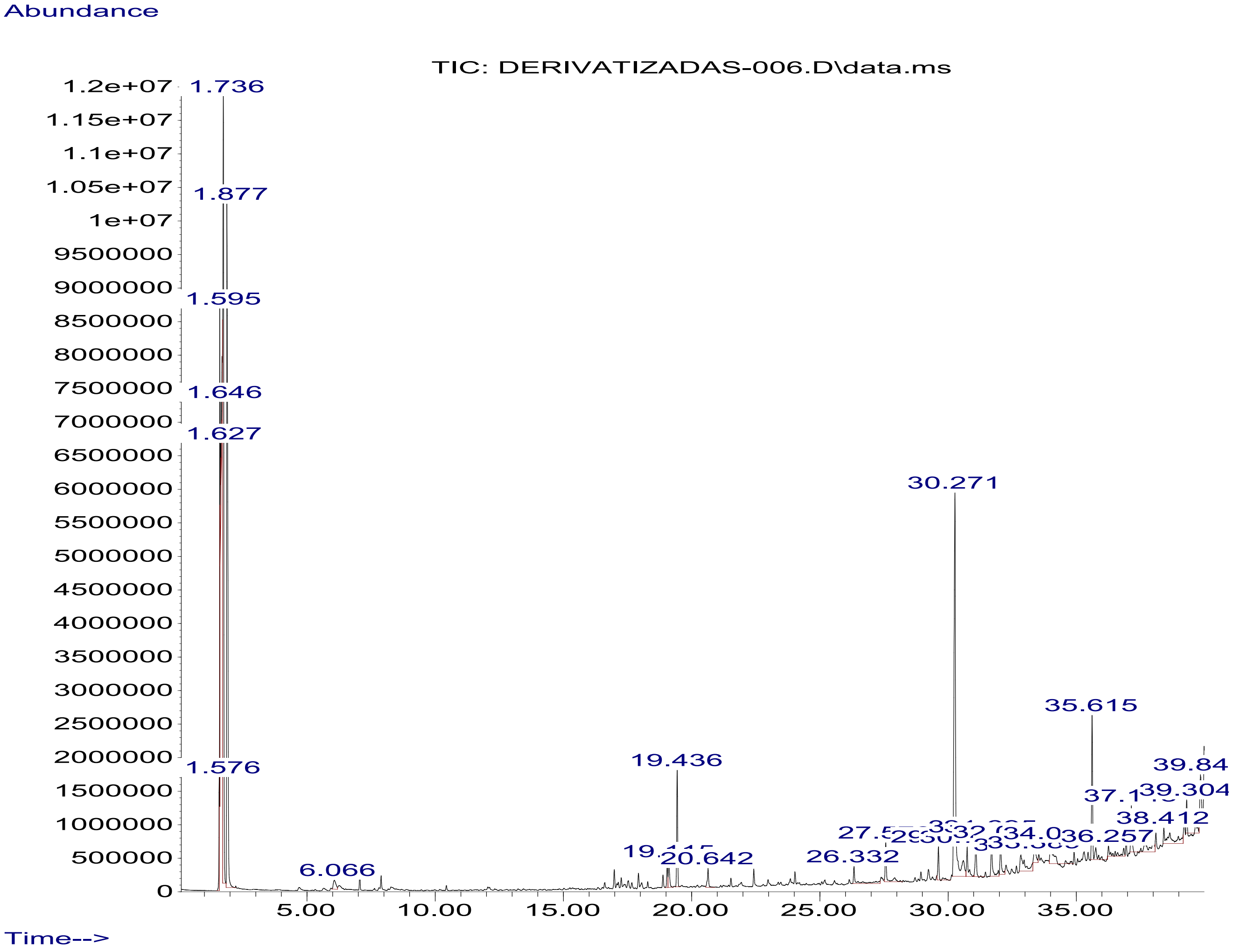

B.- GC-MS chromatogram of the EAF

1.- β-Galactopyranose

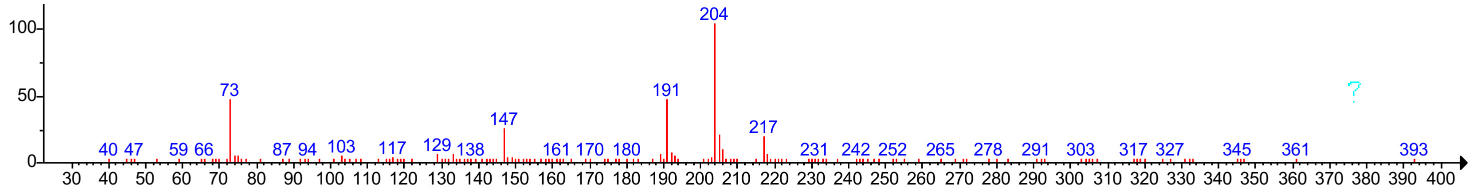

(Text File) Scan 3482 (22.425 min): DERIVATIZADAS-006.D\ data.ms (-3499)

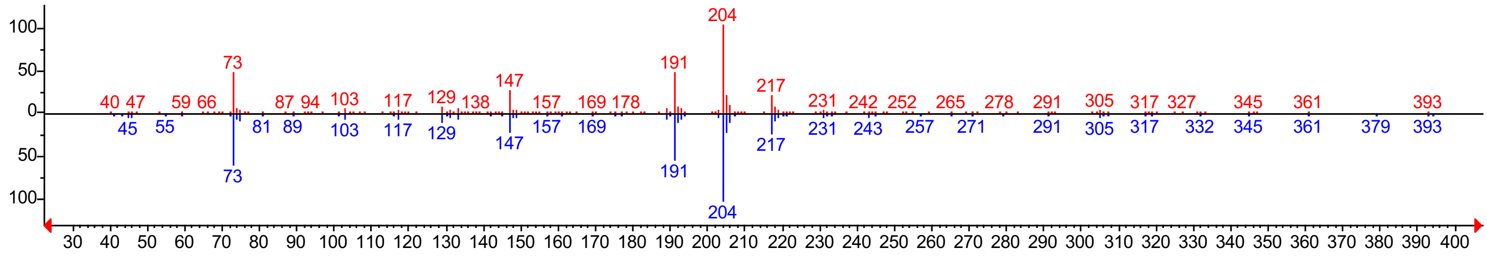

Scan 3482 (22.425 min): DERIVATIZADAS-006.D\ data.ms (- | Head to Tail MF=886 RMF=936 | Galactopyranose, 1,2,3,4,6-pentakis-O-(trimethylsilyl)-, ß-d-

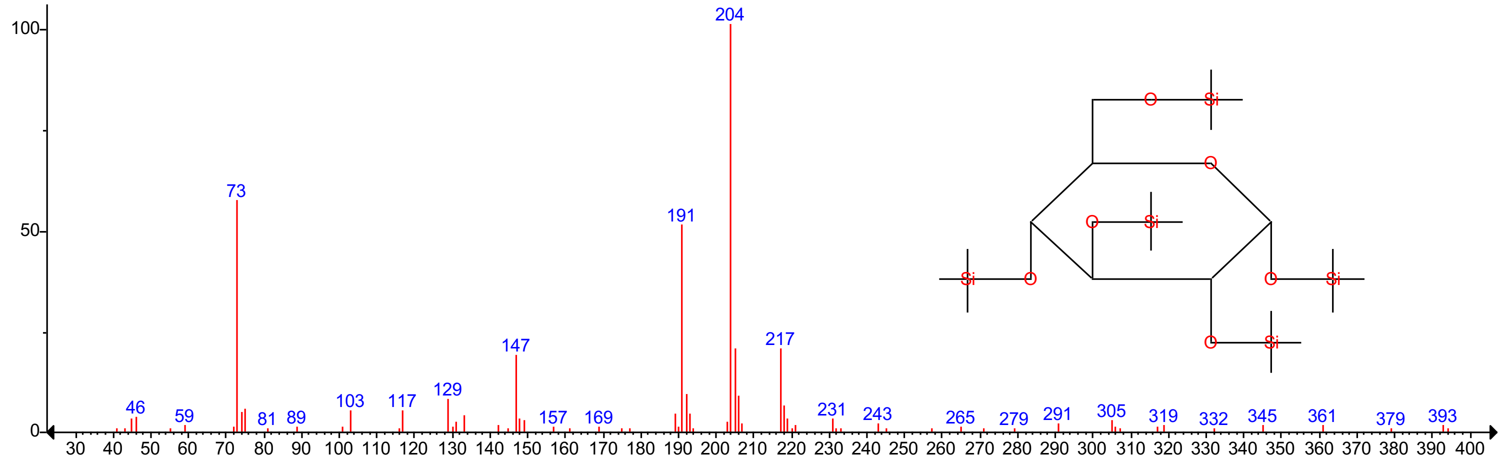

(replib) Galactopyranose, 1,2,3,4,6-pentakis-O-(trimethylsilyl)-, ß-d-

2.- 2-Nonadecanone

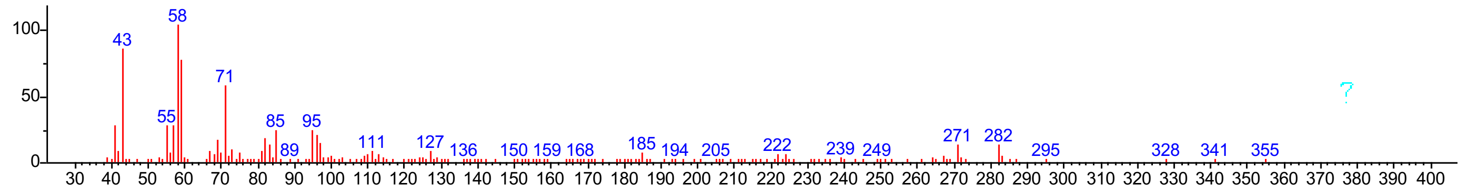

(Text File) Scan 3733 (24.036 min): DERIVATIZADAS-006.D\ data.ms (-3740)

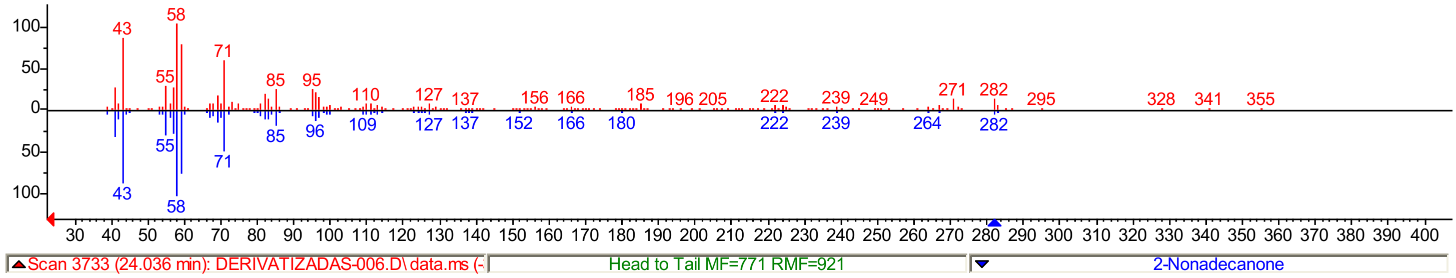

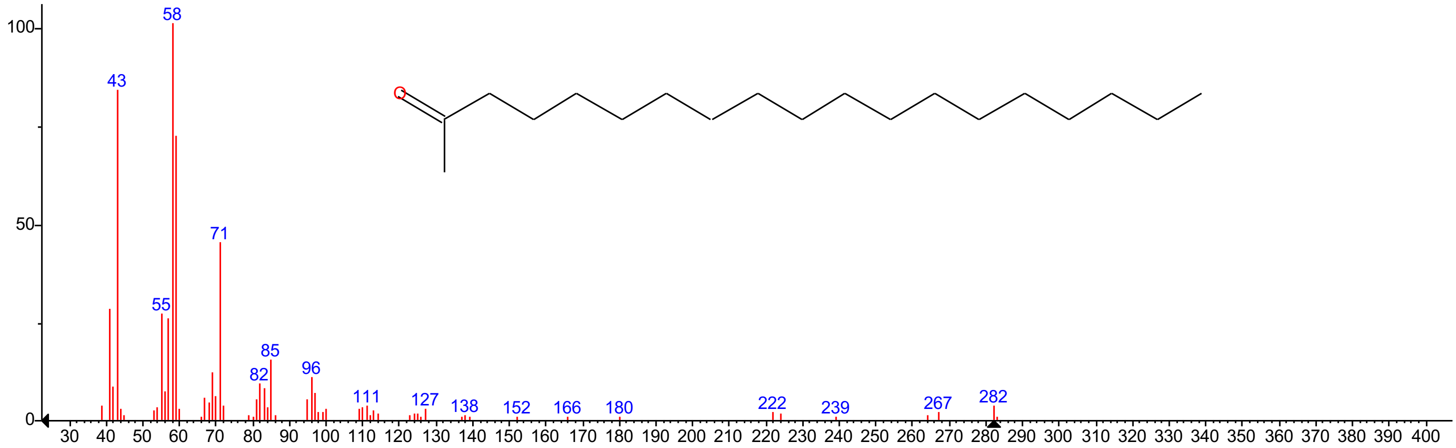

(mainlib) 2-Nonadecanone

3.-Pinostrobin chalcone

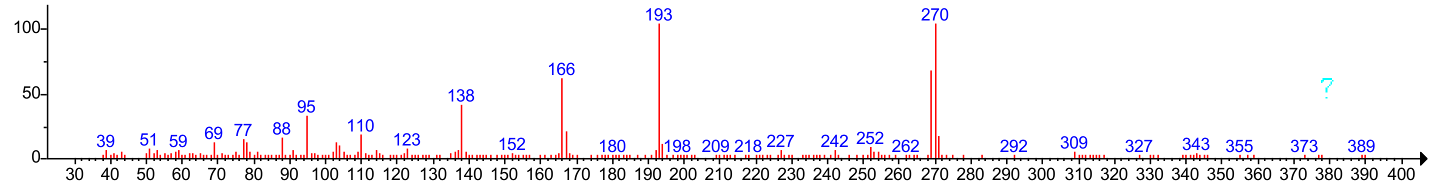

(Text File) Scan 4544 (29.238 min): DERIVATIZADAS-006.D\ data.ms (-4558)

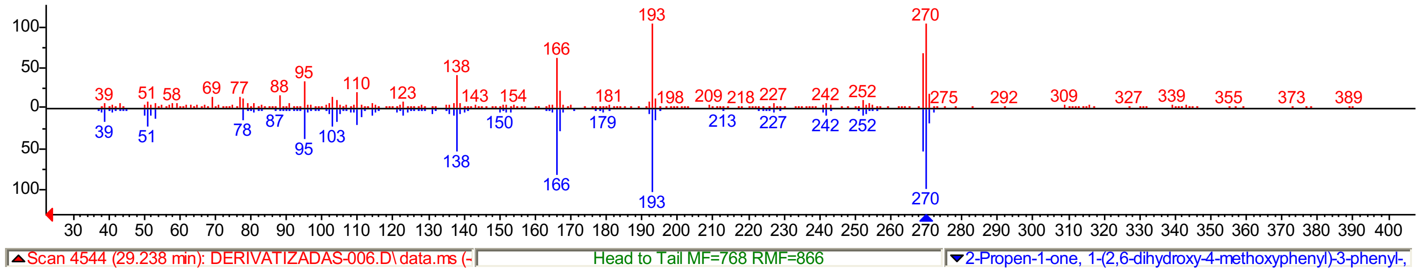

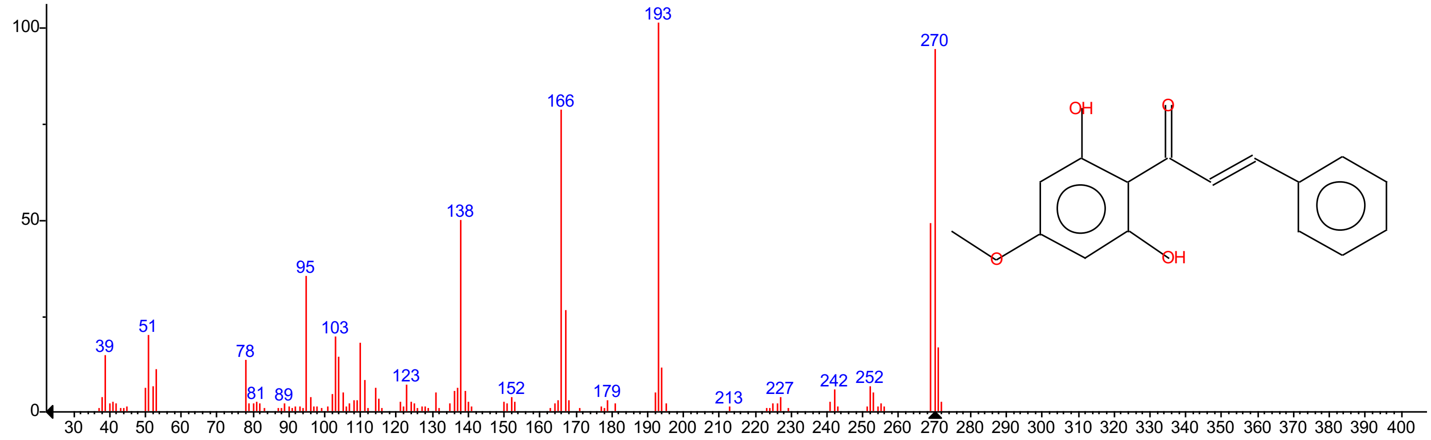

(mainlib) 2-Propen-1-one, 1-(2,6-dihydroxy-4-methoxyphenyl)-3-phenyl-, (E)-

4.- Pinocembrin

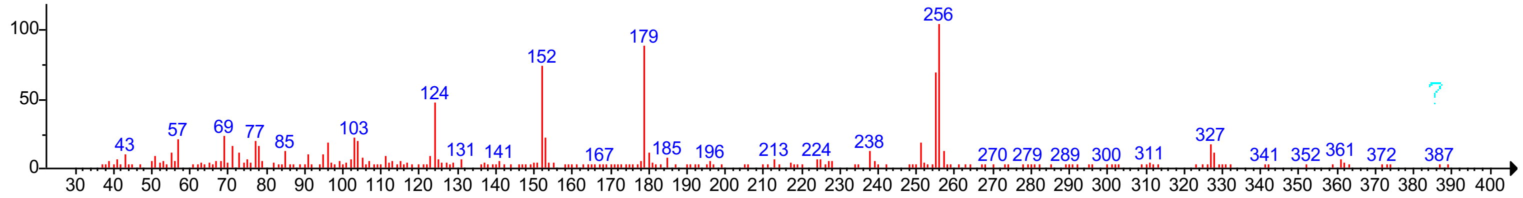

(Text File) Scan 4756 (30.598 min): DERIVATIZADAS-006.D\ data.ms (-4770)

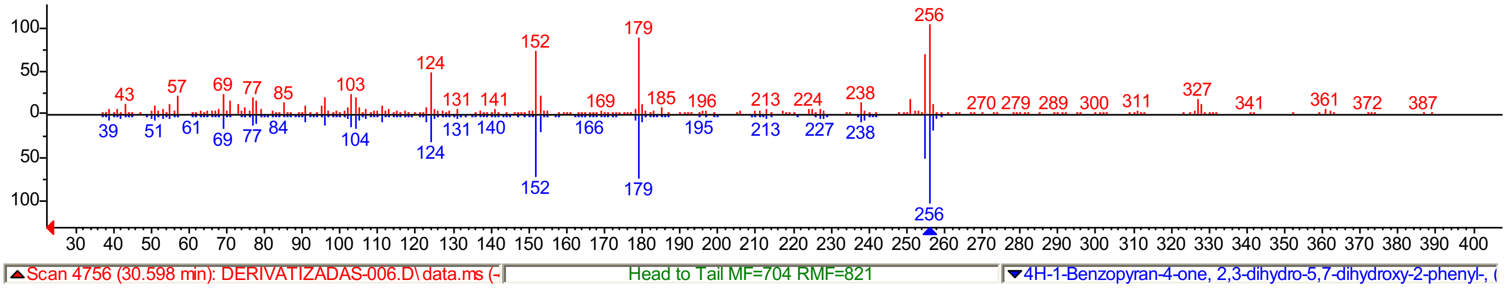

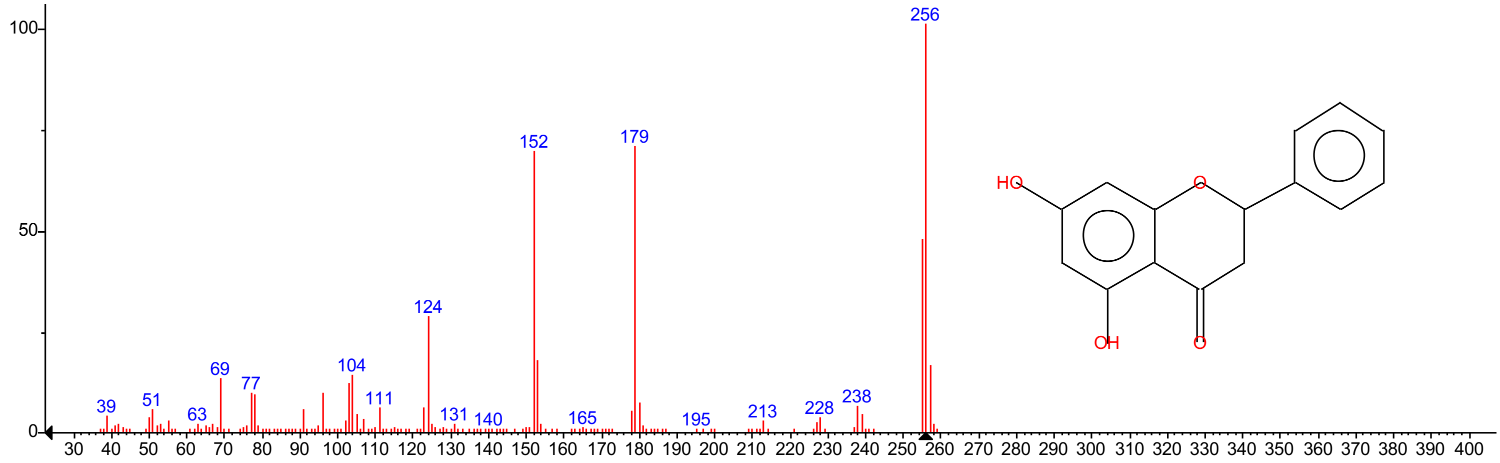

(mainlib) 4H-1-Benzopyran-4-one, 2,3-dihydro-5,7-dihydroxy-2-phenyl-, (S)-

5.- Tectochrysin

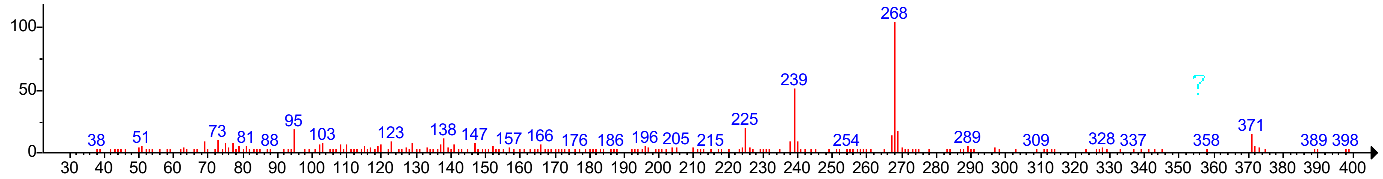

(Text File) Scan 5016 (32.266 min): DERIVATIZADAS-006.D\ data.ms (-5027)

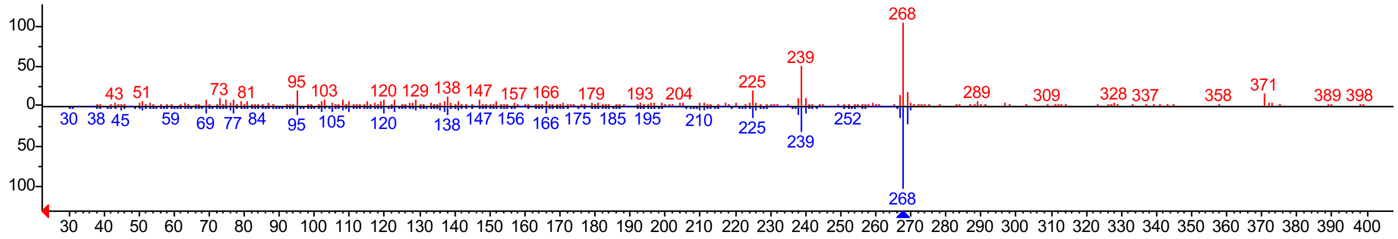

Scan 5016 (32.266 min): DERIVATIZADAS-006.D\ data.ms (- | Head to Tail MF=669 RMF=794 | 4H-1-Benzopyran-4-one, 5-hydroxy-7-methoxy-2-phenyl-

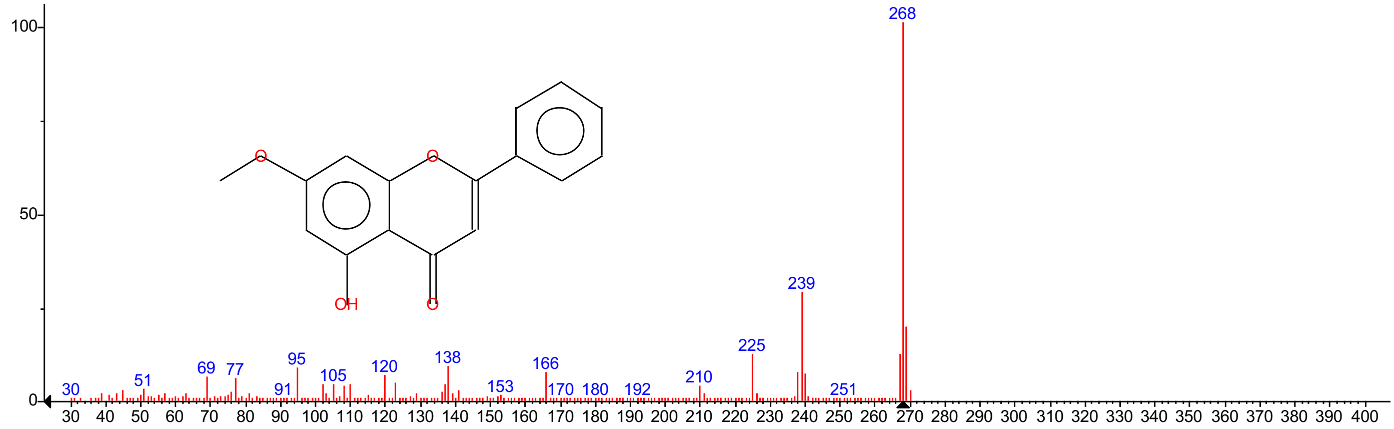

(mainlib) 4H-1-Benzopyran-4-one, 5-hydroxy-7-methoxy-2-phenyl-

Mexicali ethyl acetate extract of propolis

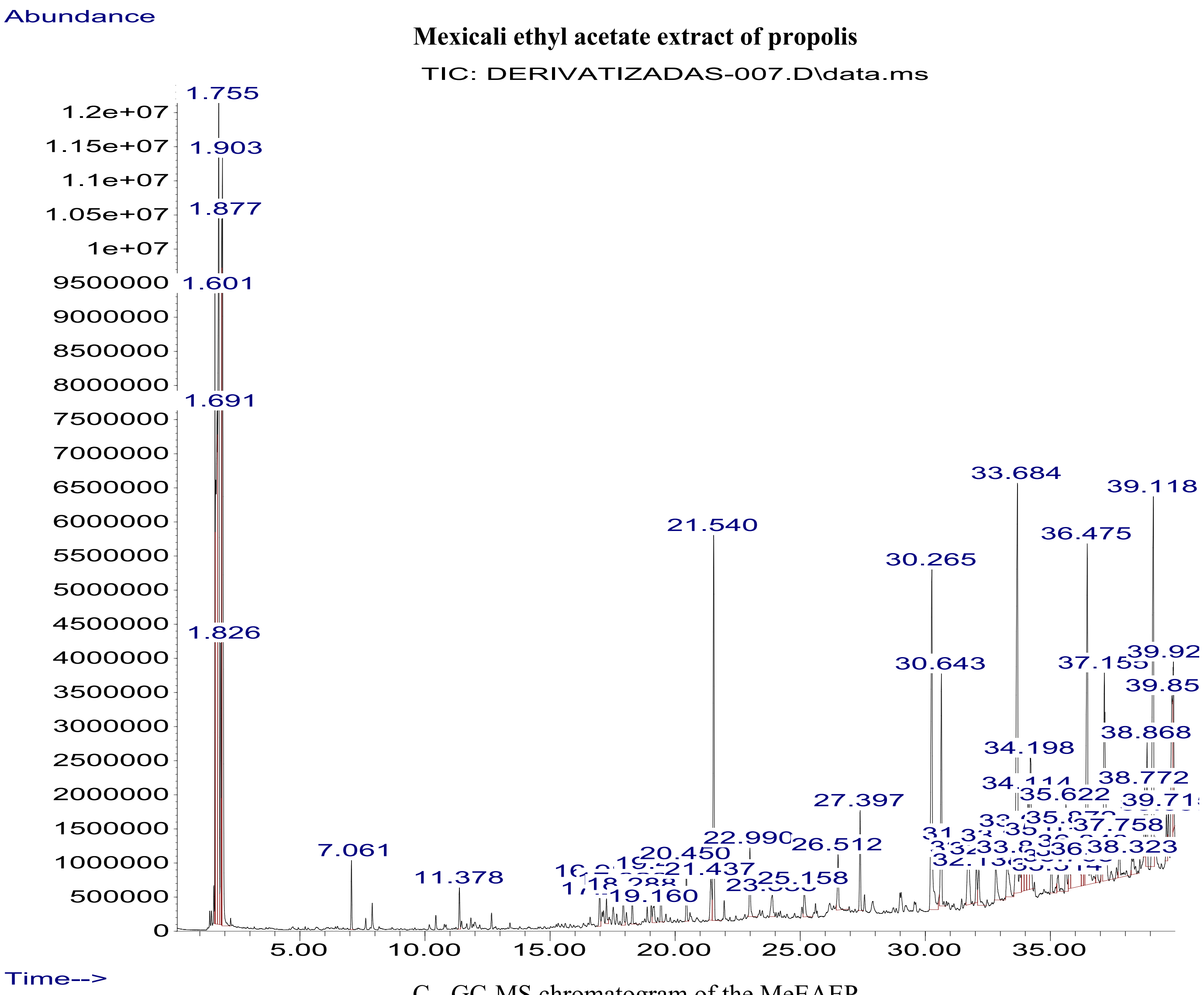

C.- GC-MS chromatogram of the MeEAEP

1.- Benzyl alcohol

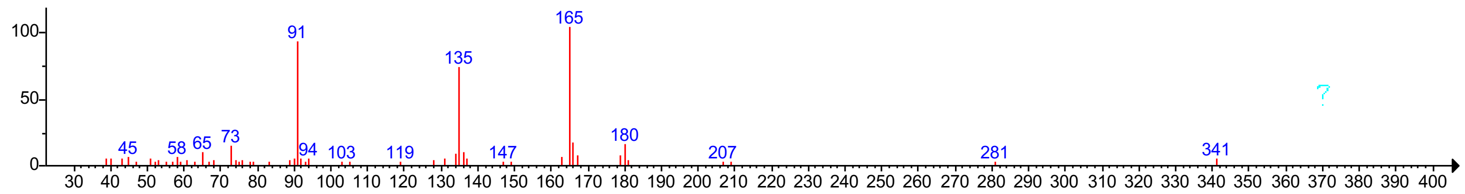

(Text File) Scan 756 (4.937 min): DERIVATIZADAS-007.D\ data.ms (-760)

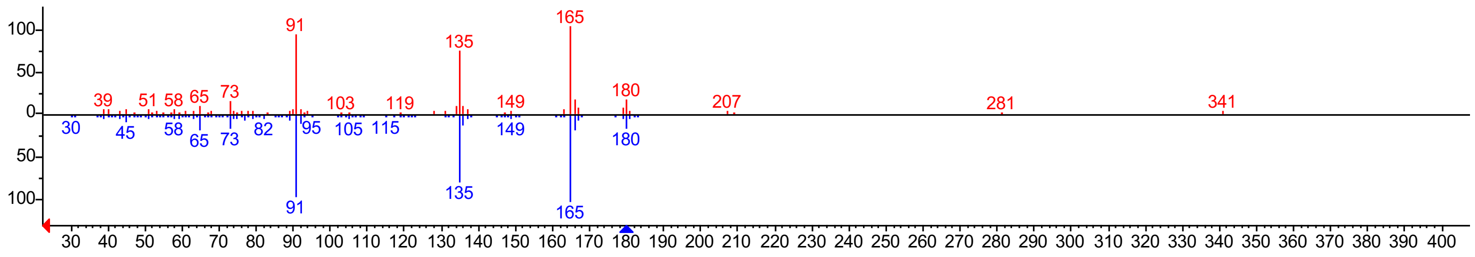

Scan 756 (4.937 min): DERIVATIZADAS-007.D\ data.ms (-76 | Head to Tail MF=807 RMF=870 | Silane, trimethyl(phenylmethoxy)-

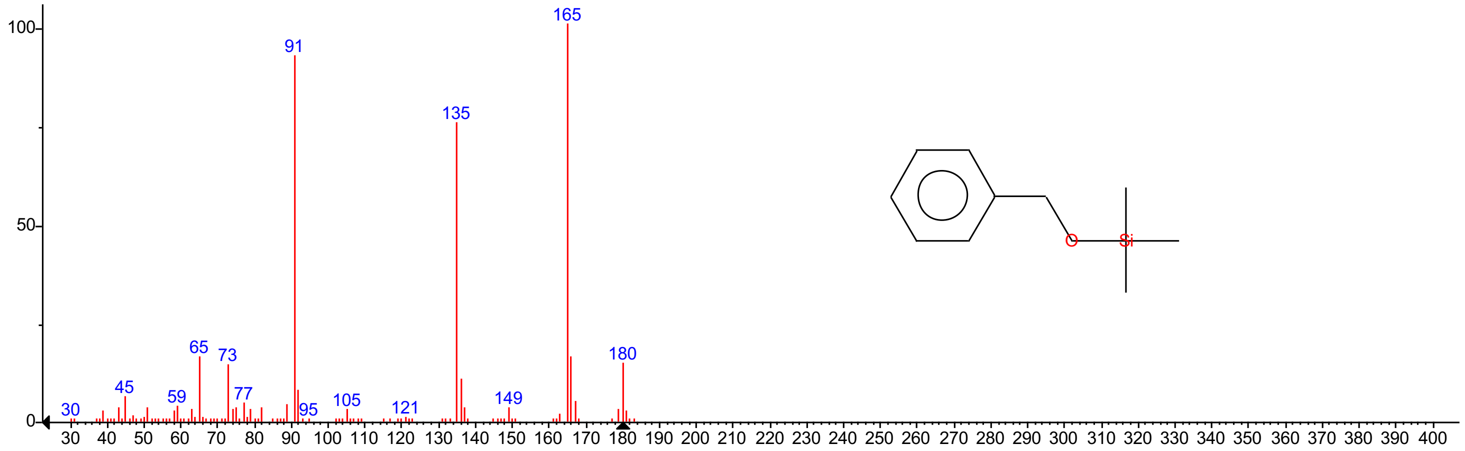

(mainlib) Silane, trimethyl(phenylmethoxy)-

2.-Benzoic Acid

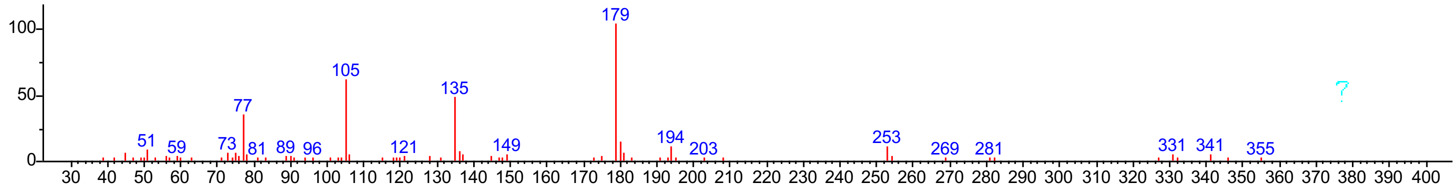

(Text File) Scan 1000 (6.503 min): DERIVATIZADAS-007.D\ data.ms (-1007)

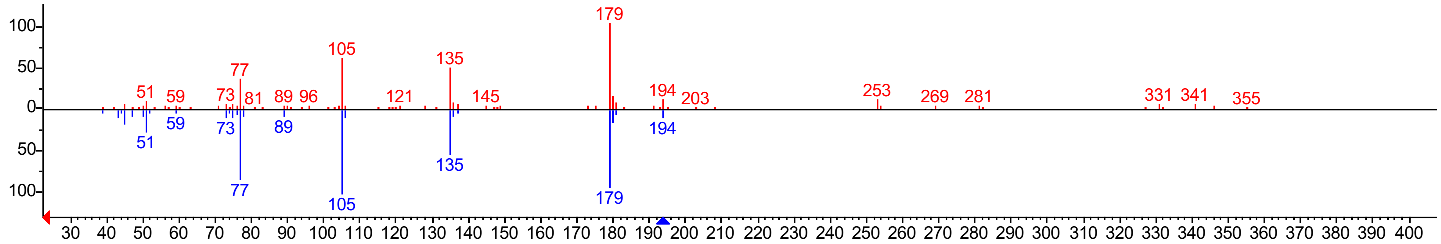

Scan 1000 (6.503 min): DERIVATIZADAS-007.D\ data.ms (-1 | Head to Tail MF=748 RMF=916 | Benzoic acid trimethylsilyl ester

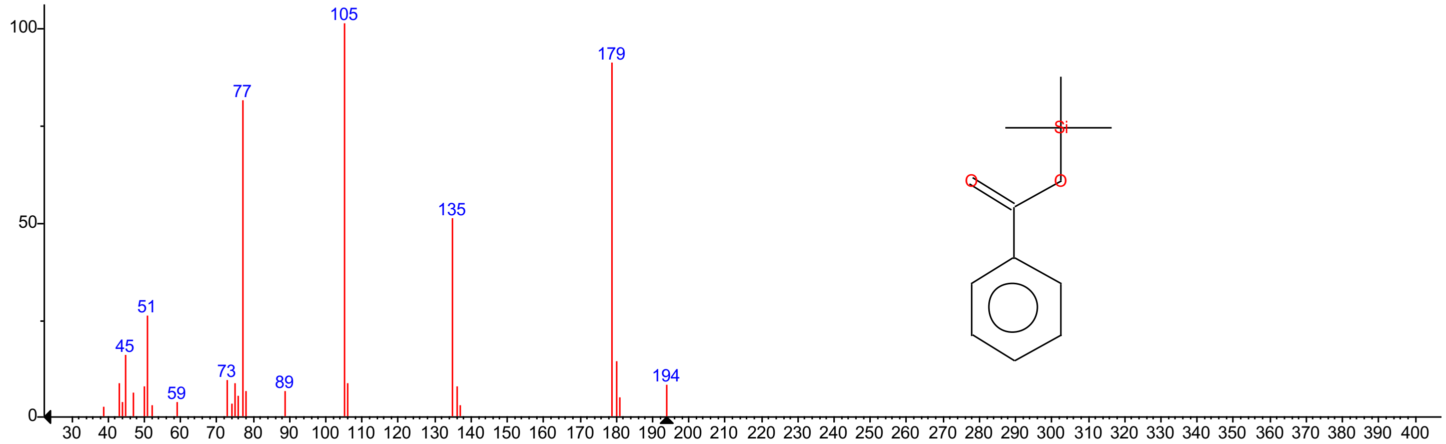

(mainlib) Benzoic acid trimethylsilyl ester

3.-Glycerol

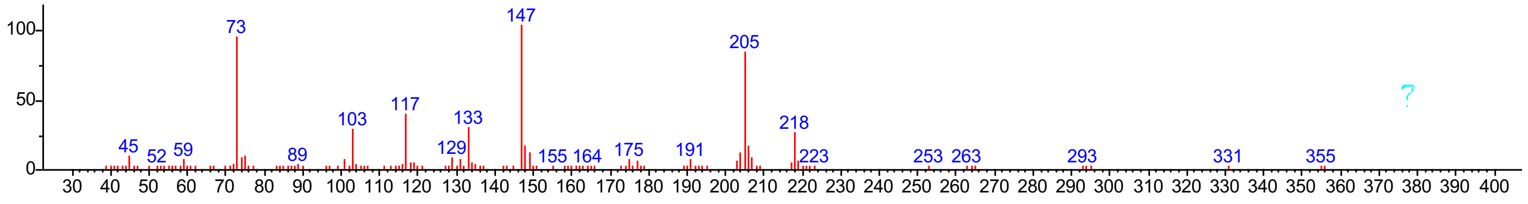

(Text File) Scan 1087 (7.061 min): DERIVATIZADAS-007.D\ data.ms (-1098)

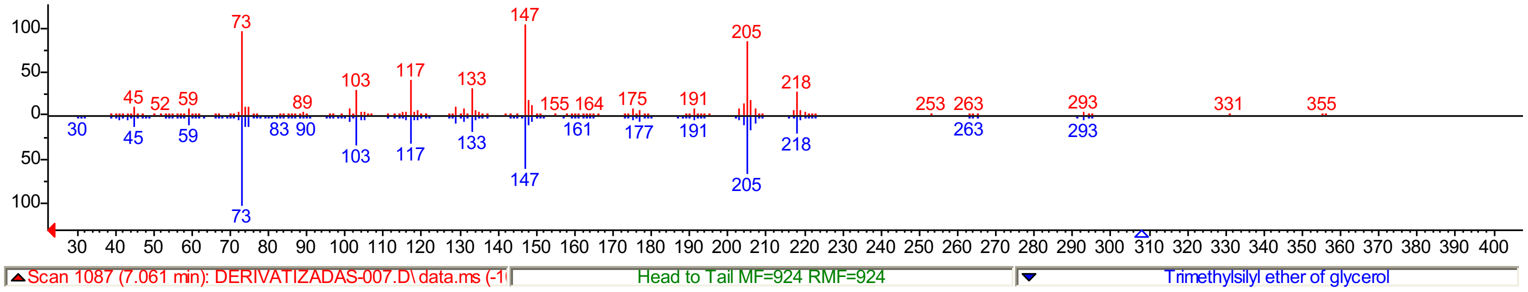

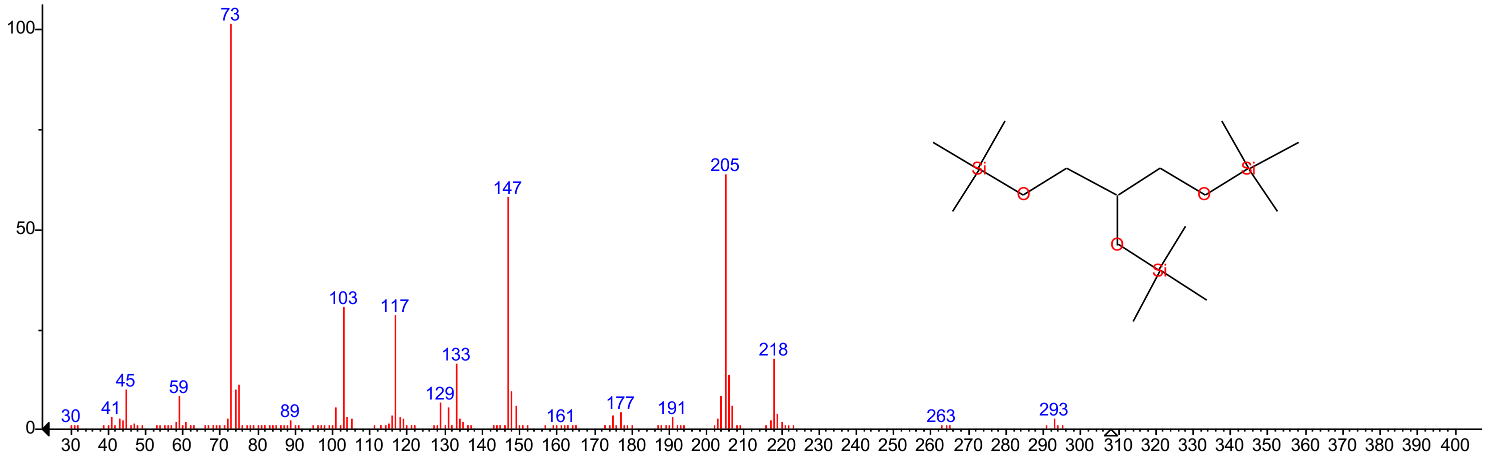

(mainlib) Trimethylsilyl ether of glycerol

4.-Cinnamic alcohol

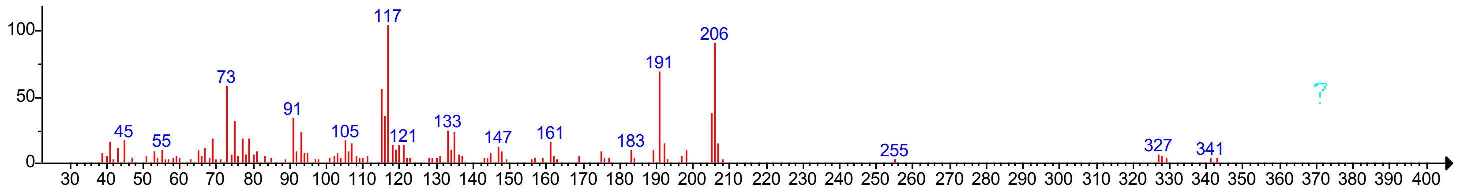

(Text File) Scan 1573 (10.179 min): DERIVATIZADAS-007.D\ data.ms (-1583)

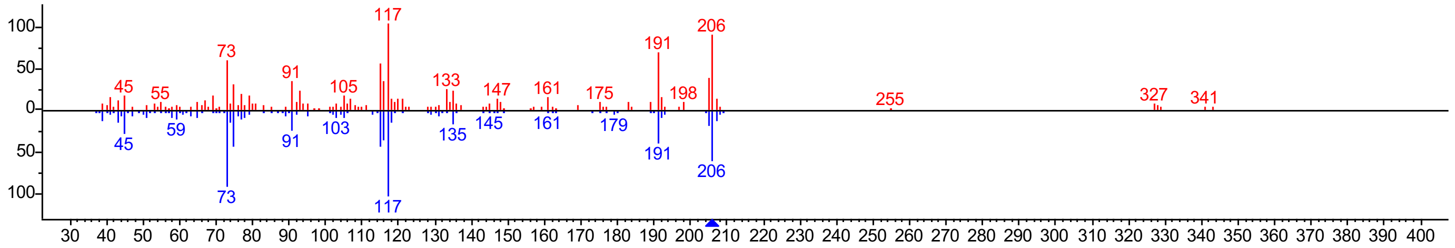

Scan 1573 (10.179 min): DERIVATIZADAS-007.D\ data.ms (- | Head to Tail MF=680 RMF=792 | 1-Phenyl-3-trimethylsilyloxy-1-propene

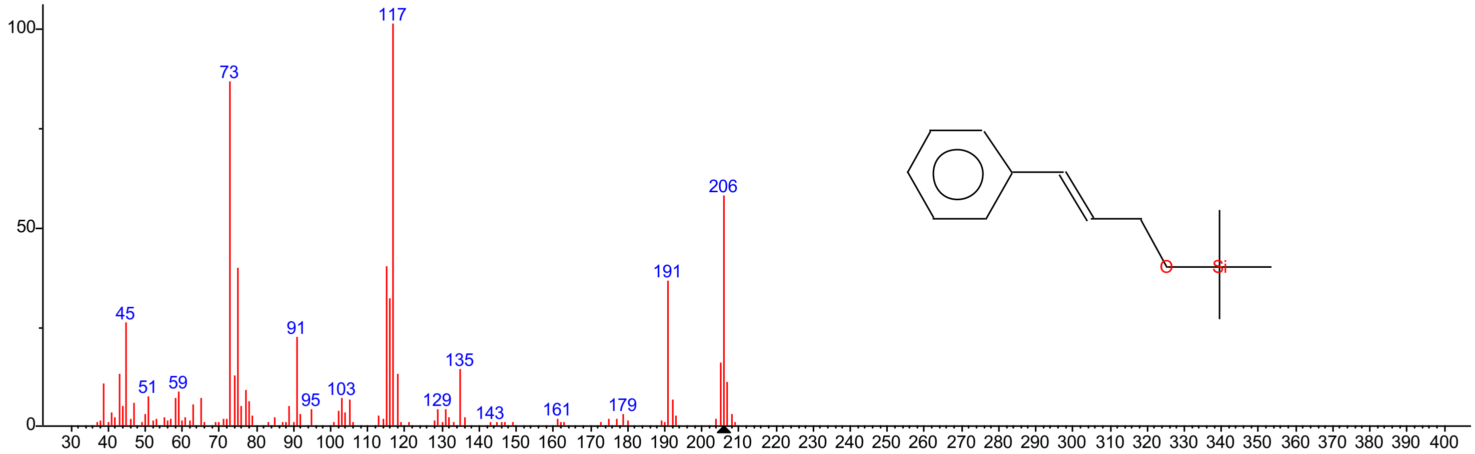

(mainlib) 1-Phenyl-3-trimethylsilyloxy-1-propene

5.- Capric acid

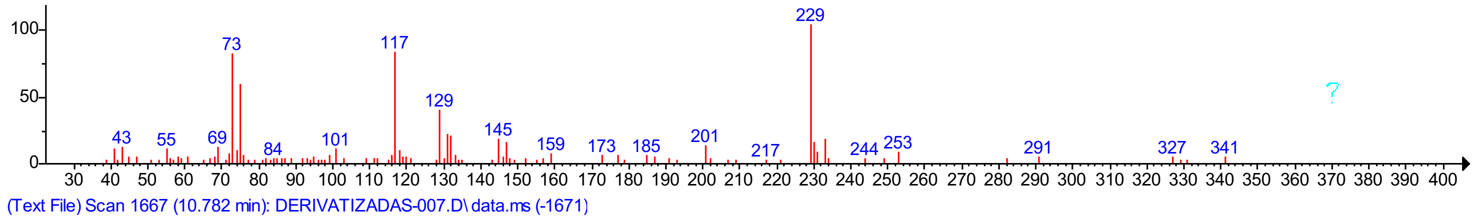

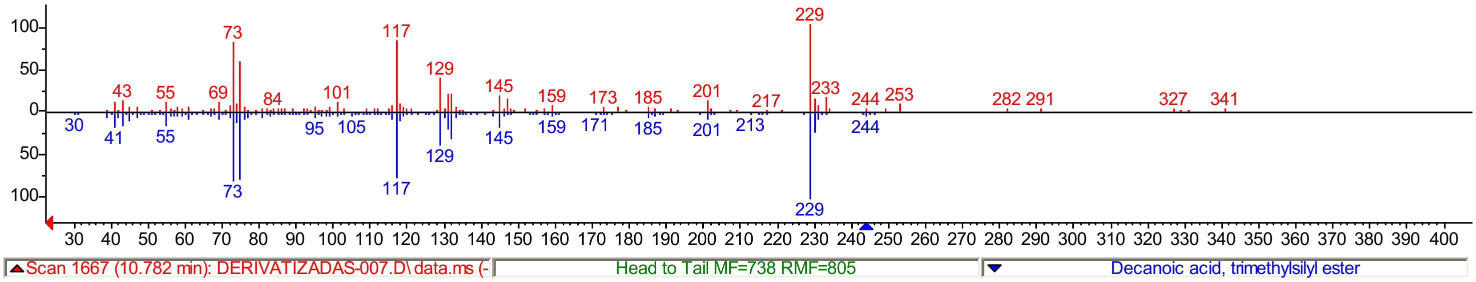

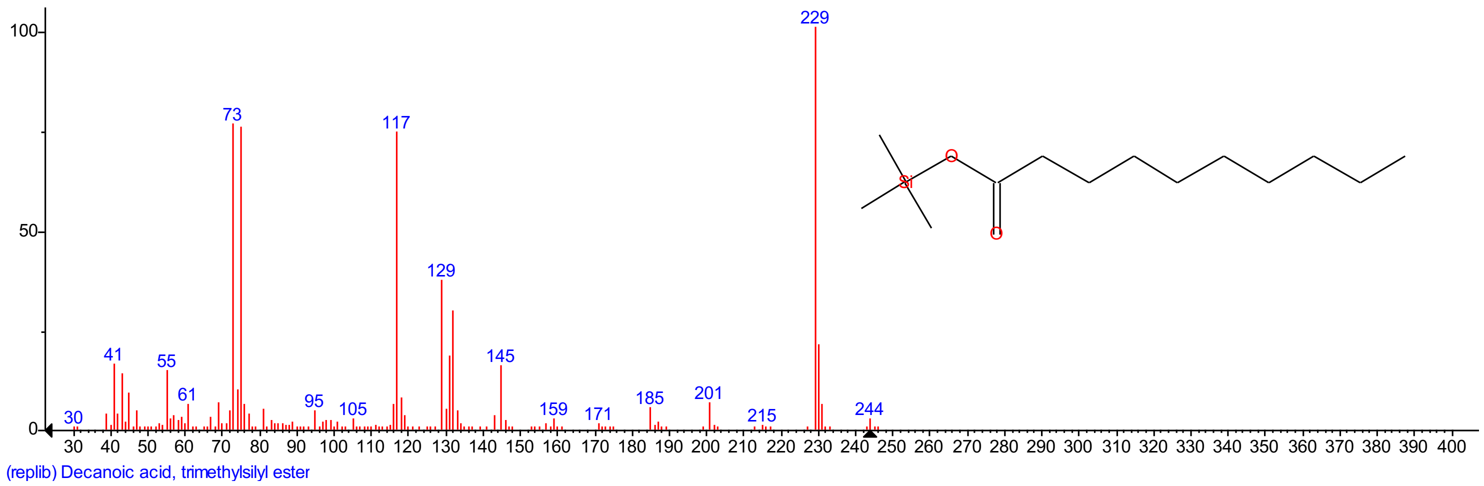

6.-4-Hydroxybenzyl alcohol

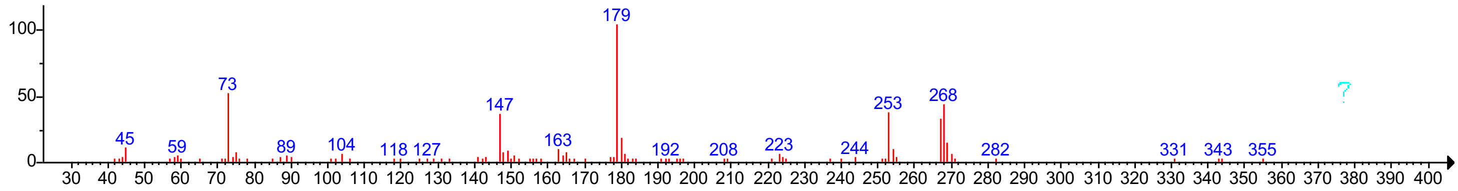

(Text File) Scan 1845 (11.924 min): DERIVATIZADAS-007.D\ data.ms (-1849)

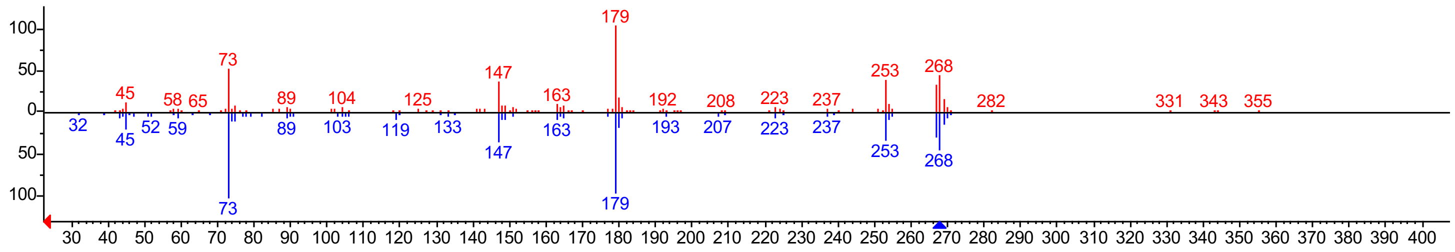

Scan 1845 (11.924 min): DERIVATIZADAS-007.D\ data.ms (- | Head to Tail MF=839 RMF=922 | Silane, trimethyl[[p-(trimethylsiloxy)benzyl]oxy]-

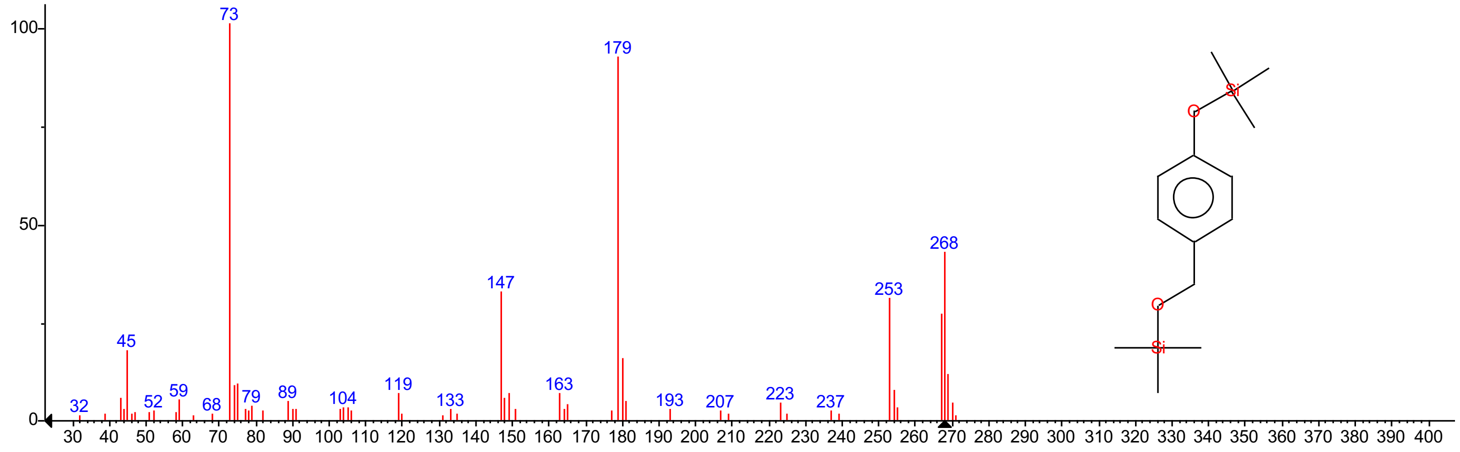

(mainlib) Silane, trimethyl[[p-(trimethylsiloxy)benzyl]oxy]-

7.-Apiol

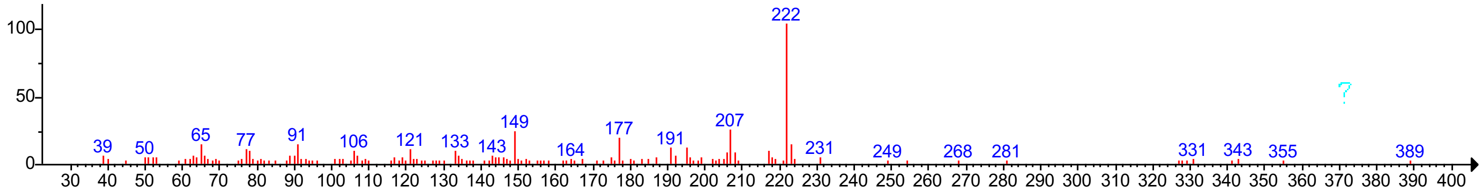

(Text File) Scan 2450 (15.805 min): DERIVATIZADAS-007.D\ data.ms (-2457)

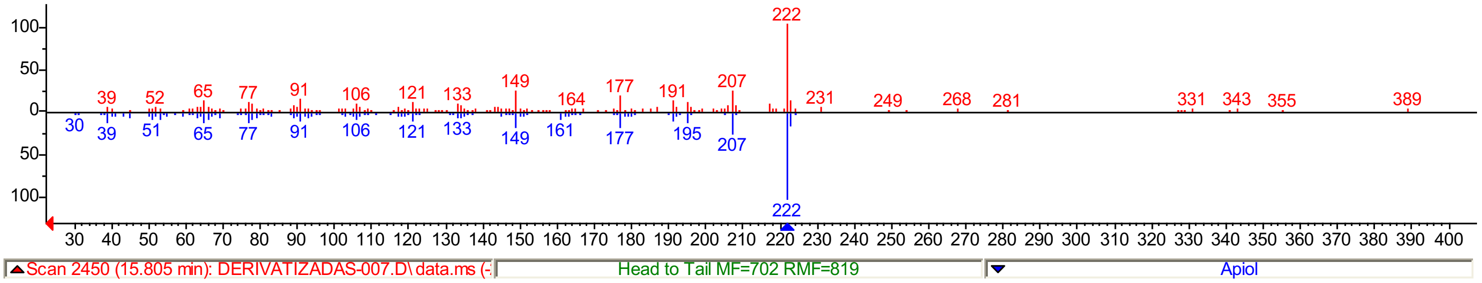

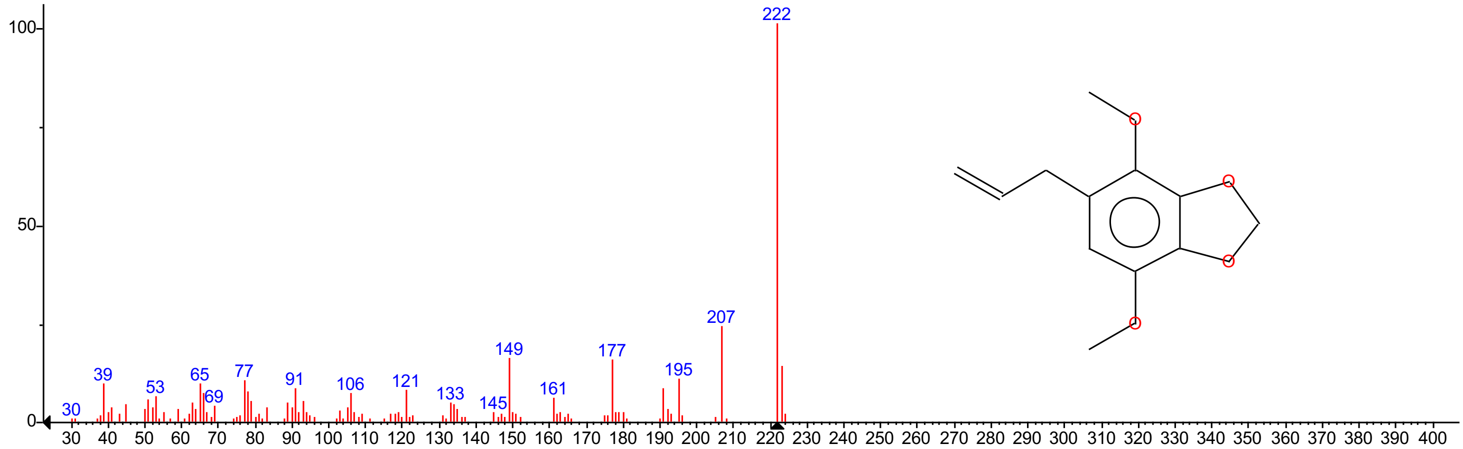

(replib) Apiol

8.-Thymol

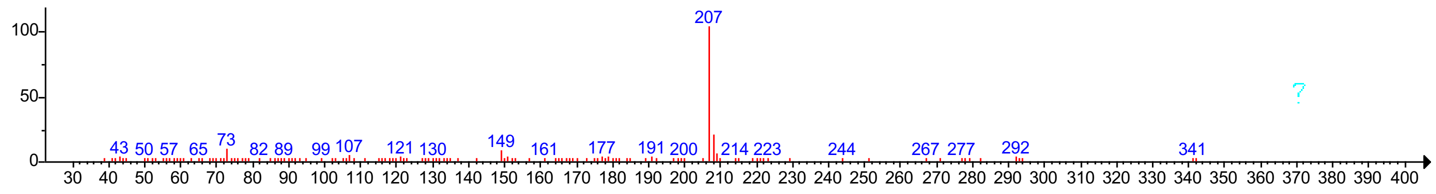

(Text File) Scan 2658 (17.139 min): DERIVATIZADAS-007.D\ data.ms (-2663)

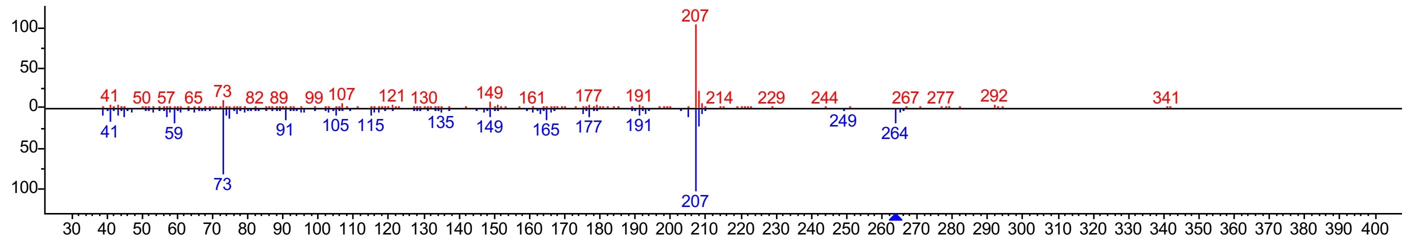

Scan 2658 (17.139 min): DERIVATIZADAS-007.D\ data.ms (- | Head to Tail MF=643 RMF=677 | Benzene, 2-[(tert-butyldimethylsilyl)oxy]-1-isopropyl-4-methyl-

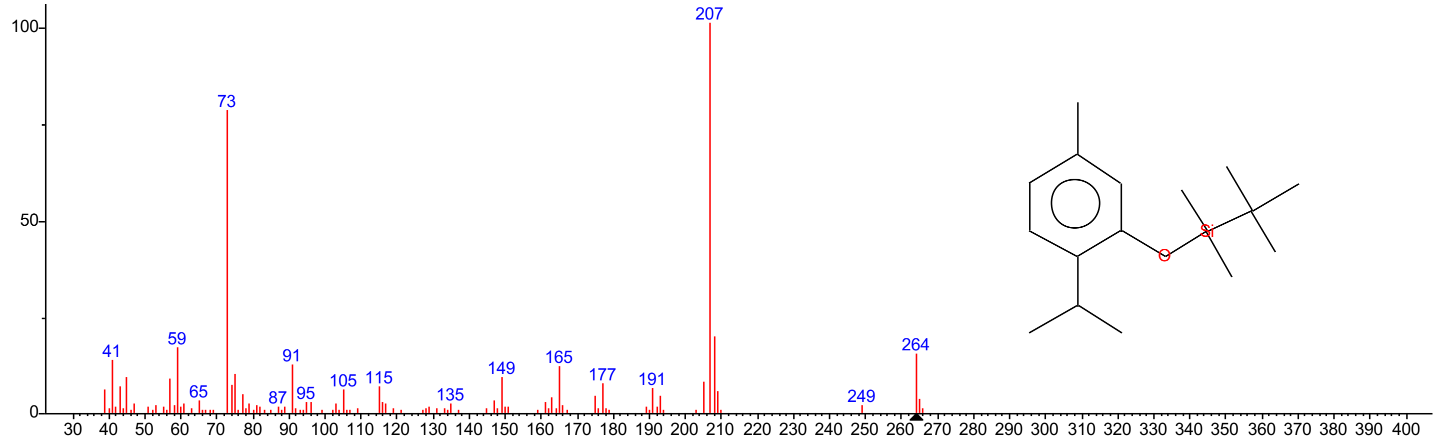

(mainlib) Benzene, 2-[(tert-butyldimethylsilyl)oxy]-1-isopropyl-4-methyl-

9.- Myristic acid

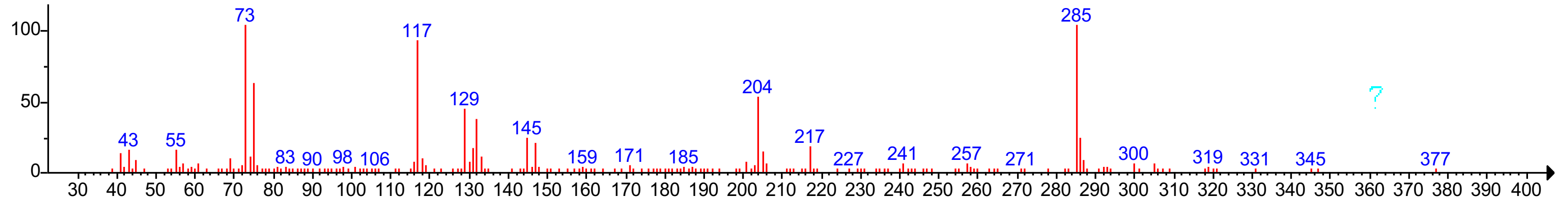

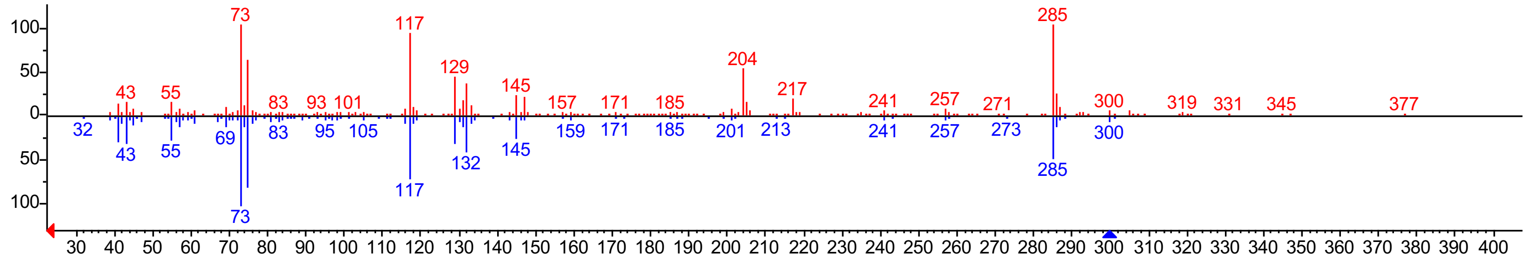

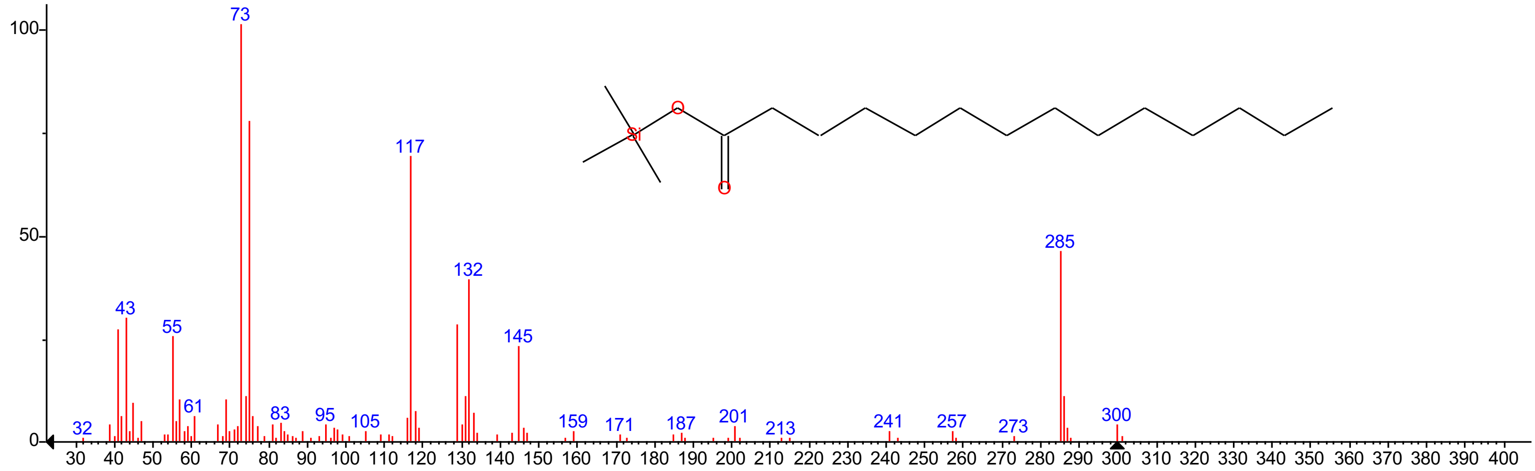

10.-*α*-D-Xylopyranose

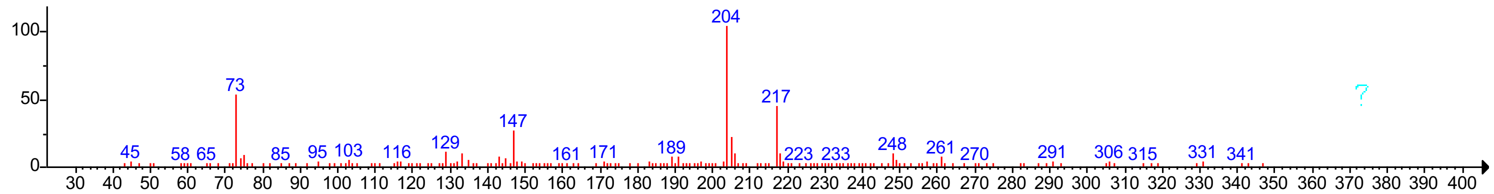

(Text File) Scan 3074 (19.808 min): DERIVATIZADAS-007.D\ data.ms (-3081)

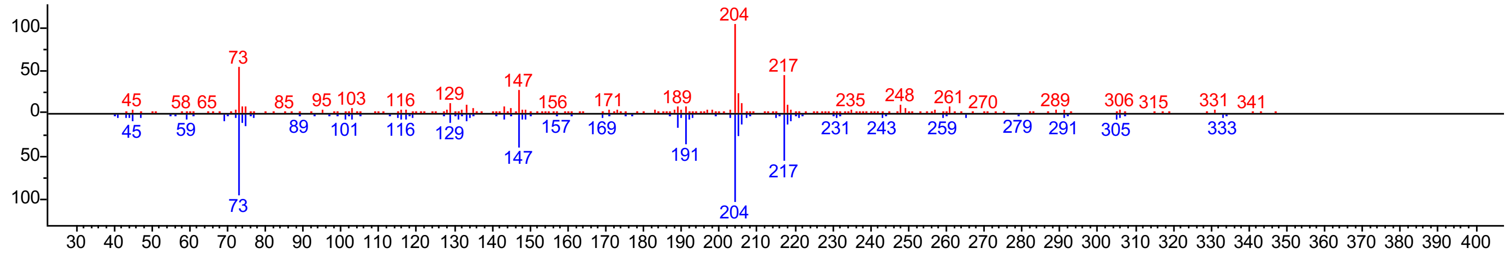

Scan 3074 (19.808 min): DERIVATIZADAS-007.D\ data.ms (- | Head to Tail MF=675 RMF=848 | a-D-Xylopyranose, 1,2,3,4-tetrakis-O-(trimethylsilyl)-

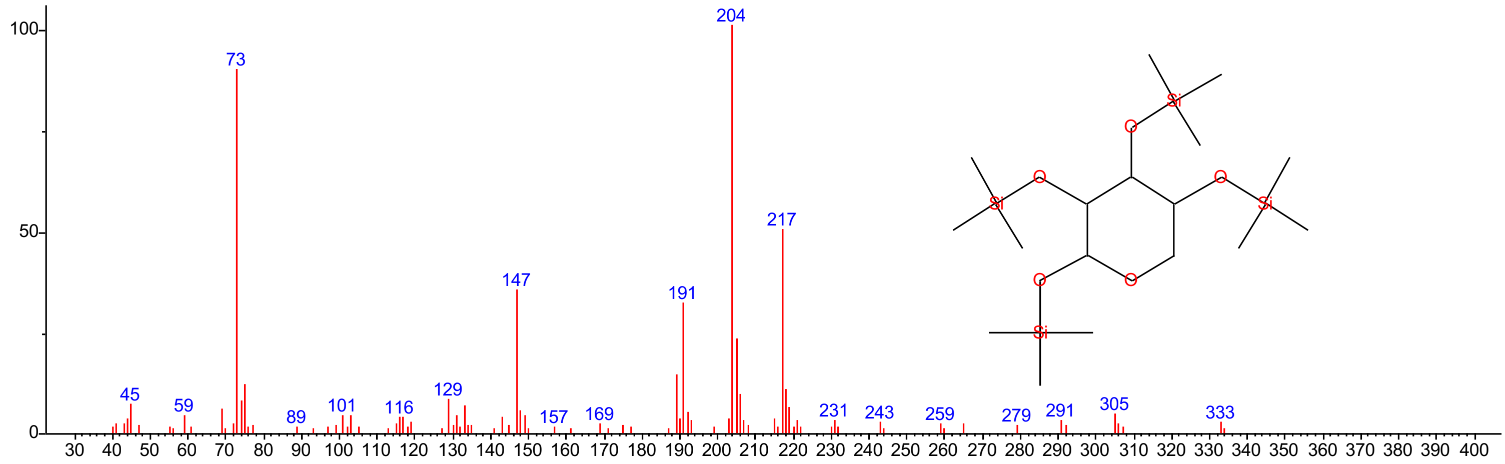

(mainlib) a-D-Xylopyranose, 1,2,3,4-tetrakis-O-(trimethylsilyl)-

11.- Palmitic acid ethyl ester

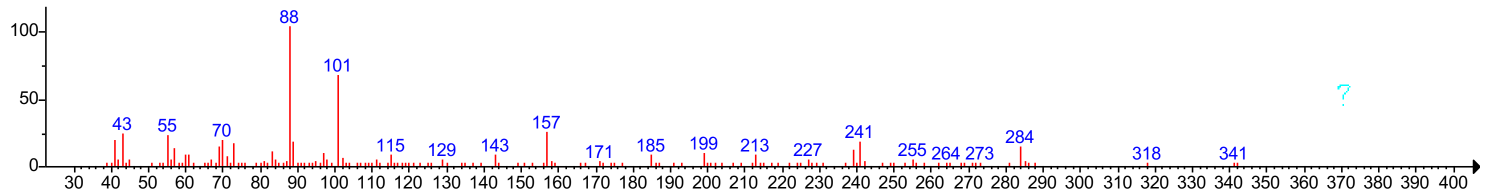

(Text File) Scan 3410 (21.964 min): DERIVATIZADAS-007.D\ data.ms (-3419)

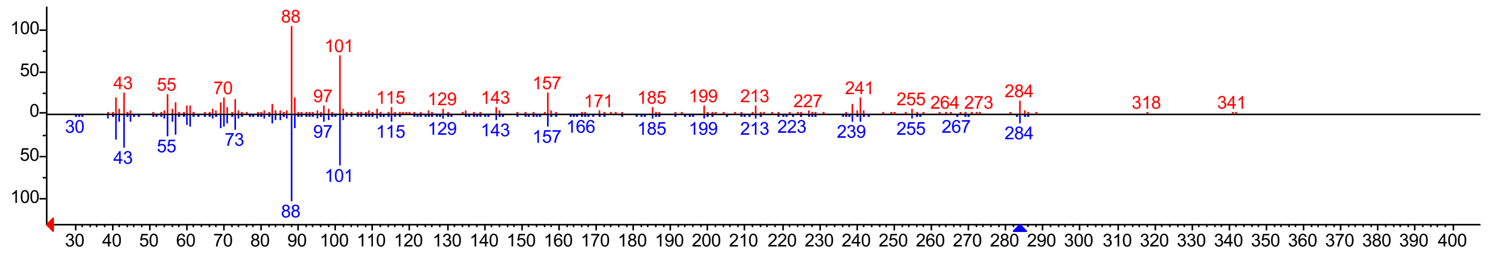

Scan 3410 (21.964 min): DERIVATIZADAS-007.D\ data.ms (-3419) | Head to Tail MF=874 RMF=889 | Hexadecanoic acid, ethyl ester

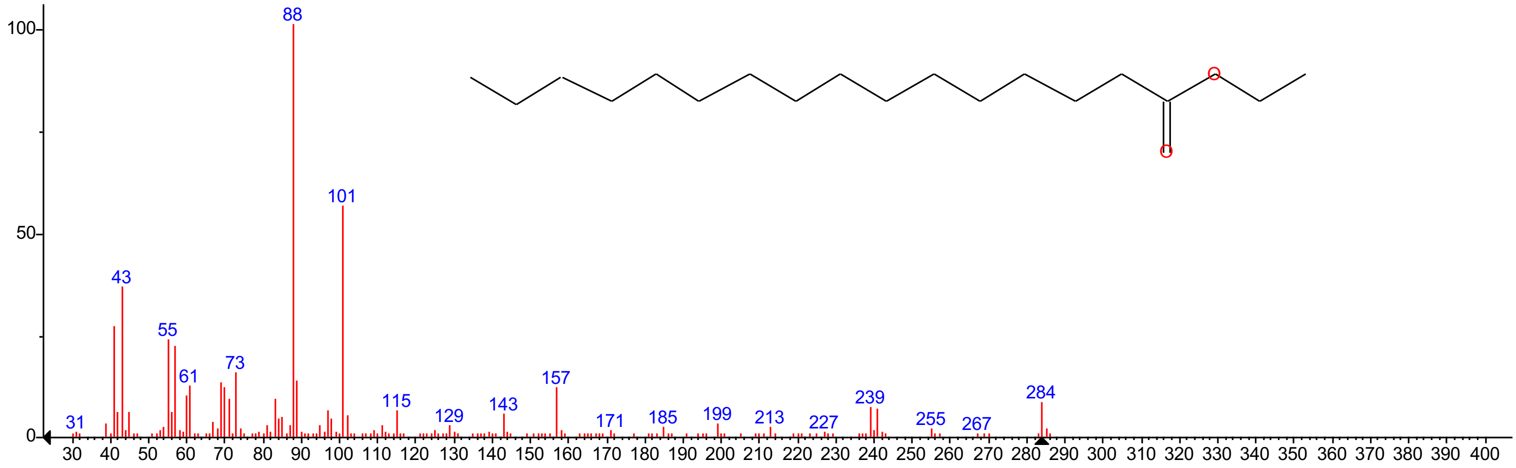

(mainlib) Hexadecanoic acid, ethyl ester

12.- Palmitic acid

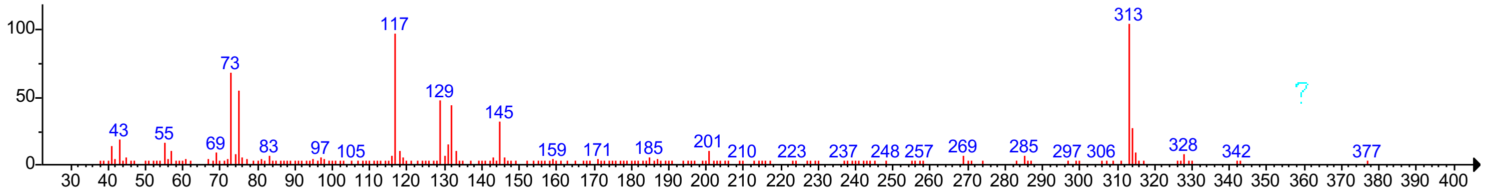

(Text File) Scan 3570 (22.990 min): DERIVATIZADAS-007.D\ data.ms (-3592)

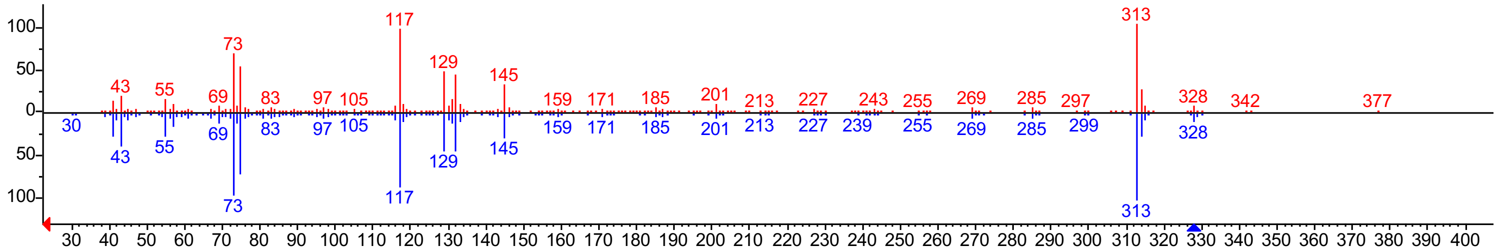

Scan 3570 (22.990 min): DERIVATIZADAS-007.D\ data.ms (- | Head to Tail MF=917 RMF=924 | Hexadecanoic acid, trimethylsilyl ester

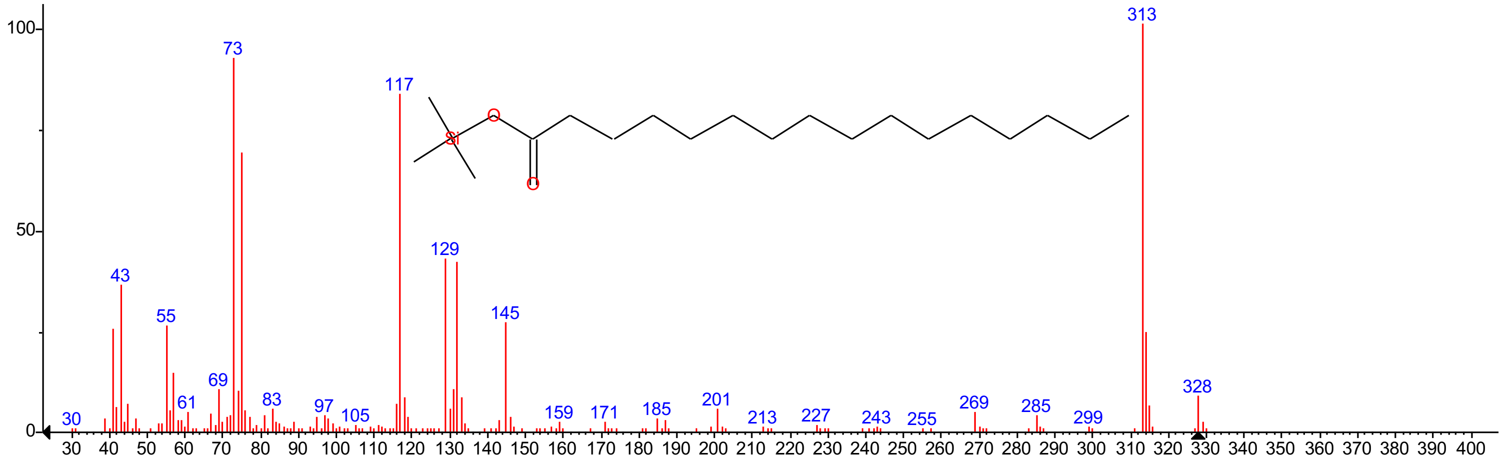

(replib) Hexadecanoic acid, trimethylsilyl ester

13.- Heneicosane

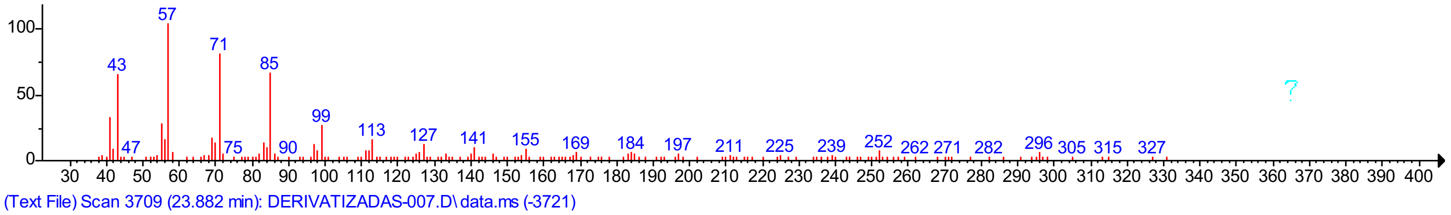

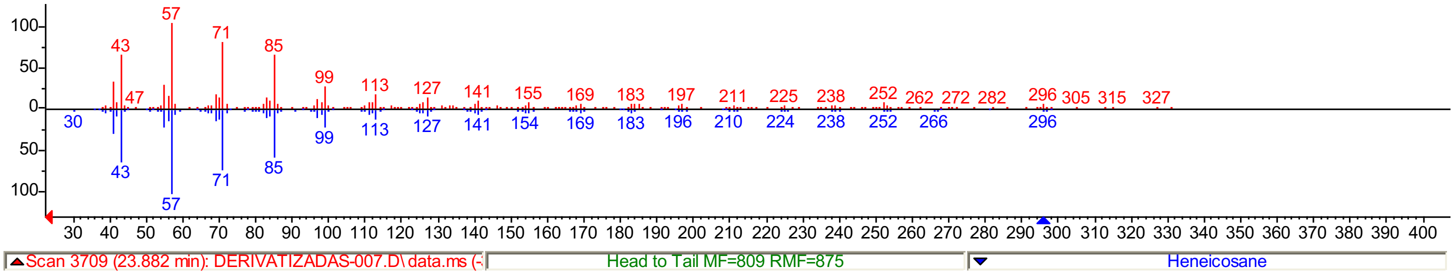

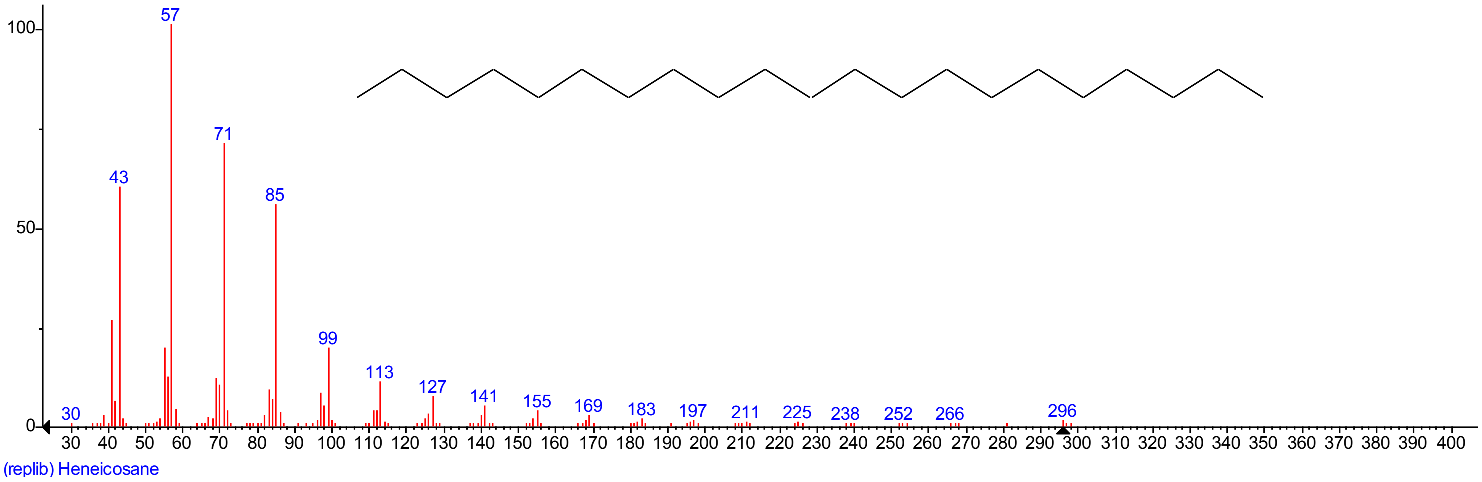

14.-Oleic acid

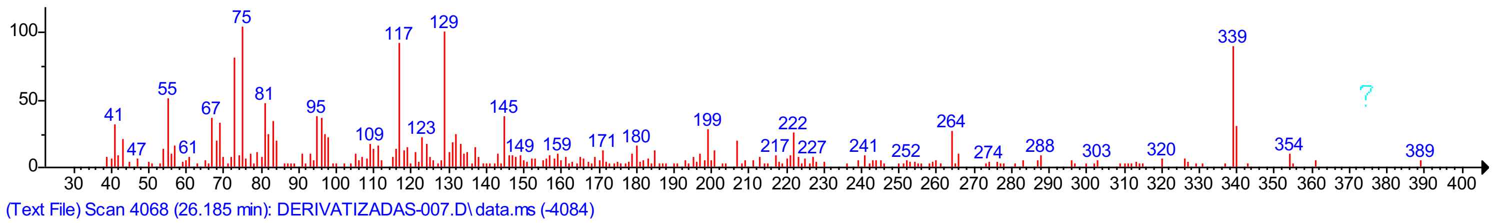

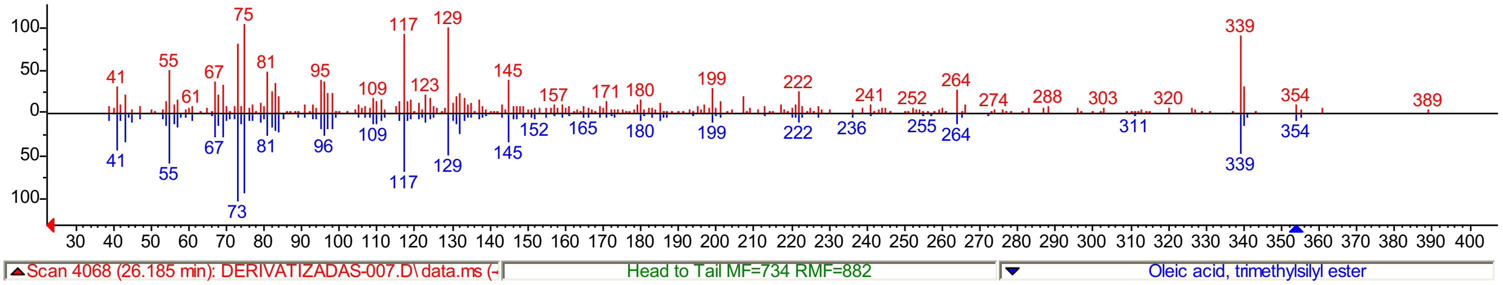

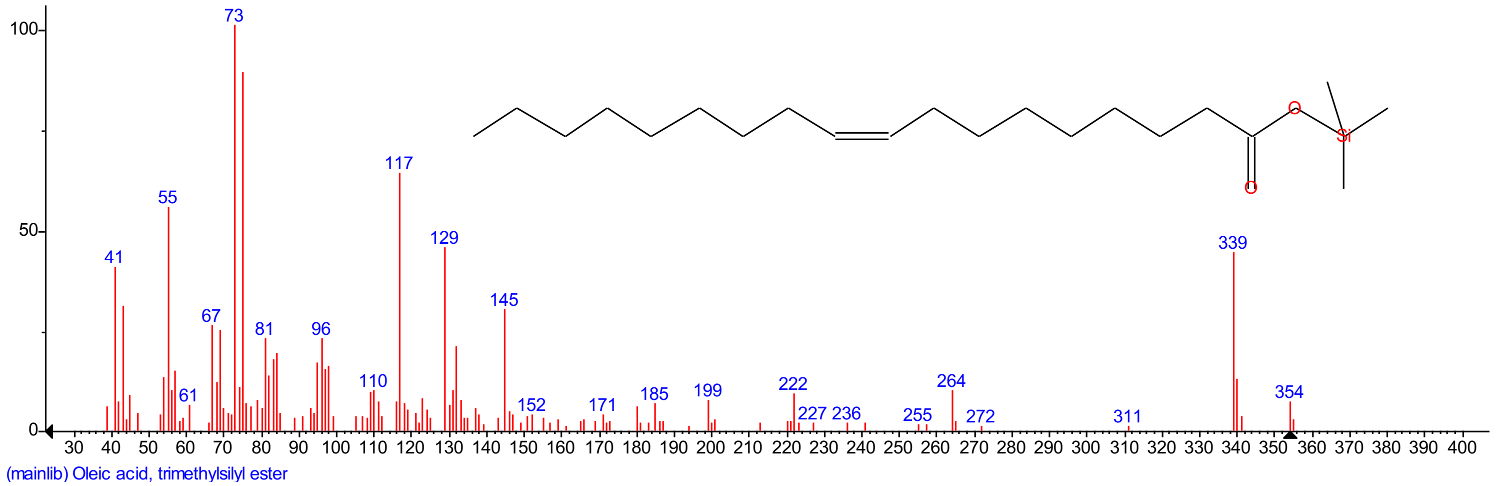

15.- Stearic acid

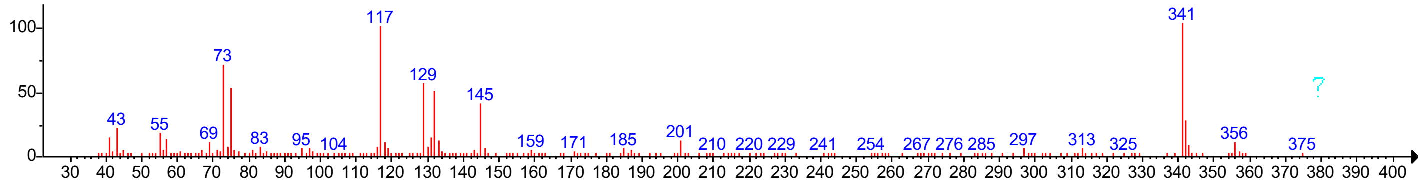

(Text File) Scan 4120 (26.518 min): DERIVATIZADAS-007.D\ data.ms (-4139)

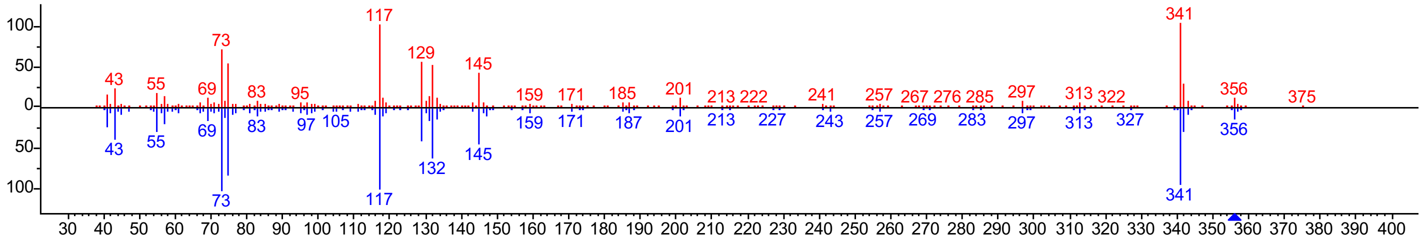

Scan 4120 (26.518 min): DERIVATIZADAS-007.D\ data.ms (- | Head to Tail MF=918 RMF=939 | Octadecanoic acid, trimethylsilyl ester

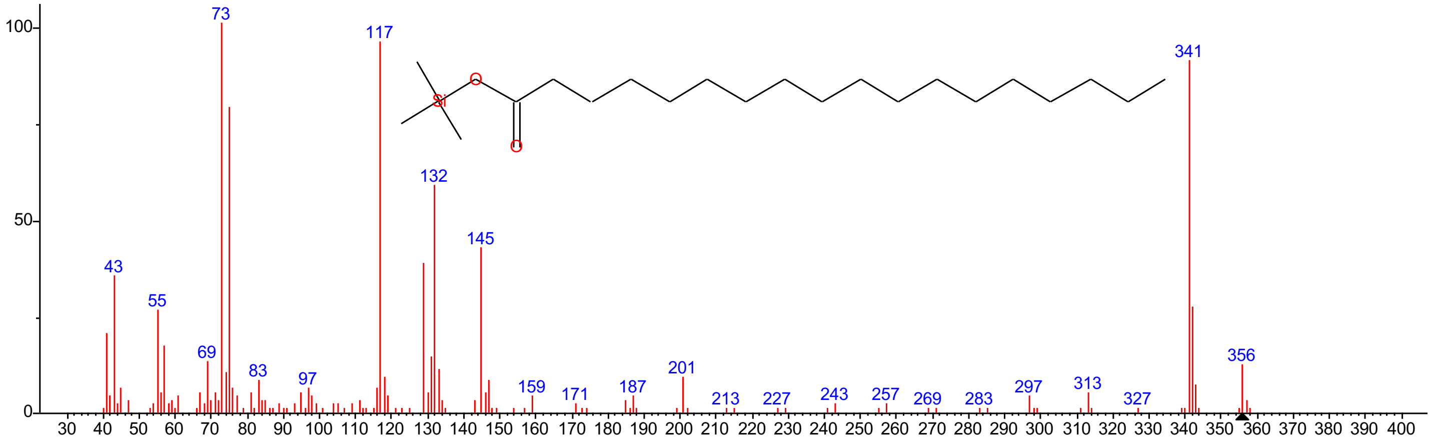

(replib) Octadecanoic acid, trimethylsilyl ester

16.-Octacosane

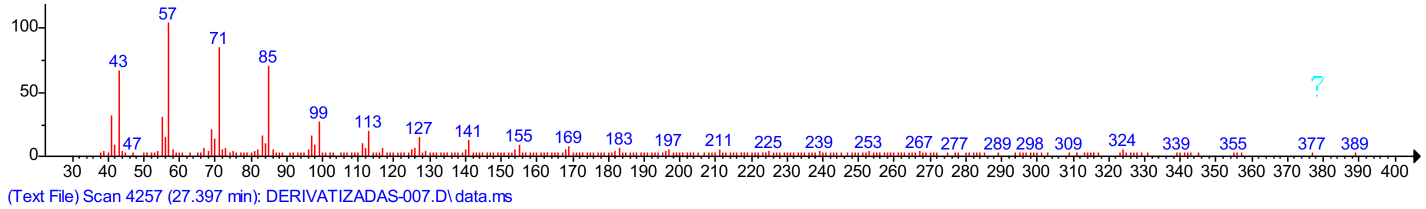

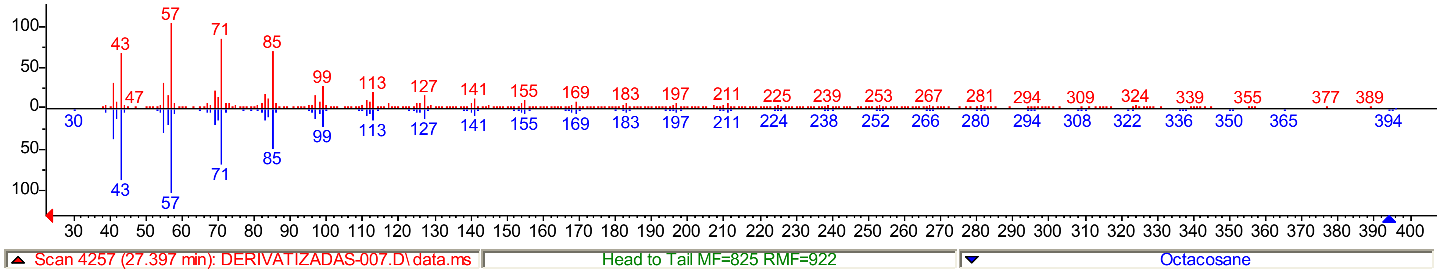

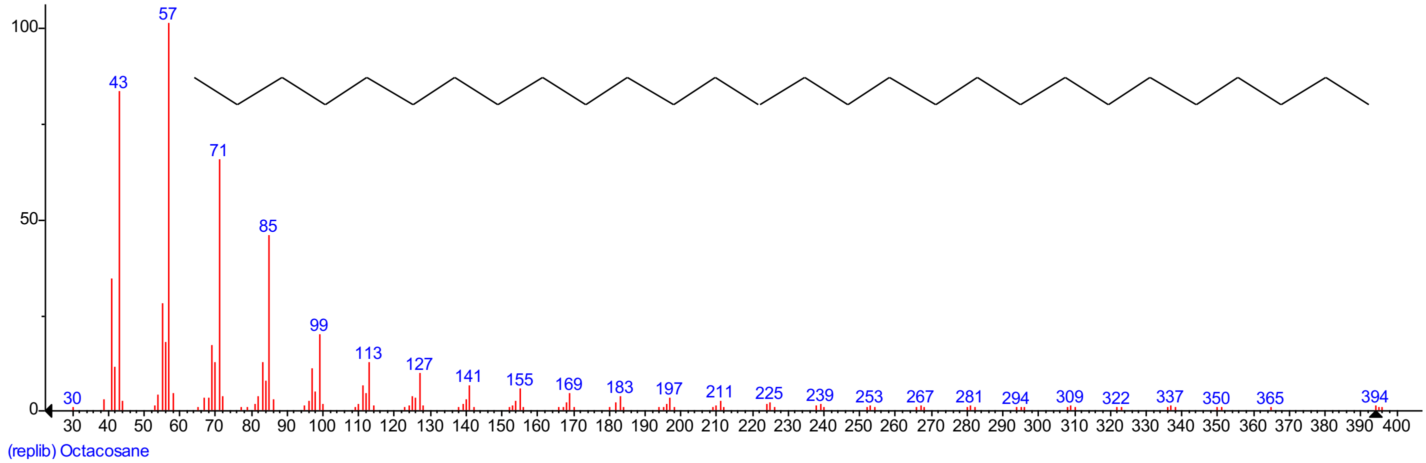

17.-Hexacosane

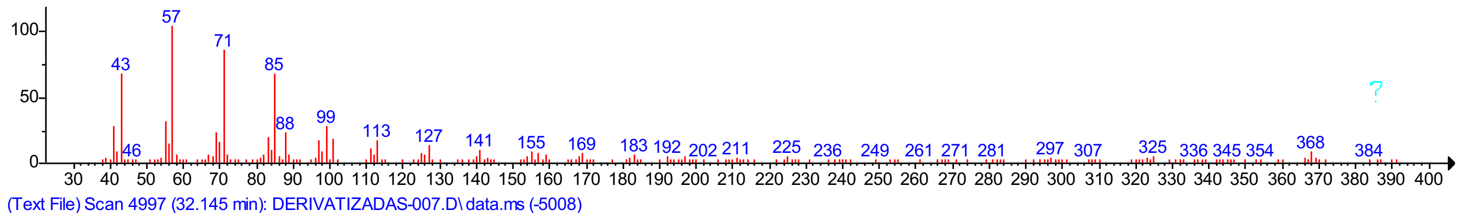

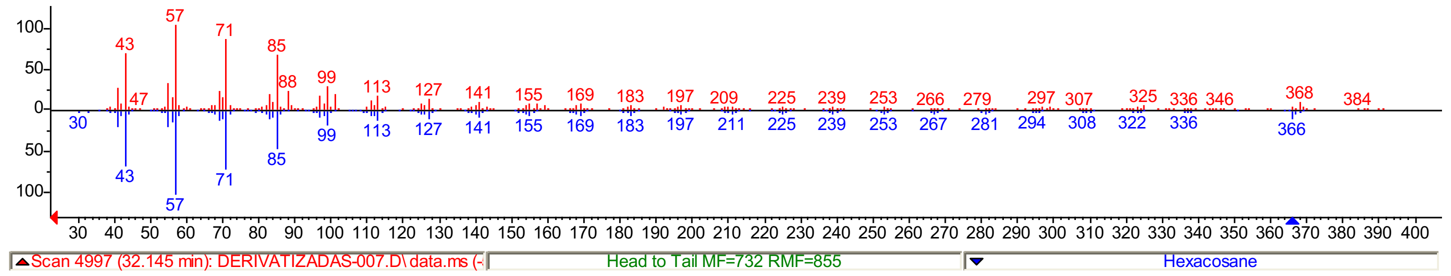

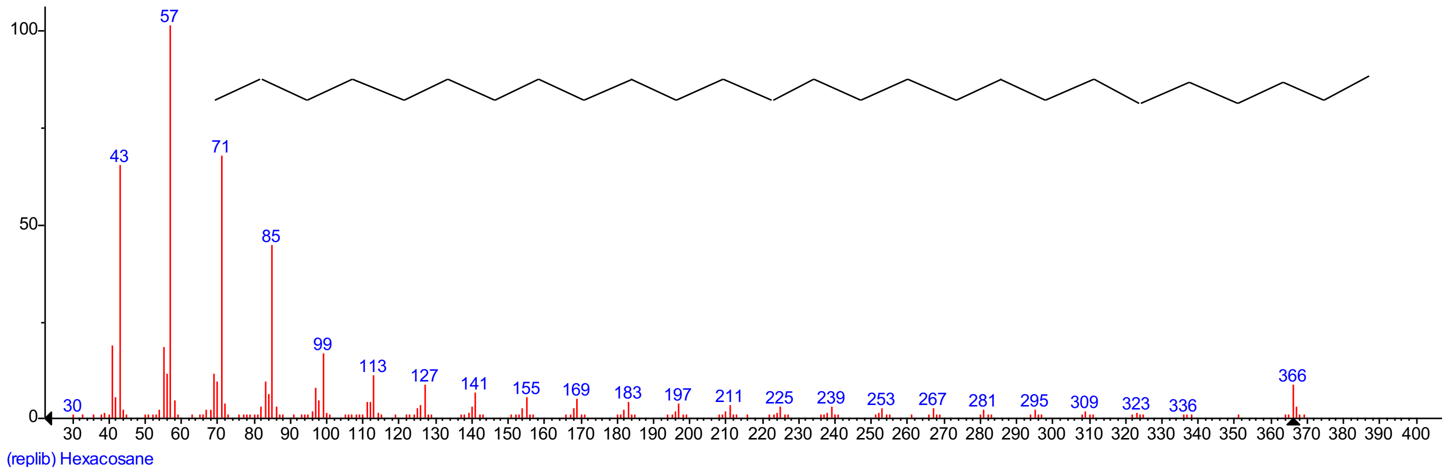

Mexicali hexanic extract of propolis

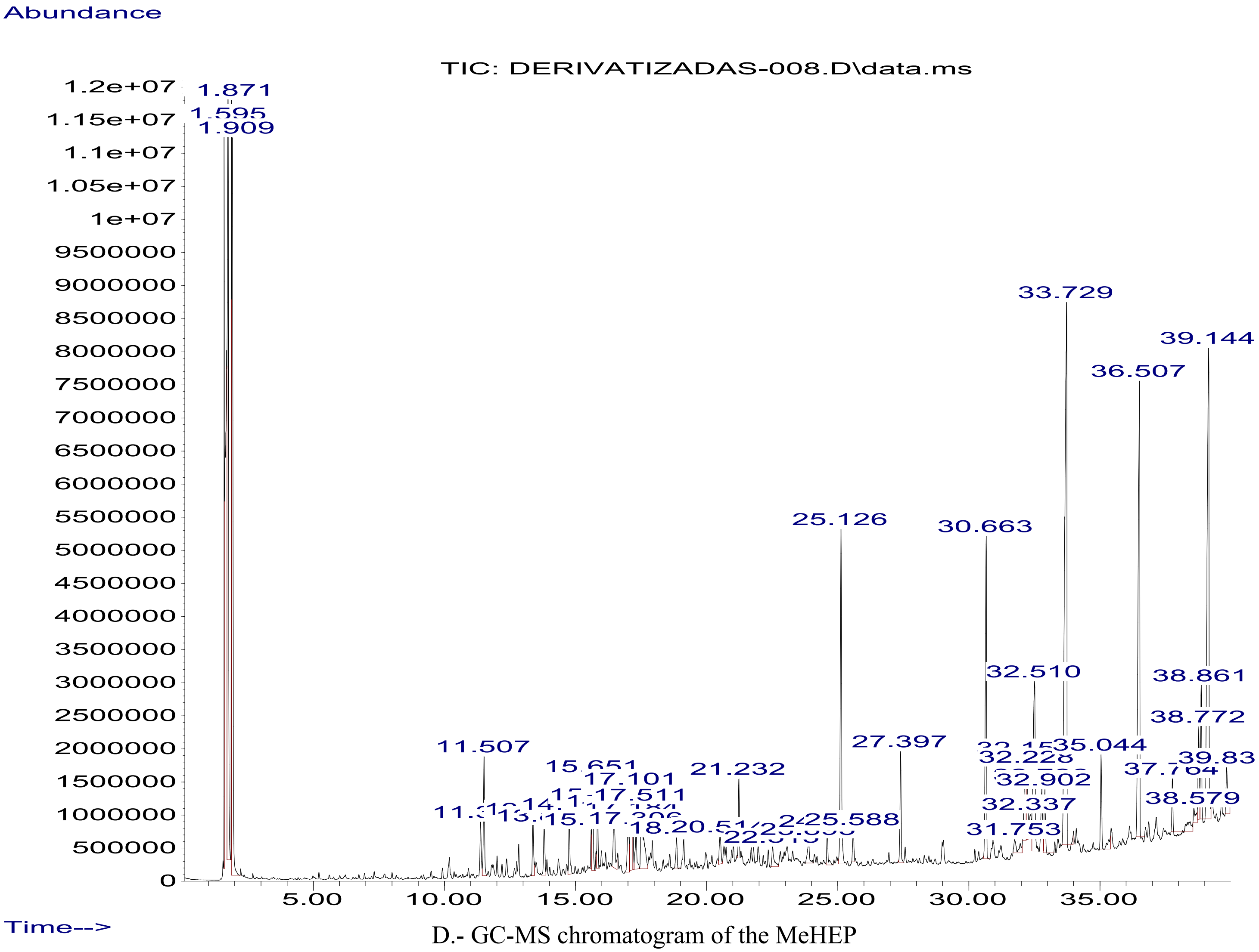

D.- GC-MS chromatogram of the MeHEP

1.-α-Pinene

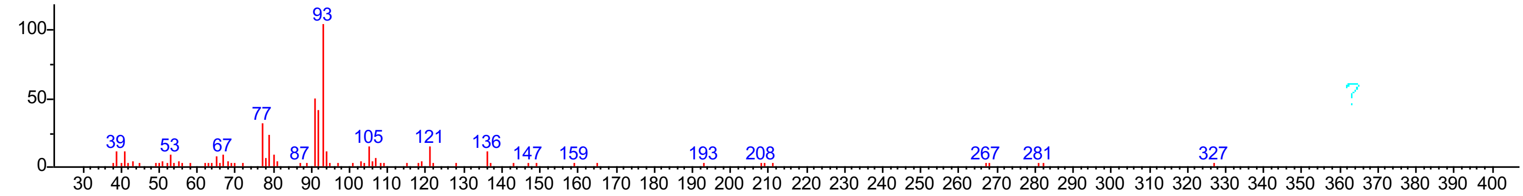

(Text File) Scan 406 (2.692 min): DERIVATIZADAS-008.D\ data.ms (-399)

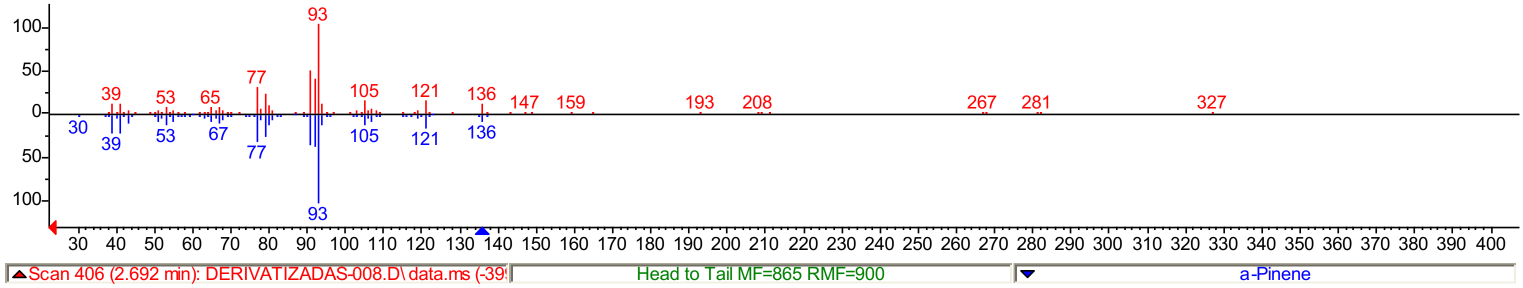

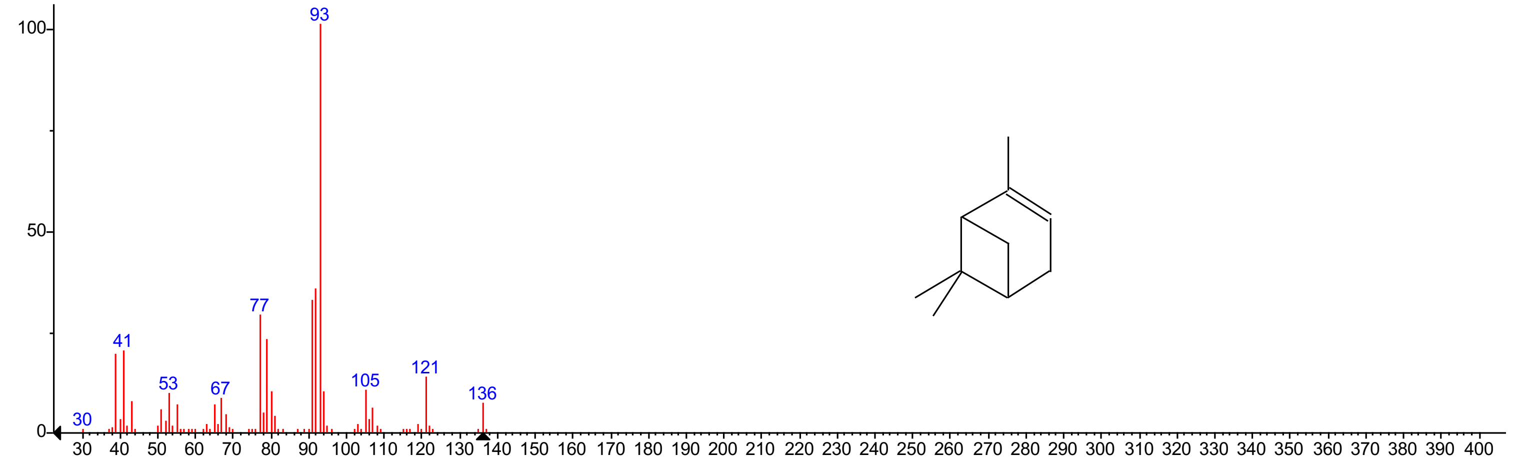

(mainlib) a-Pinene

2.- Camphor

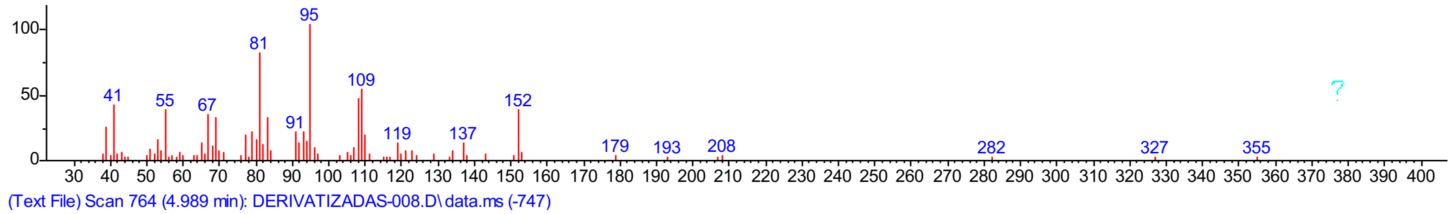

100
50
0
95
81
109
41
55
67
91
119
137
152
179
193
208
282
327
355
30 40 50 60 70 80 90 100 110 120 130 140 150 160 170 180 190 200 210 220 230 240 250 260 270 280 290 300 310 320 330 340 350 360 370 380 390 400
(Text File) Scan 764 (4.989 min): DERIVATIZADAS-008.D\ data.ms (-747)

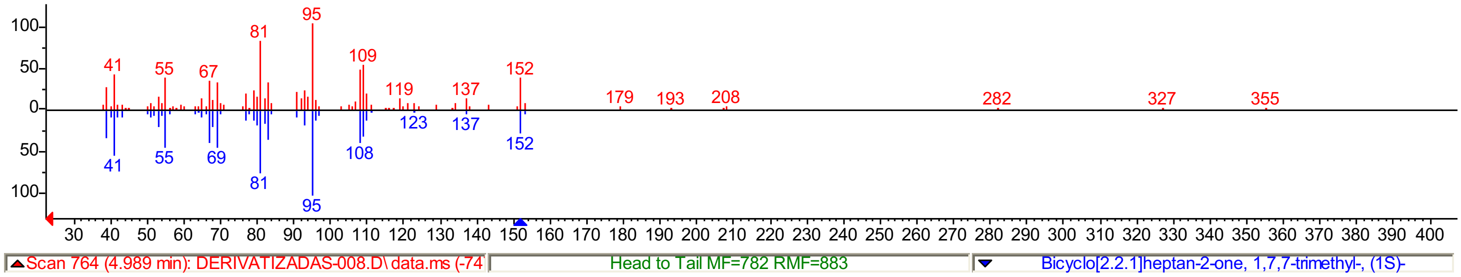

100
50
0
50
100
95
81
109
41
55
67
119
137
152
179
193
208
282
327
355
41
55
69
81
95
108
123
137
152
30 40 50 60 70 80 90 100 110 120 130 140 150 160 170 180 190 200 210 220 230 240 250 260 270 280 290 300 310 320 330 340 350 360 370 380 390 400
Scan 764 (4.989 min): DERIVATIZADAS-008.D\ data.ms (-74
Head to Tail MF=782 RMF=883
Bicyclo[2.2.1]heptan-2-one, 1,7,7-trimethyl-, (1S)-

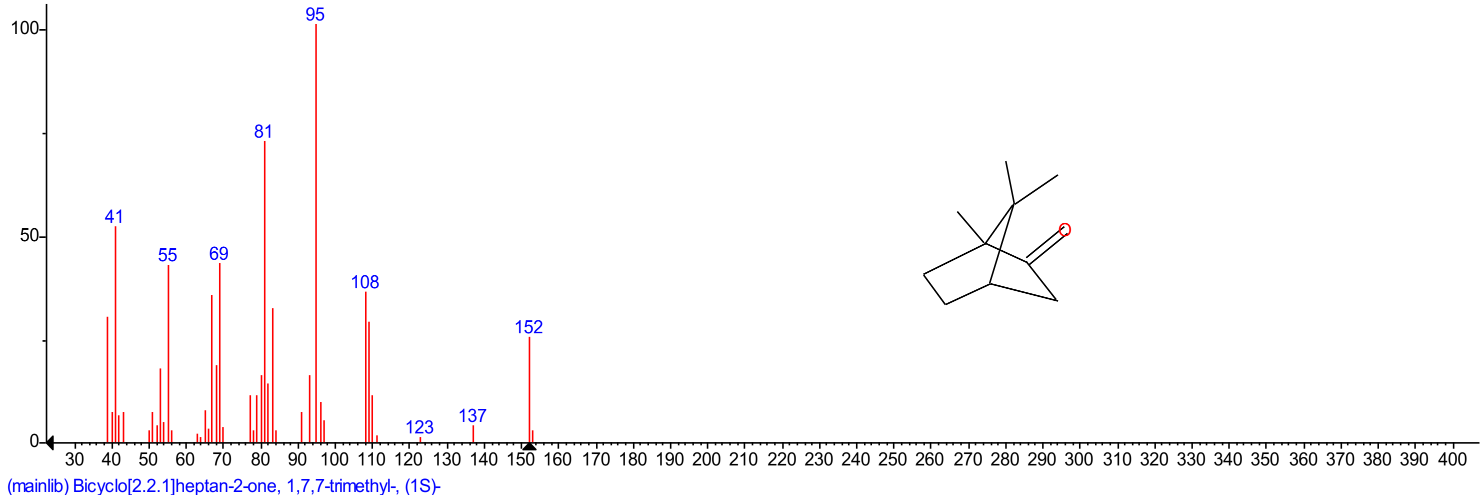

100
50
0
95
81
41
55
69
108
152
123
137
O
30 40 50 60 70 80 90 100 110 120 130 140 150 160 170 180 190 200 210 220 230 240 250 260 270 280 290 300 310 320 330 340 350 360 370 380 390 400
(mainlib) Bicyclo[2.2.1]heptan-2-one, 1,7,7-trimethyl-, (1S)-

3.-Myrtenal

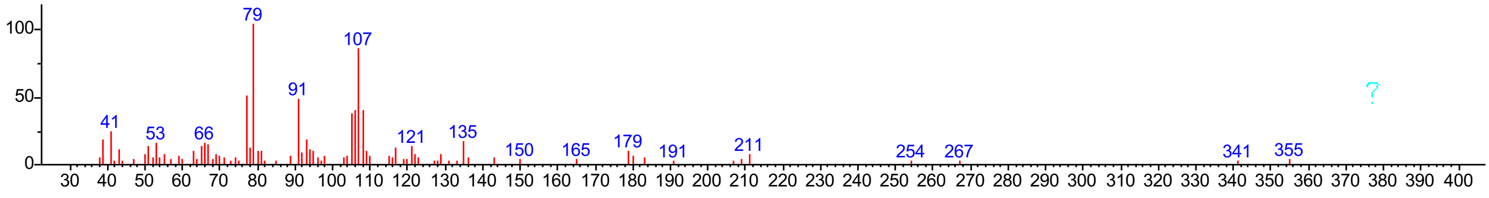

(Text File) Scan 886 (5.771 min): DERIVATIZADAS-008.D\ data.ms (-894)

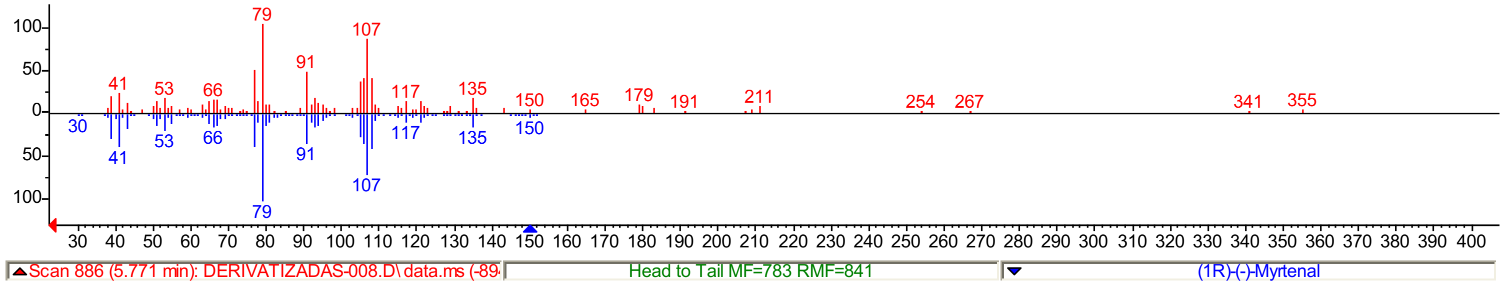

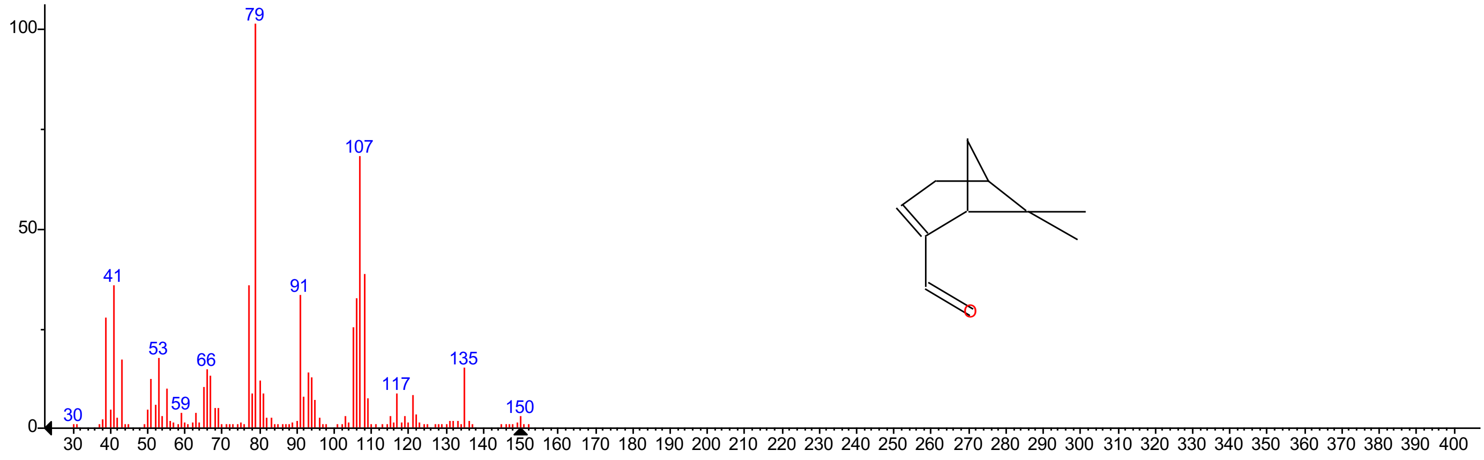

(mainlib) (1R)-(-)-Myrtenal

4.- D-Verbenone

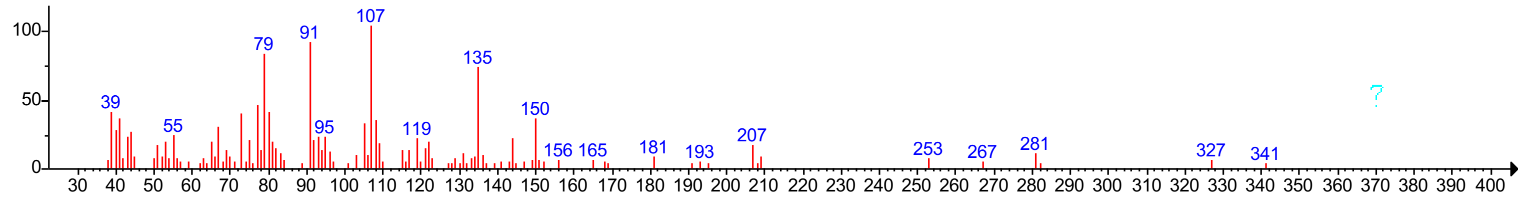

(Text File) Scan 923 (6.009 min): DERIVATIZADAS-008.D\ data.ms

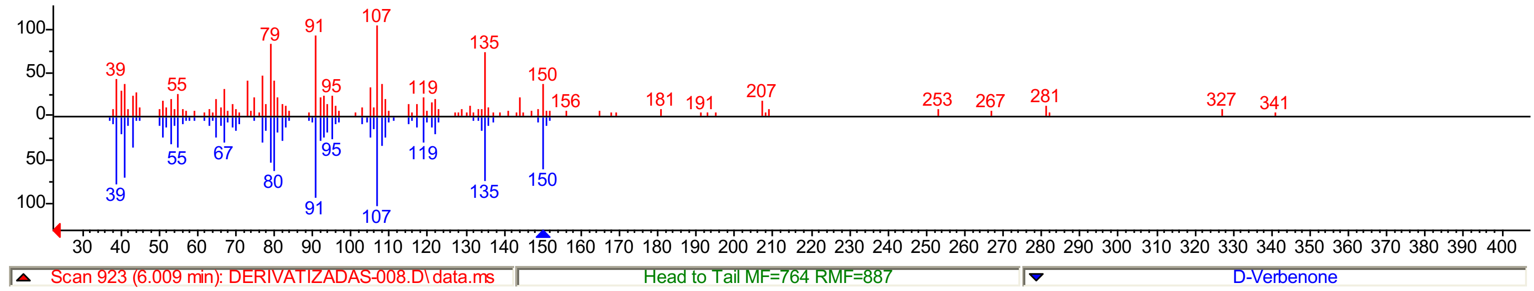

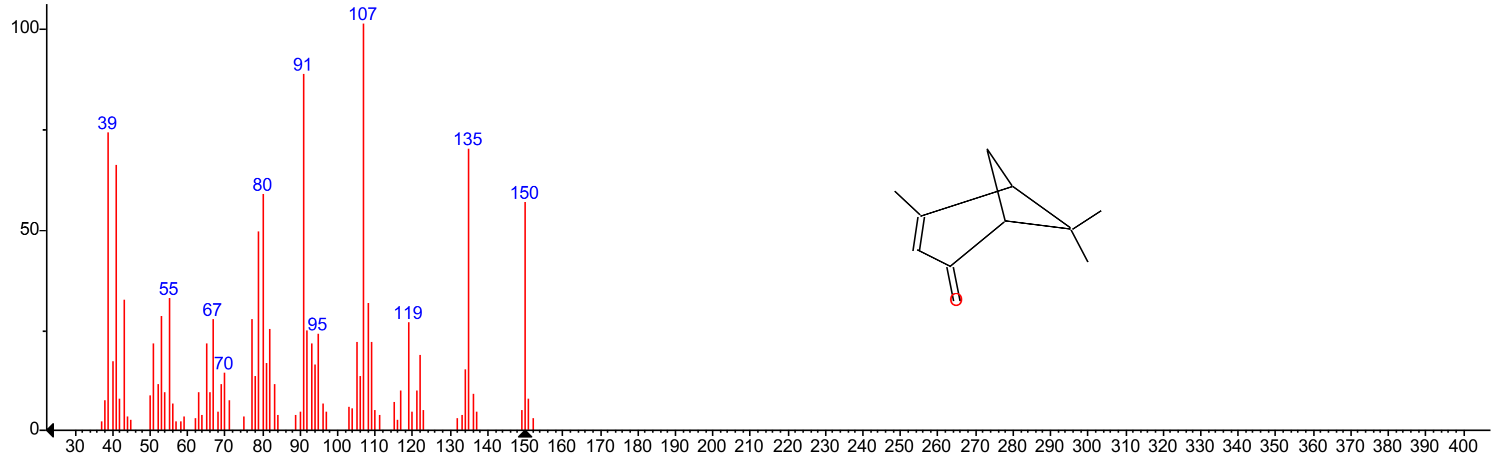

(mainlib) D-Verbenone

5.- Cuminaldehyde

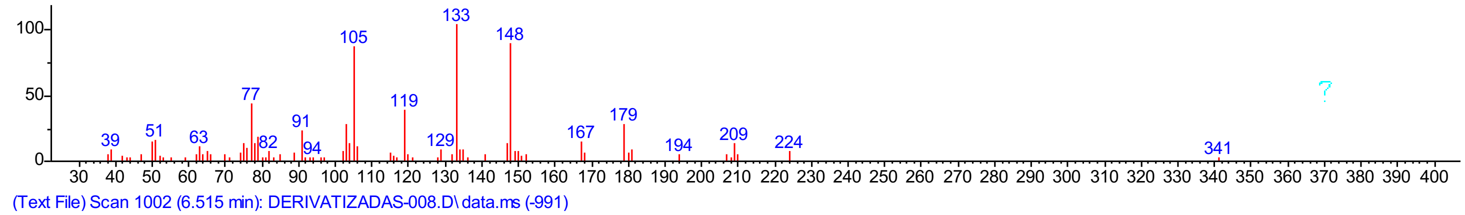

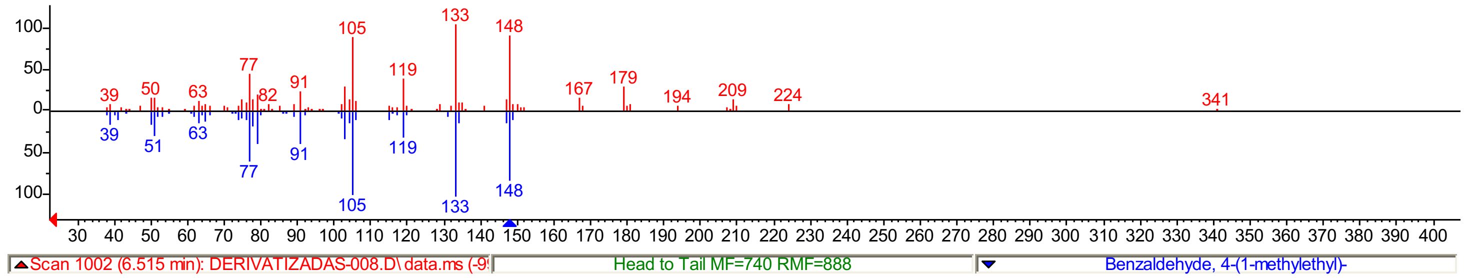

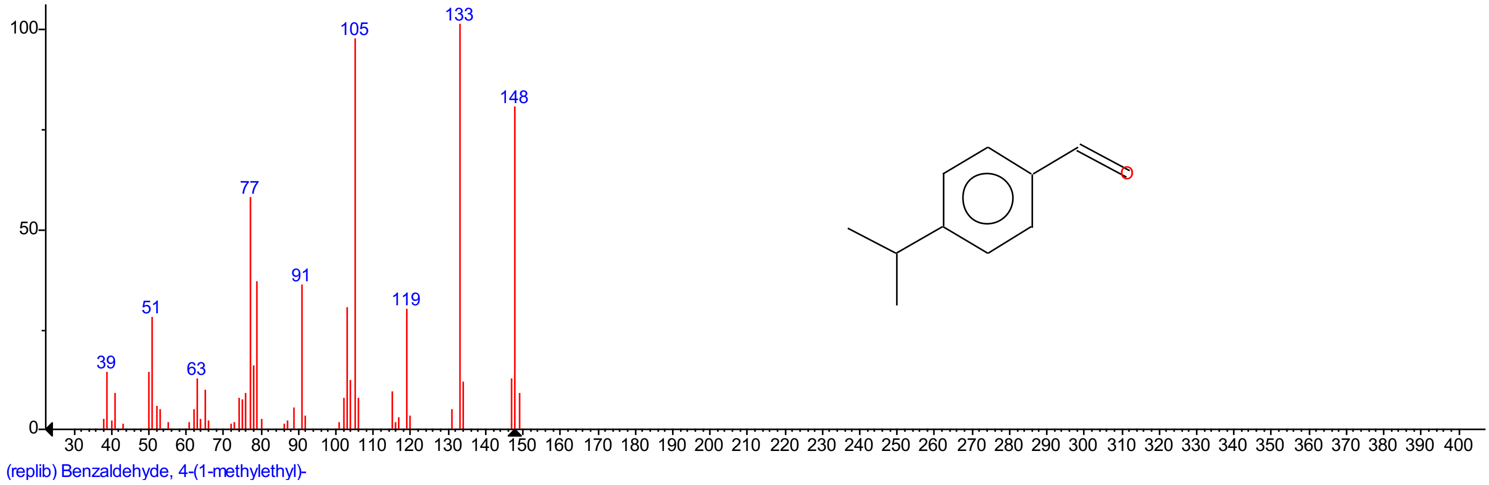

6.- Bornyl acetate

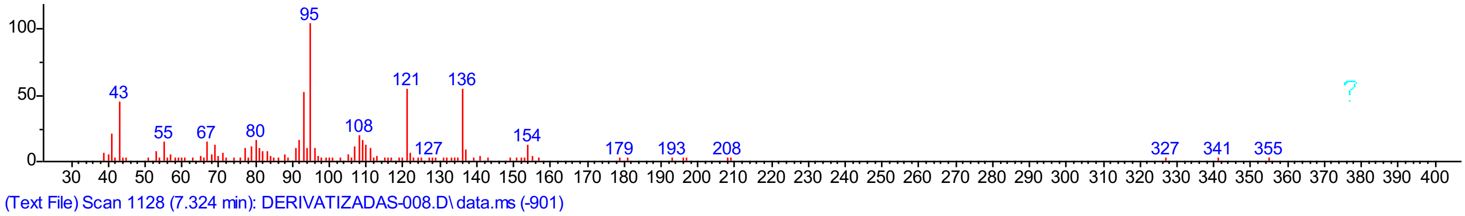

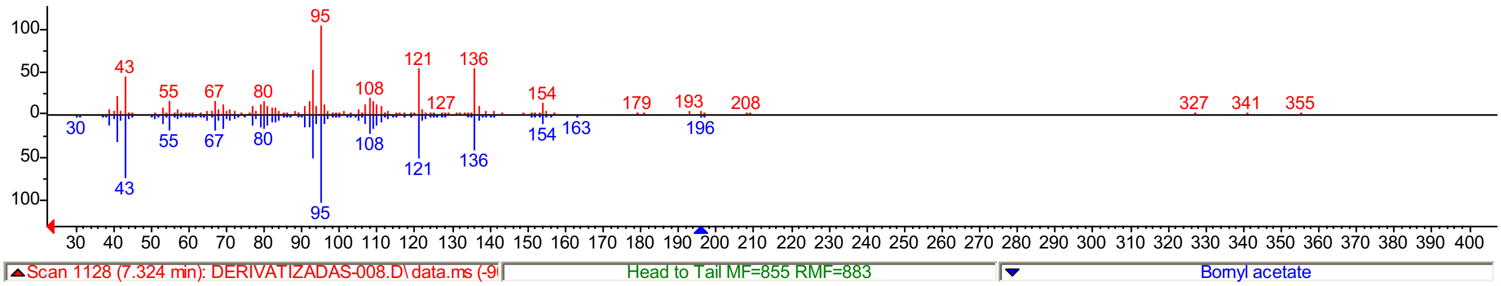

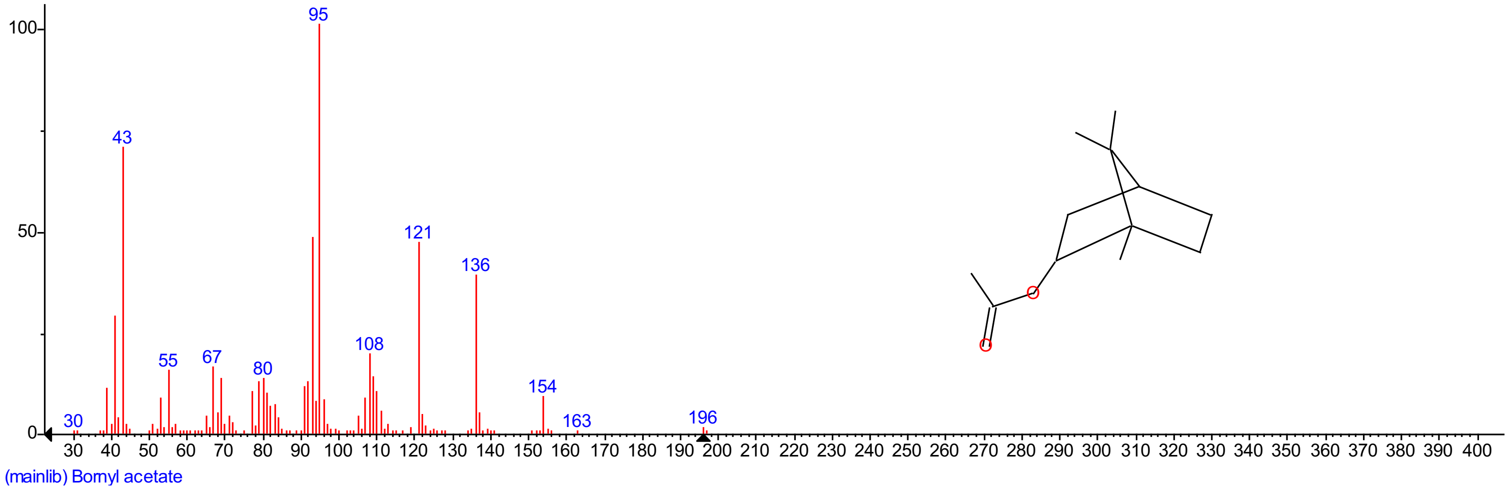

7.- α-Copaene

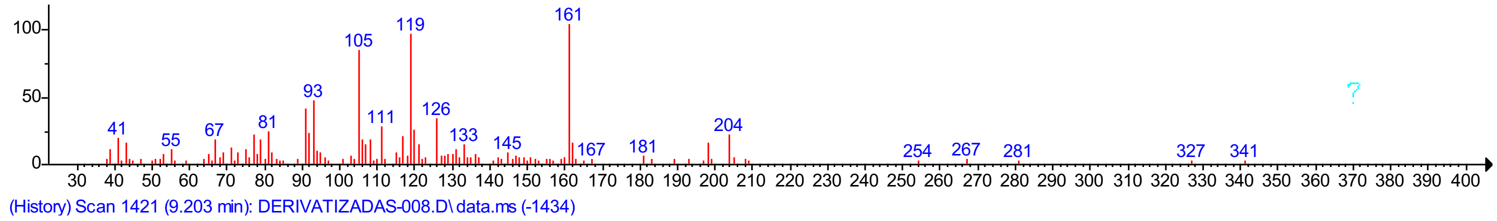
100
50
0
41 55 67 81 93 105 111 119 126 133 145 161 167 181 204 254 267 281 327 341
?
30 40 50 60 70 80 90 100 110 120 130 140 150 160 170 180 190 200 210 220 230 240 250 260 270 280 290 300 310 320 330 340 350 360 370 380 390 400
(History) Scan 1421 (9.203 min): DERIVATIZADAS-008.D\ data.ms (-1434)

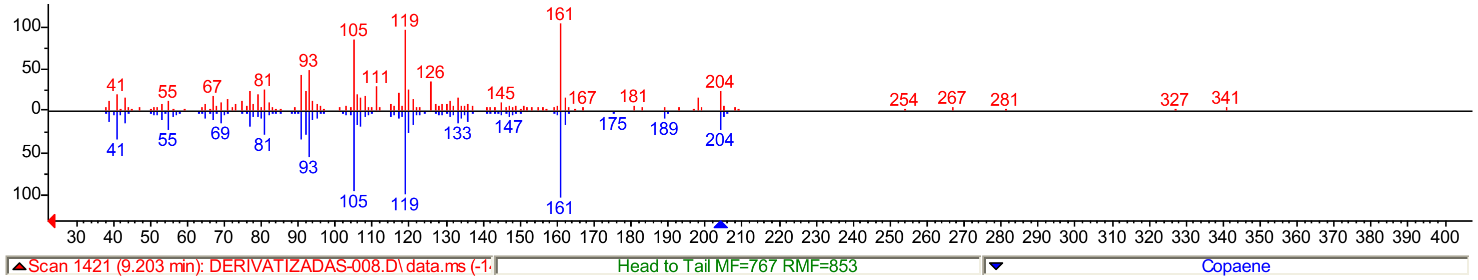
100
50
0
50
100
41 55 67 81 93 105 111 119 126 145 161 167 181 204 254 267 281 327 341
41 55 69 81 93 105 119 133 147 161 175 189 204
30 40 50 60 70 80 90 100 110 120 130 140 150 160 170 180 190 200 210 220 230 240 250 260 270 280 290 300 310 320 330 340 350 360 370 380 390 400
Scan 1421 (9.203 min): DERIVATIZADAS-008.D\ data.ms (-1
Head to Tail MF=767 RMF=853
Copaene

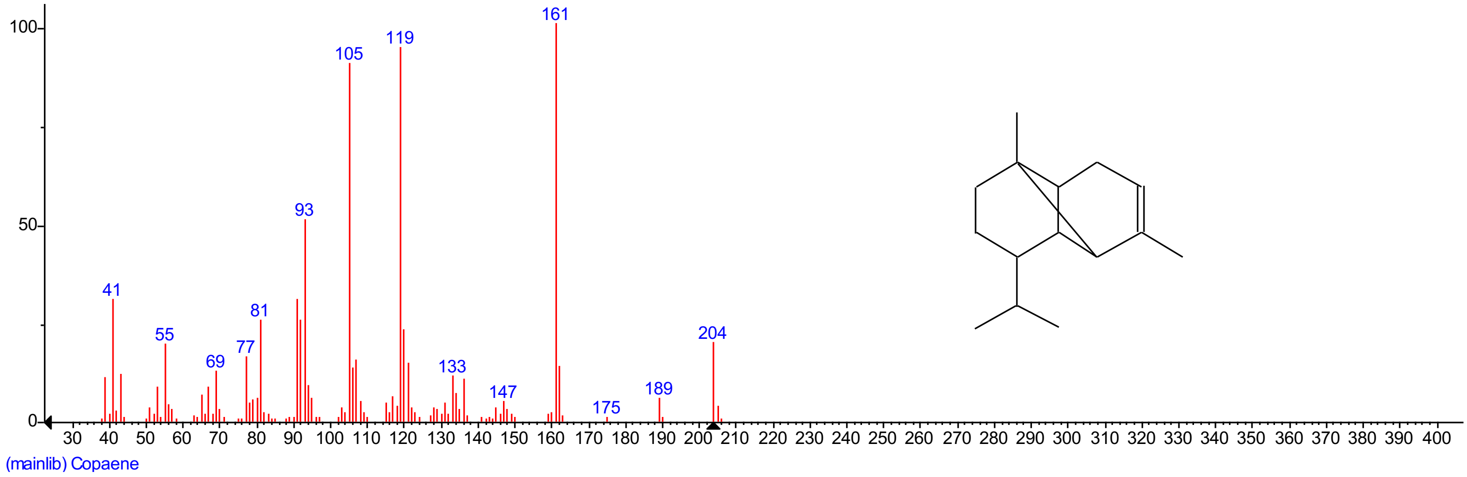
100
50
0
41 55 69 77 81 93 105 119 133 147 161 175 189 204
30 40 50 60 70 80 90 100 110 120 130 140 150 160 170 180 190 200 210 220 230 240 250 260 270 280 290 300 310 320 330 340 350 360 370 380 390 400
(mainlib) Copaene

8.- β-Caryophyllene

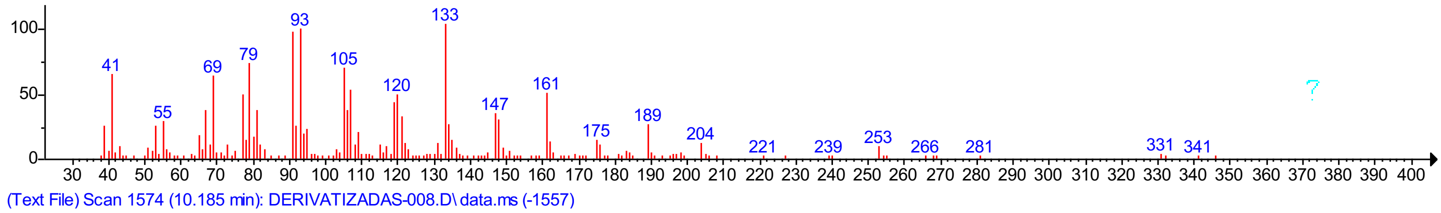

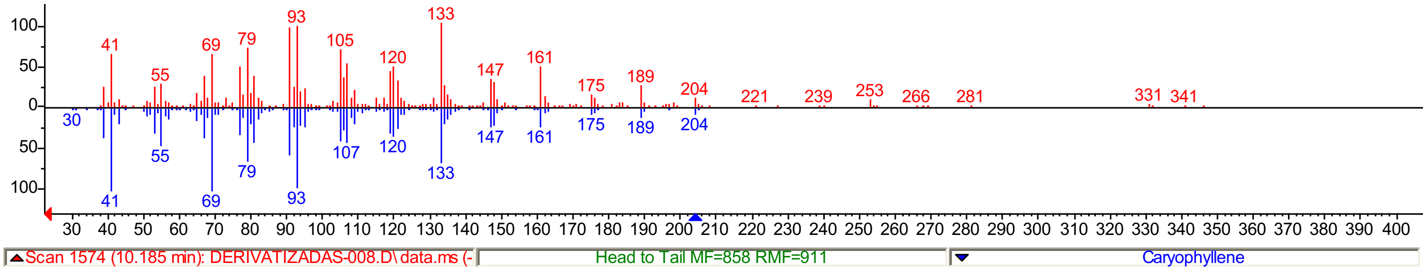

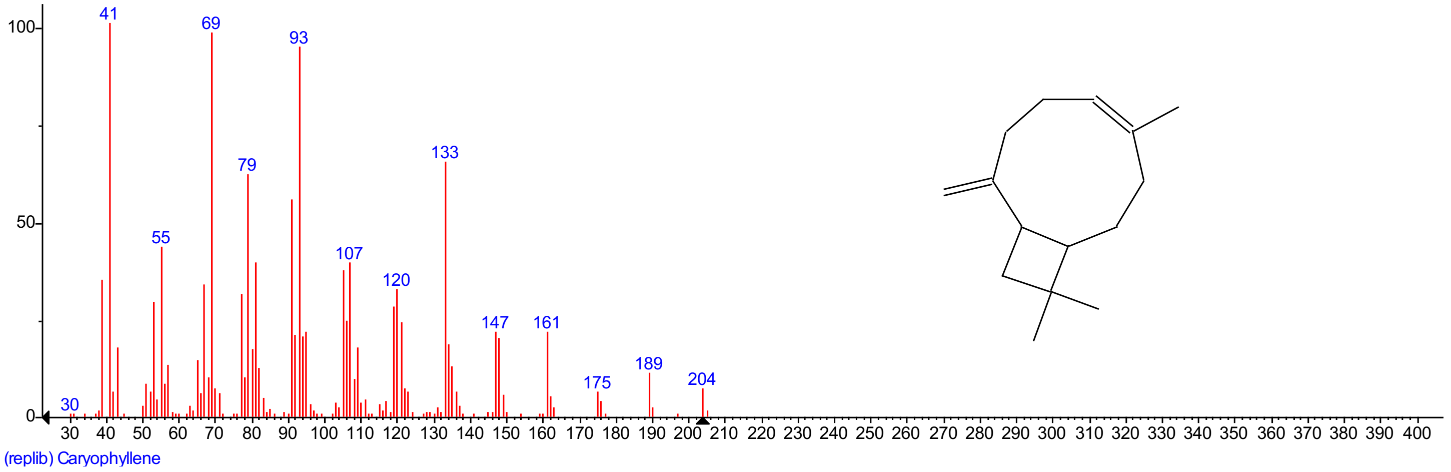

9.- α-Caryophyllene

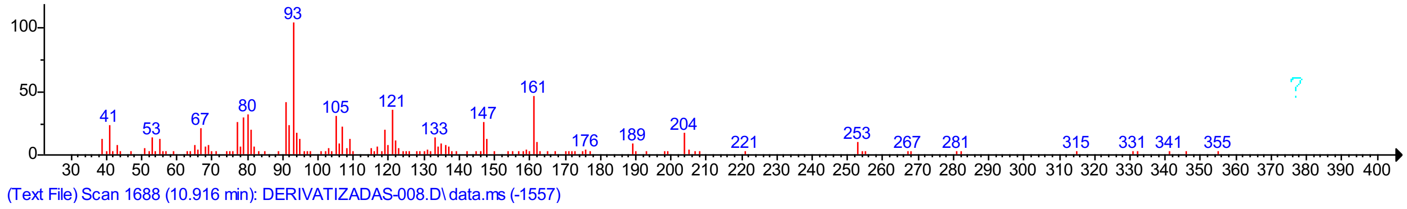

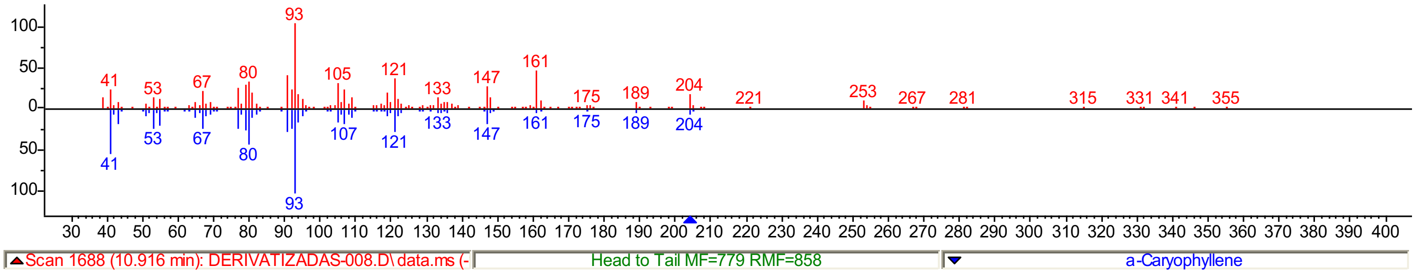

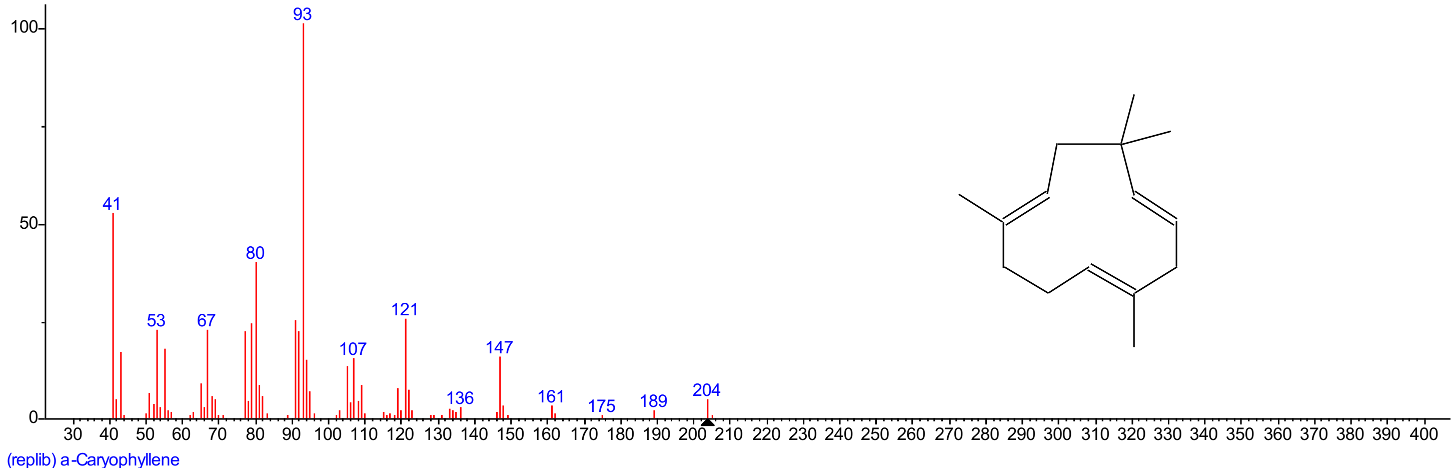

10.- Germacrene D

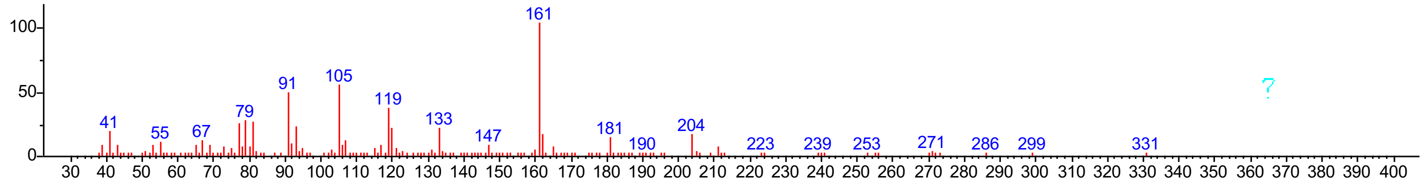

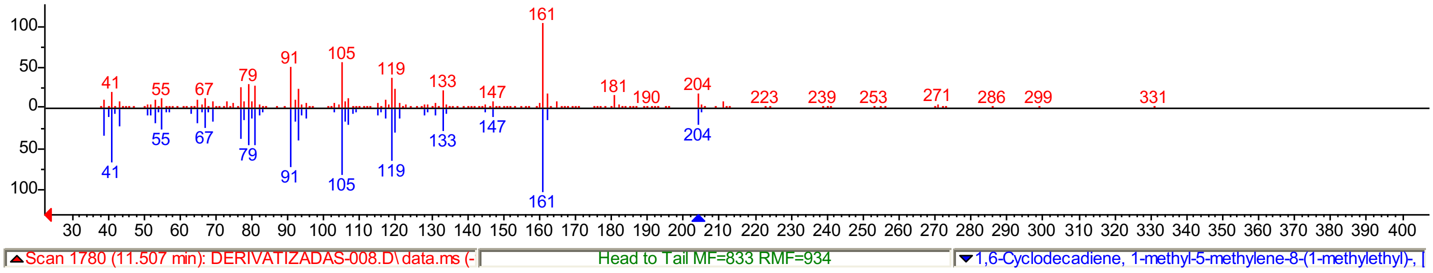

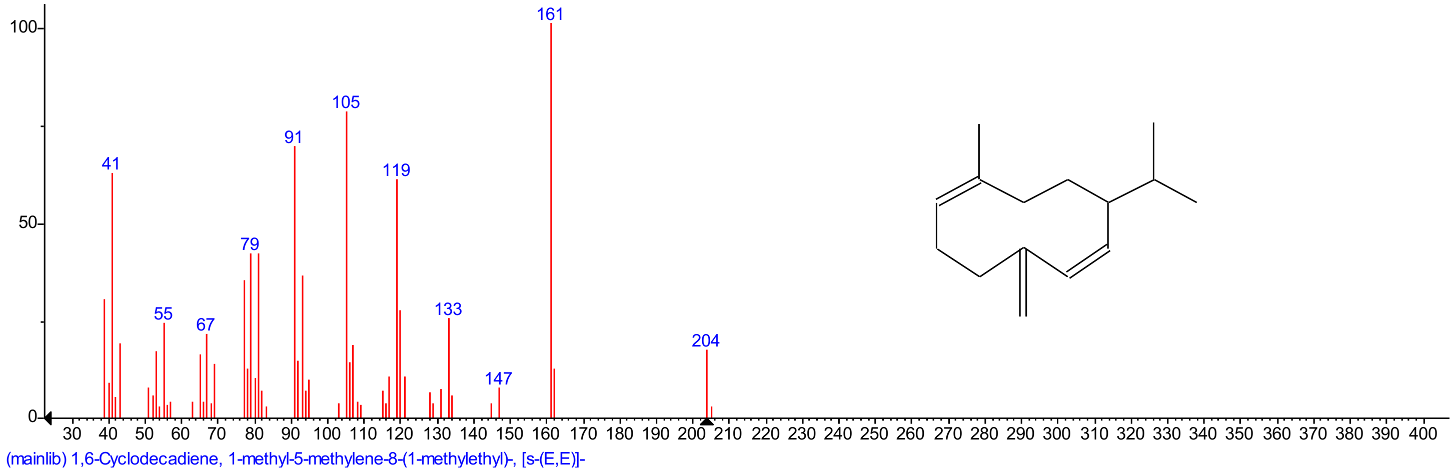

11.-Guaiazulene

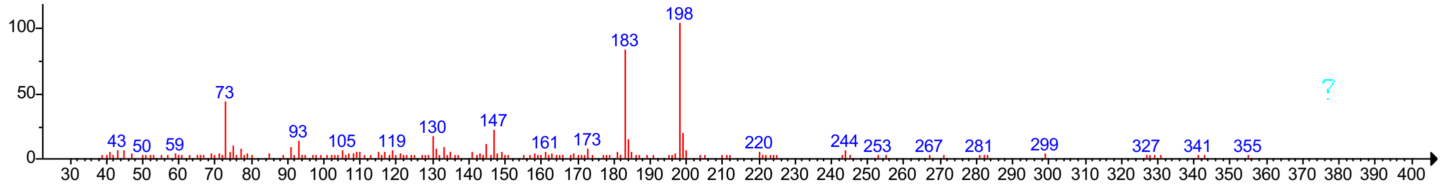

(Text File) Scan 1806 (11.673 min): DERIVATIZADAS-008.D\ data.ms (-1898)

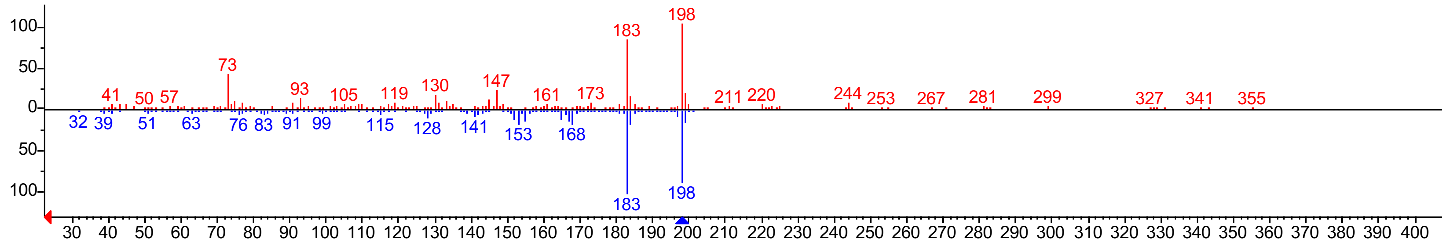

Scan 1806 (11.673 min): DERIVATIZADAS-008.D\ data.ms (- | Head to Tail MF=533 RMF=641 | Azulene, 1,4-dimethyl-7-(1-methylethyl)-

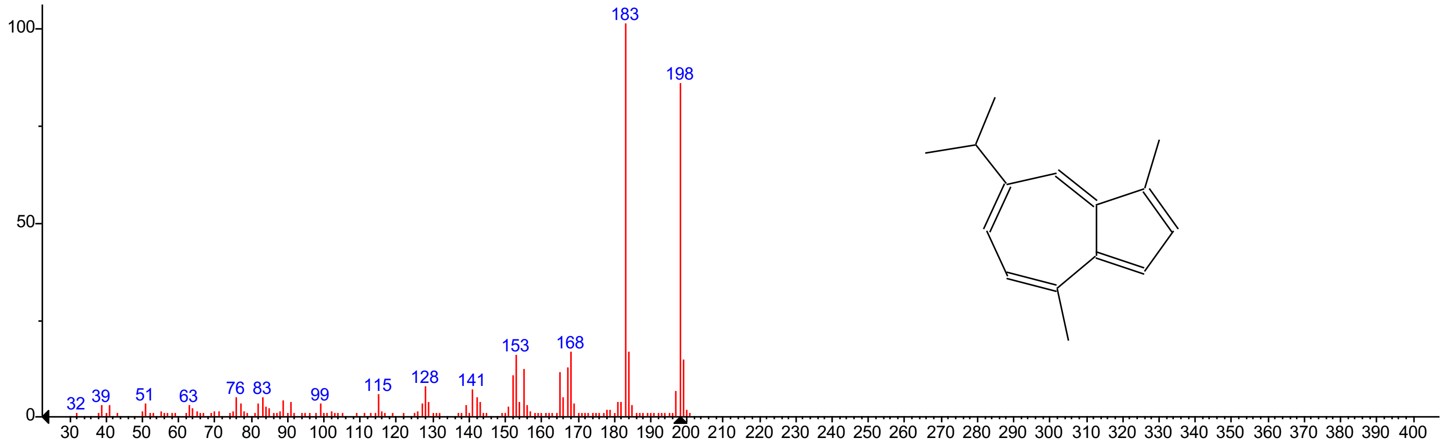

(replib) Azulene, 1,4-dimethyl-7-(1-methylethyl)-

12.- α-Muurolene

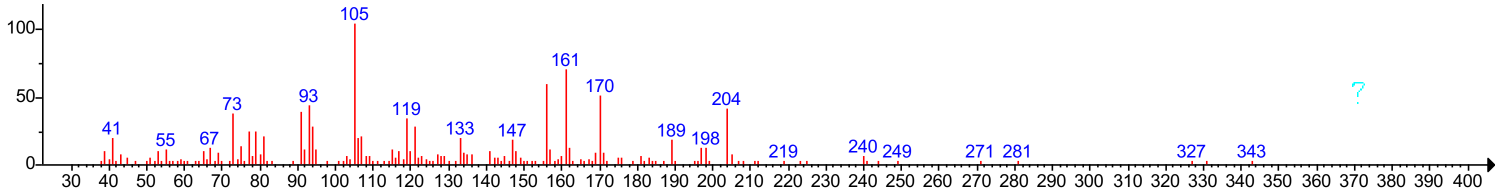

(Text File) Scan 1834 (11.853 min): DERIVATIZADAS-008.D\ data.ms (-1874)

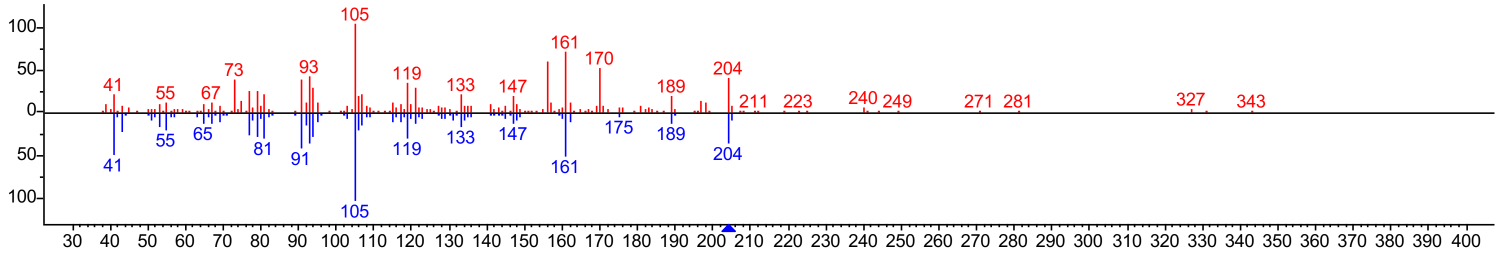

Scan 1834 (11.853 min): DERIVATIZADAS-008.D\ data.ms (-
Head to Tail MF=732 RMF=907
Naphthalene, 1,2,4a,5,6,8a-hexahydro-4,7-dimethyl-1-(1-meth

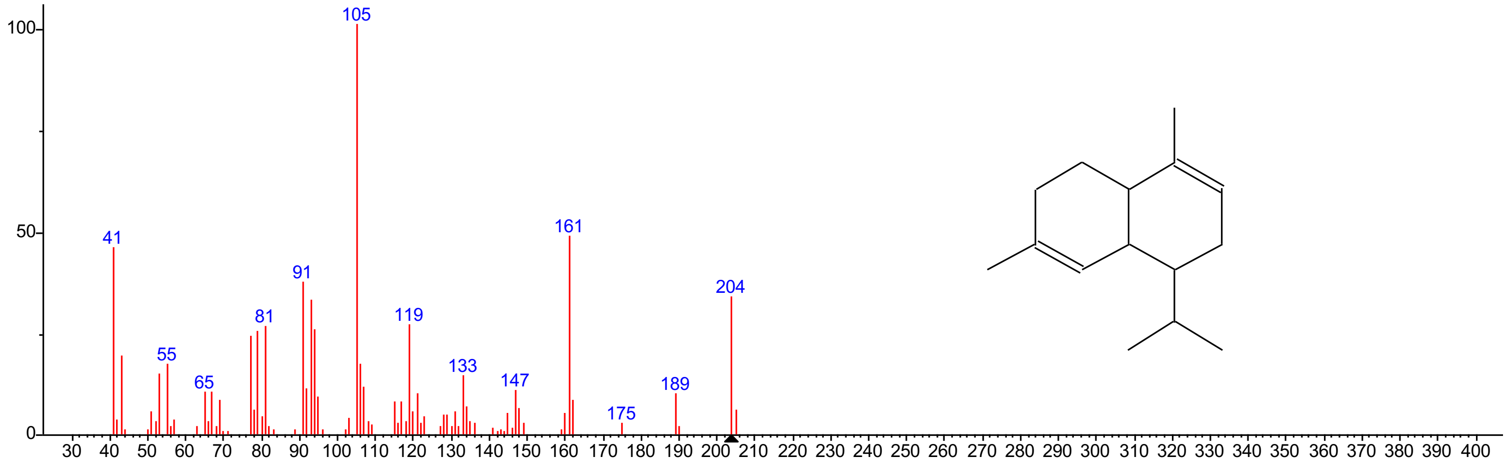

(replib) Naphthalene, 1,2,4a,5,6,8a-hexahydro-4,7-dimethyl-1-(1-methylethyl)-, (1a,4aa,8aa)-

13.- γ-Cadinene

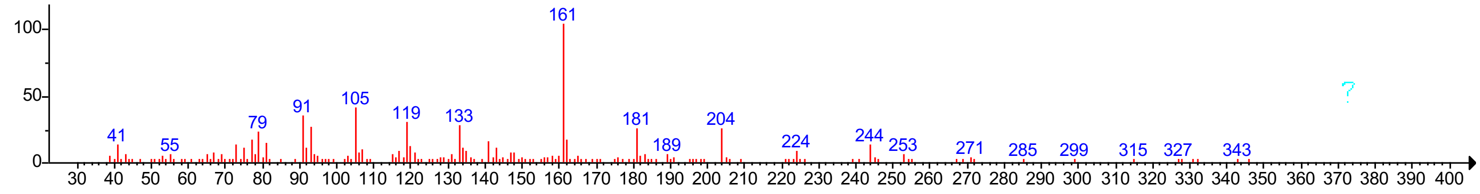

(Text File) Scan 1887 (12.193 min): DERIVATIZADAS-008.D\ data.ms (-1899)

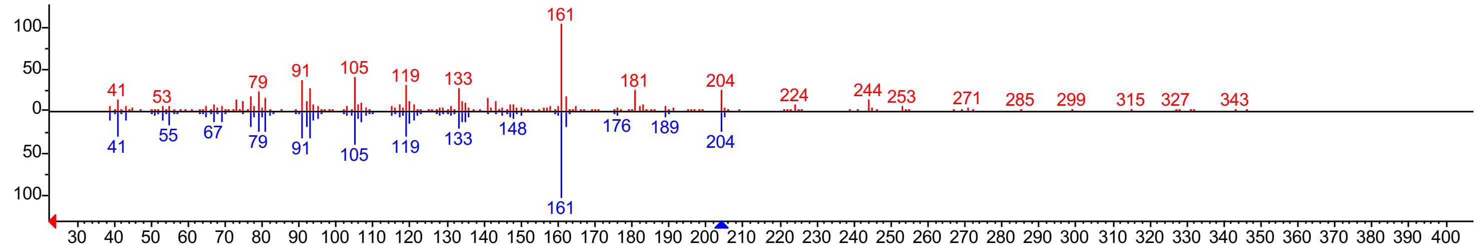

Scan 1887 (12.193 min): DERIVATIZADAS-008.D\ data.ms (- | Head to Tail MF=762 RMF=907 | Naphthalene, 1,2,3,4,4a,5,6,8a-octahydro-7-methyl-4-methyle

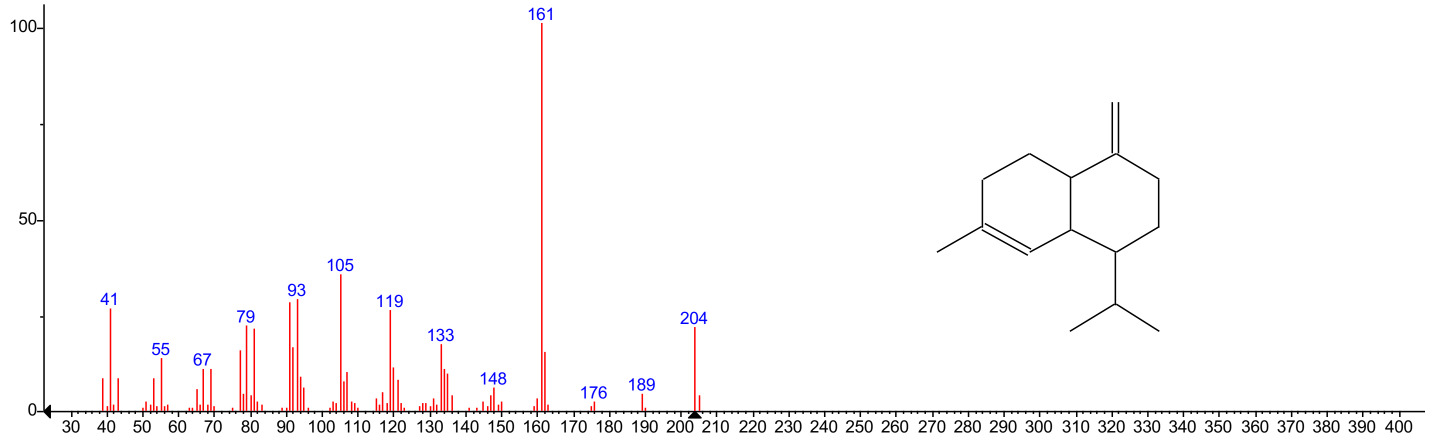

(replib) Naphthalene, 1,2,3,4,4a,5,6,8a-octahydro-7-methyl-4-methylene-1-(1-methylethyl)-, (1a,4aß,8aa)-

14.-δ-Cadinene

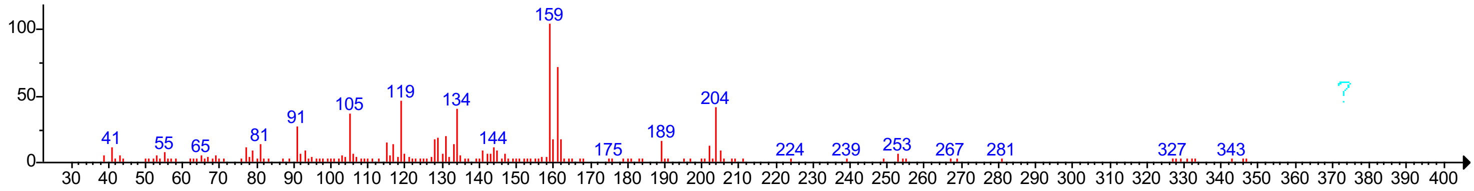

(Text File) Scan 1915 (12.373 min): DERIVATIZADAS-008.D\ data.ms (-1931)

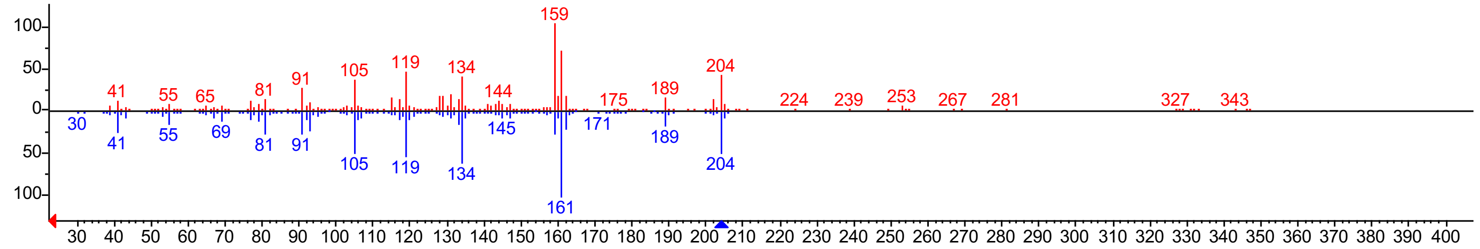

Scan 1915 (12.373 min): DERIVATIZADAS-008.D\ data.ms (- | Head to Tail MF=778 RMF=806 | Naphthalene, 1,2,3,5,6,8a-hexahydro-4,7-dimethyl-1-(1-methyl

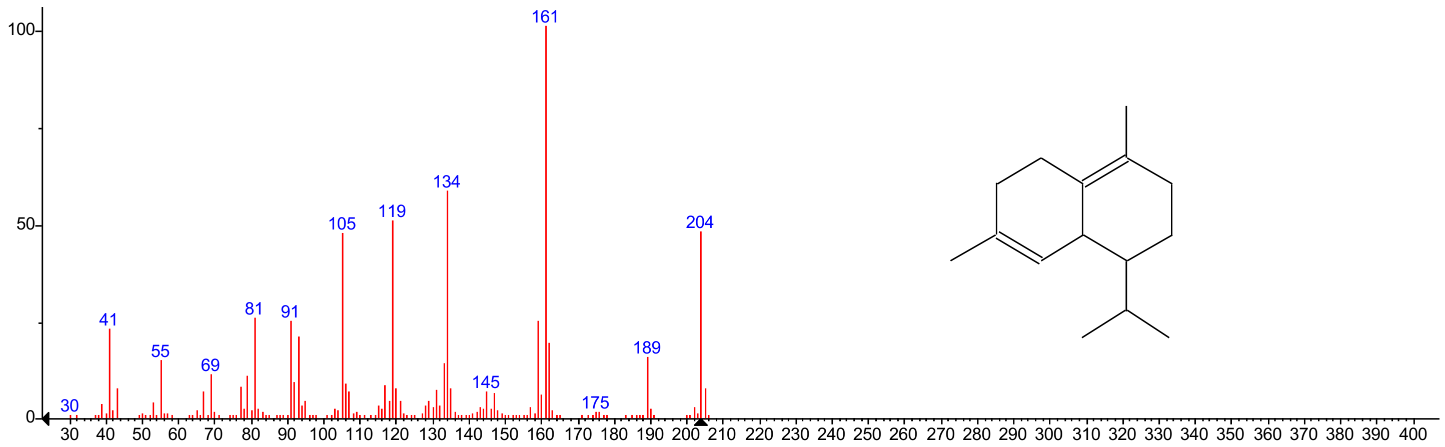

(replib) Naphthalene, 1,2,3,5,6,8a-hexahydro-4,7-dimethyl-1-(1-methylethyl)-, (1S-cis)-

15.-Caryophyllene oxide

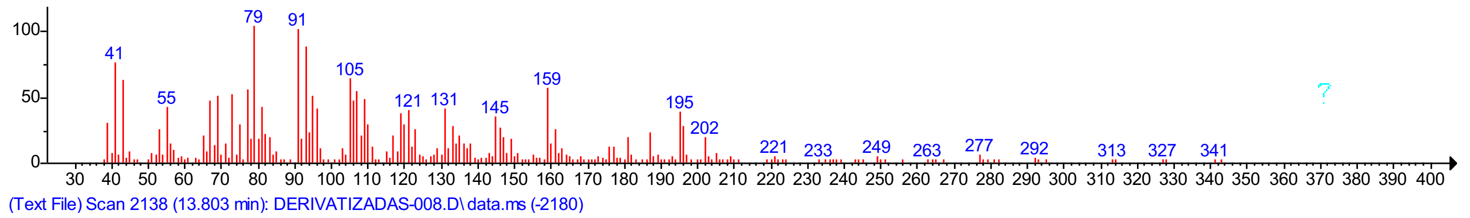

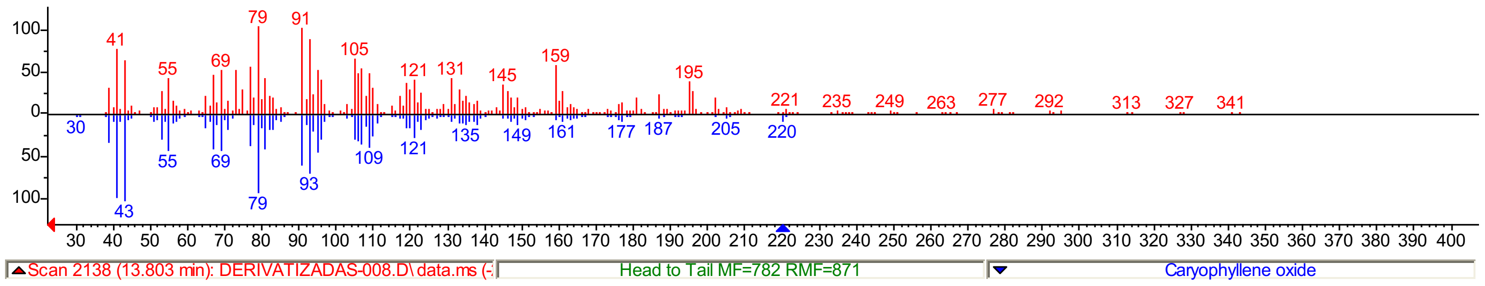

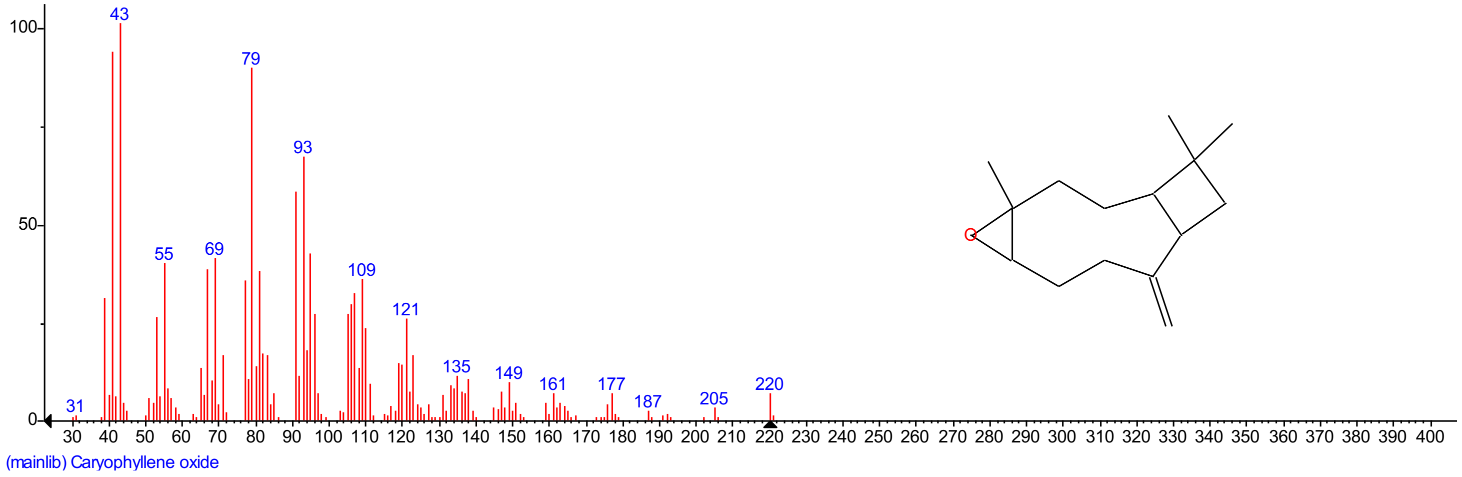

16.- β-Guaiene

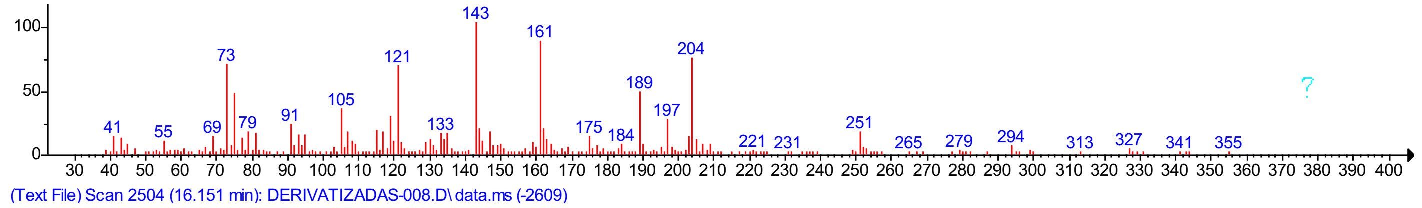
143 161 204 73 121 189 197 105 91 41 55 69 79 133 175 184 221 231 251 265 279 294 313 327 341 355
(Text File) Scan 2504 (16.151 min): DERIVATIZADAS-008.D\ data.ms (-2609)

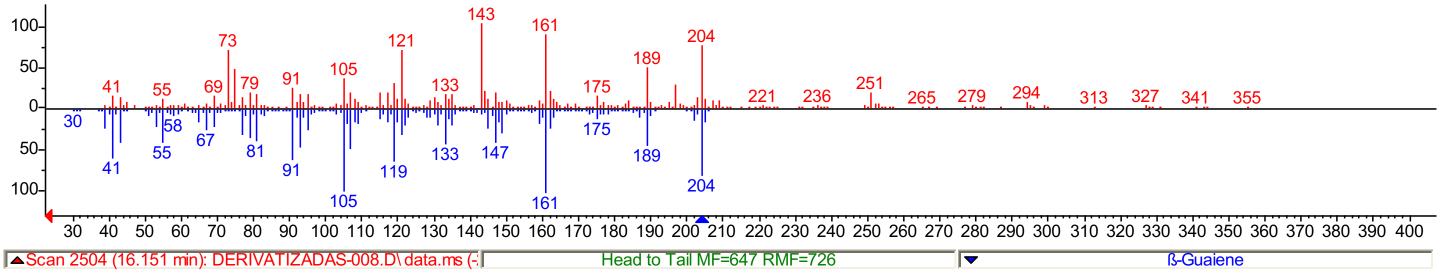
143 161 204 73 121 189 105 41 55 69 79 91 133 175 221 236 251 265 279 294 313 327 341 355
30 58 55 67 41 81 91 105 119 133 147 161 175 189 204
Scan 2504 (16.151 min): DERIVATIZADAS-008.D\ data.ms (-
Head to Tail MF=647 RMF=726
ß-Guaiene

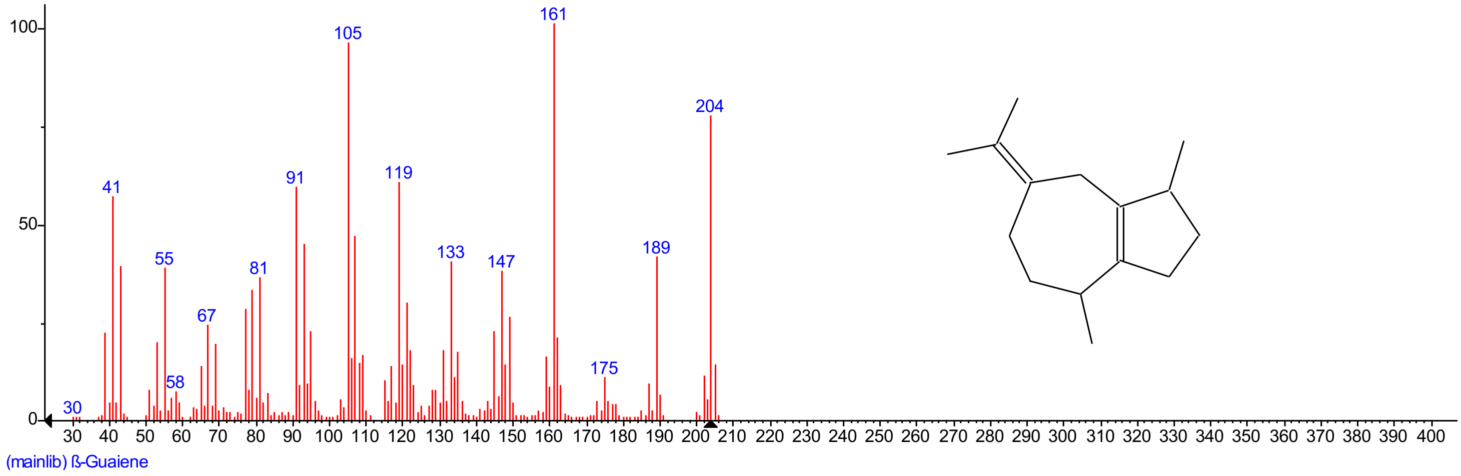
161 105 204 41 91 119 55 81 133 147 189 67 58 175 30
(mainlib) ß-Guaiene

17.- Valencene

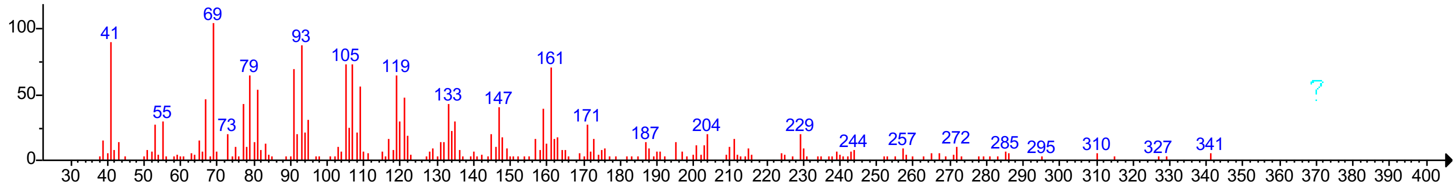

(Text File) Scan 3004 (19.359 min): DERIVATIZADAS-008.D\ data.ms (-2934)

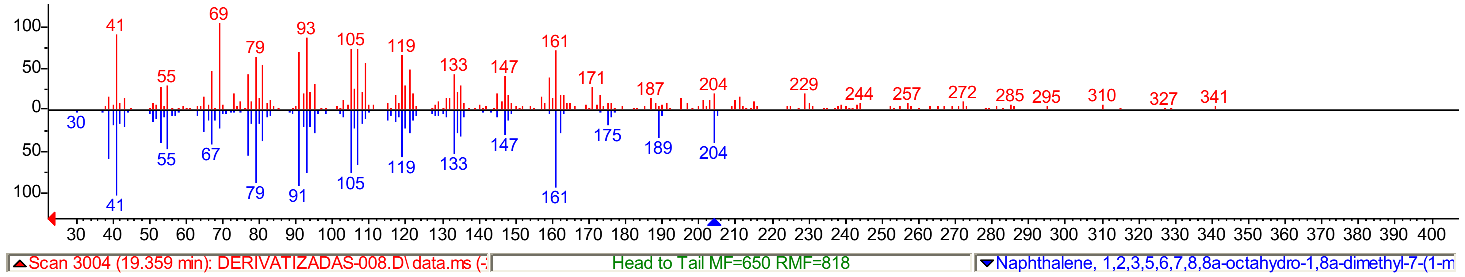

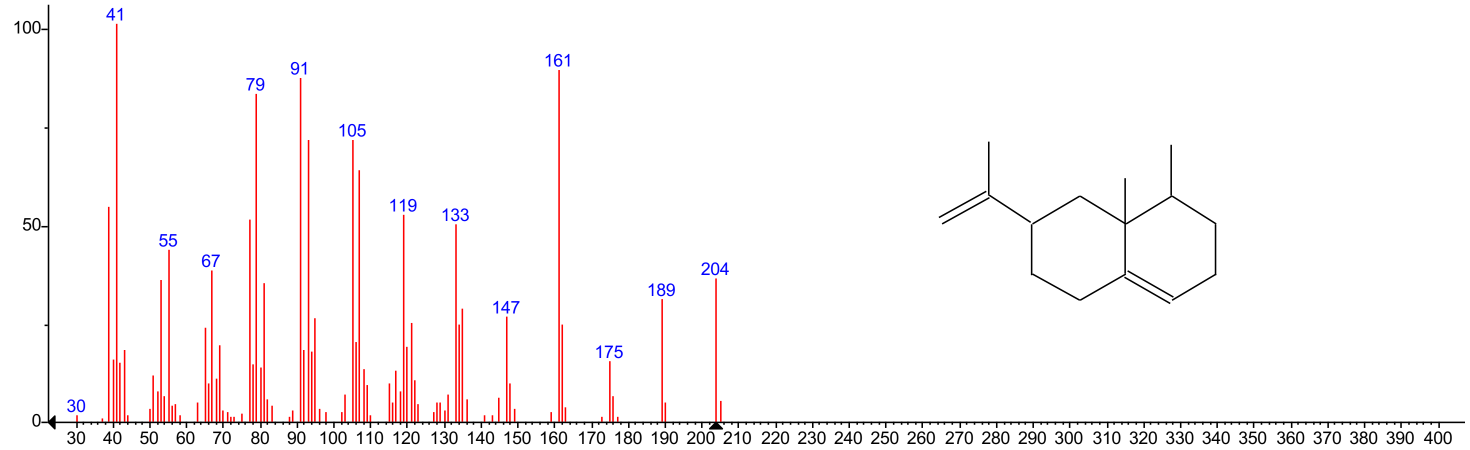

(replib) Naphthalene, 1,2,3,5,6,7,8,8a-octahydro-1,8a-dimethyl-7-(1-methylethenyl)-, [1R-(1a,7ß,8aa)]-

18.- Palmitic acid, ethyl ester

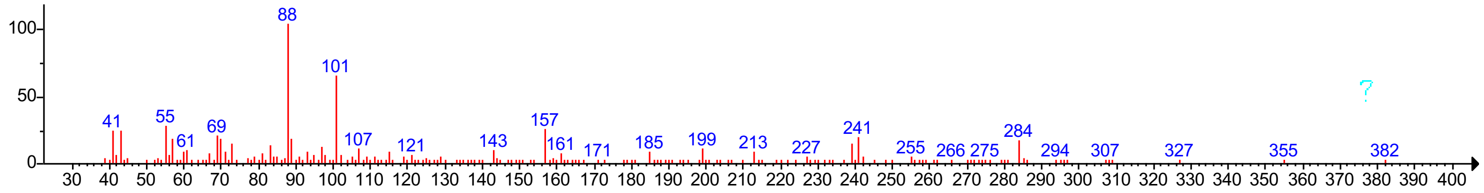

(Text File) Scan 3411 (21.970 min): DERIVATIZADAS-008.D\ data.ms (-3427)

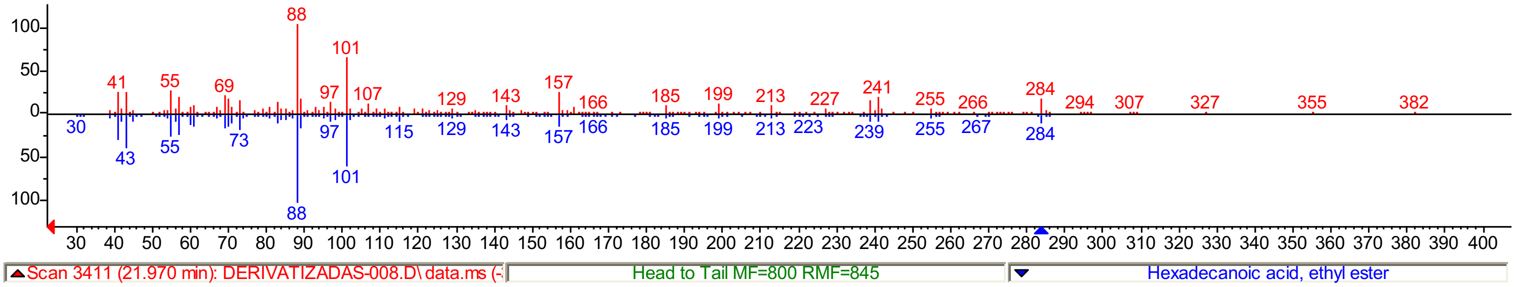

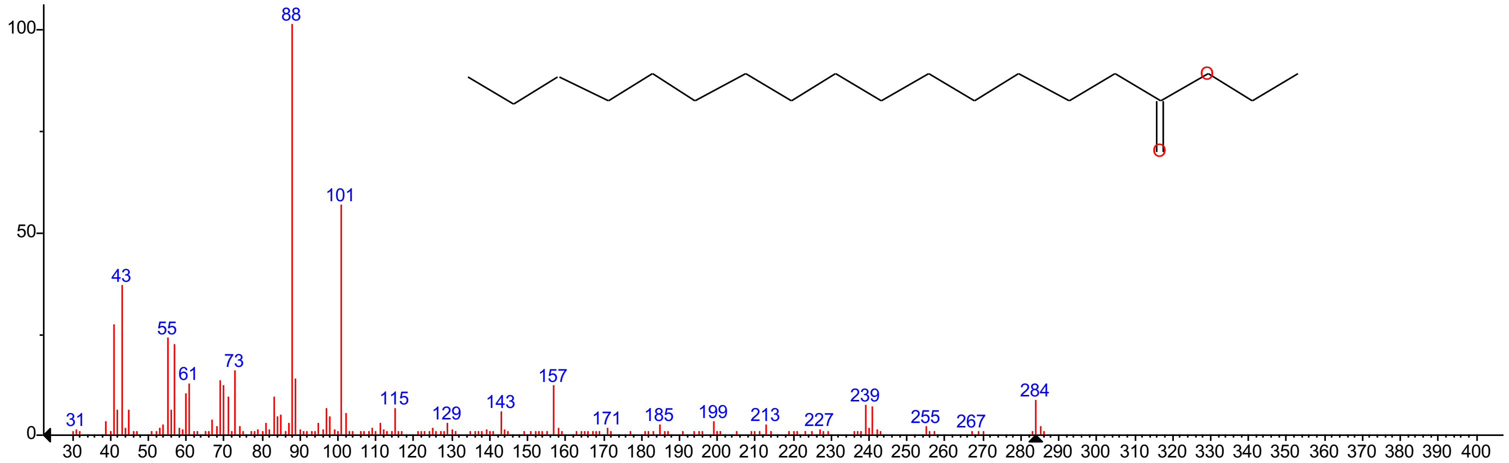

(mainlib) Hexadecanoic acid, ethyl ester

19.- Ent-kaurene

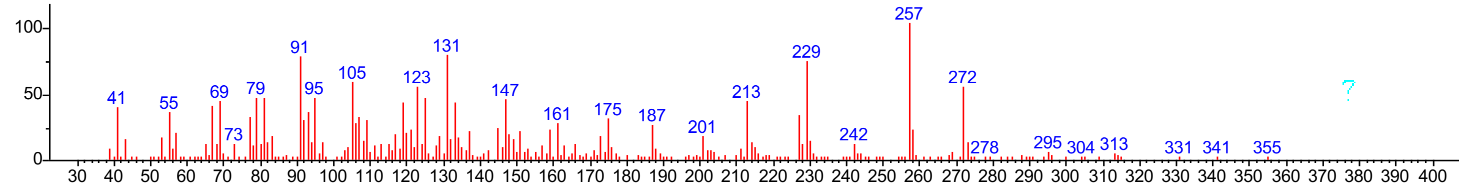

(Text File) Scan 3584 (23.080 min): DERIVATIZADAS-008.D\ data.ms (-3593)

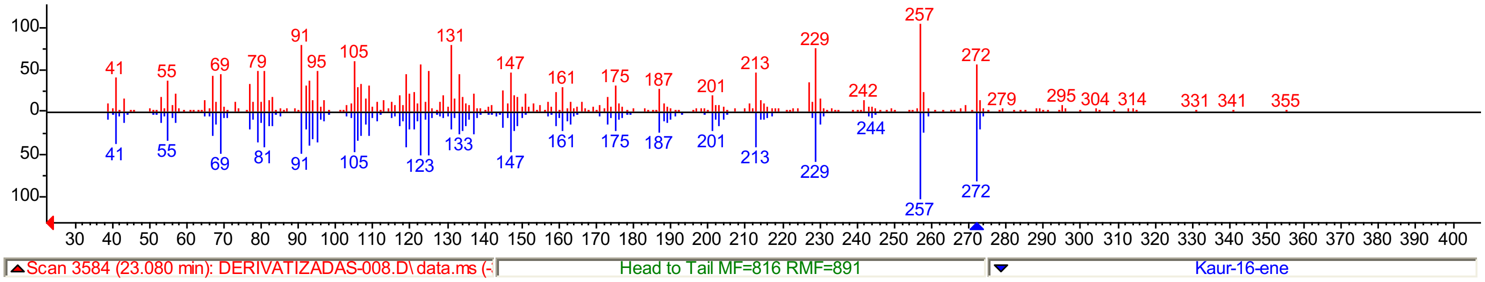

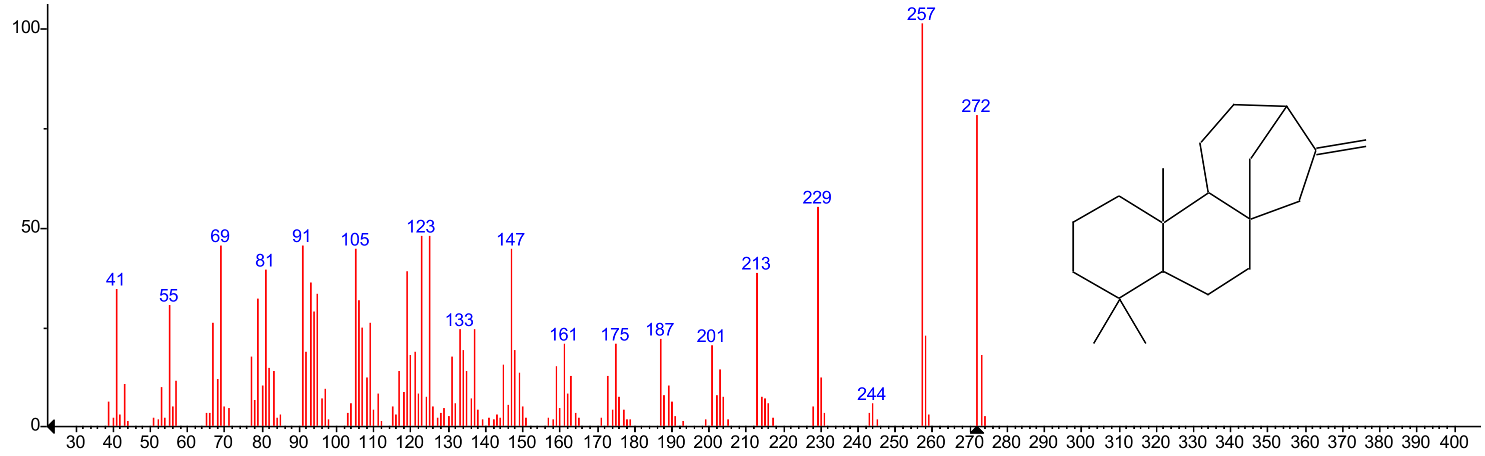

(mainlib) Kaur-16-ene

20.-Tricosane

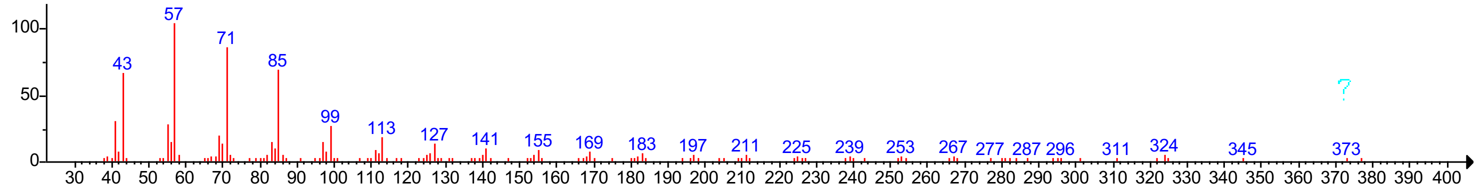

(Text File) Scan 4258 (27.404 min): DERIVATIZADAS-008.D\ data.ms (-4231)

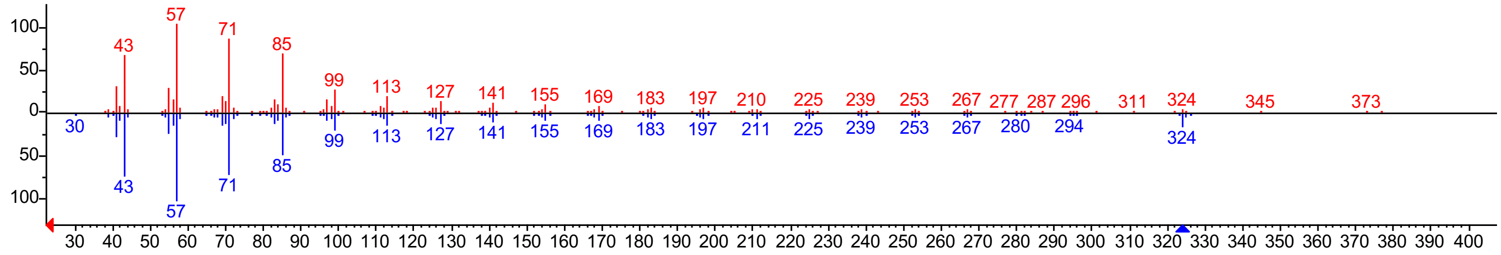

Scan 4258 (27.404 min): DERIVATIZADAS-008.D\ data.ms (-	Head to Tail MF=916 RMF=917	Tricosane

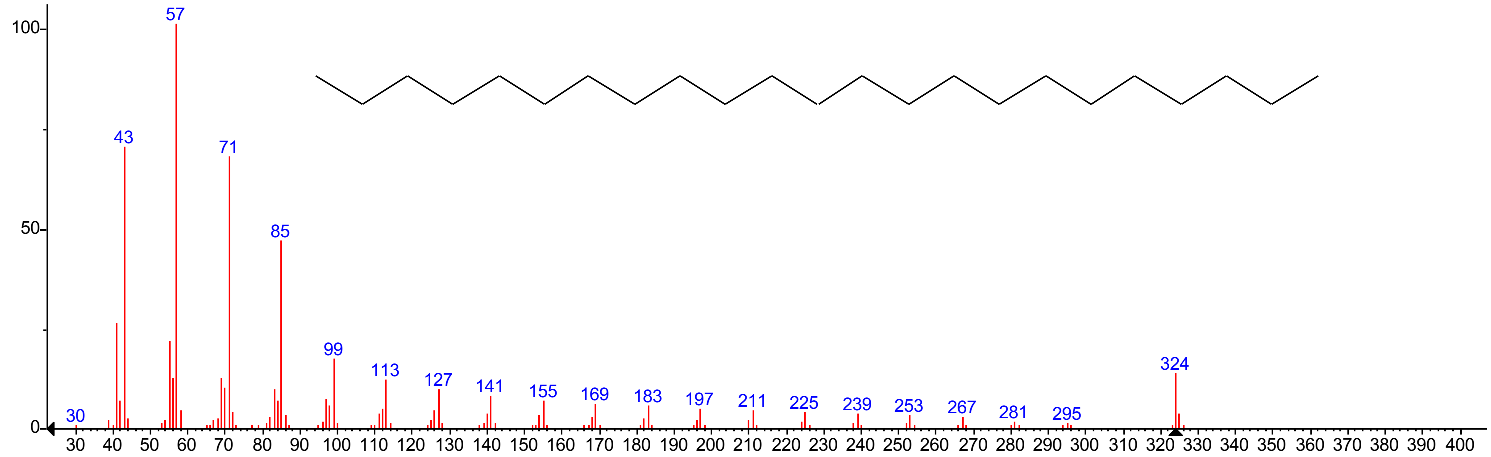

(mainlib) Tricosane
